# Methodological Choices on 24-h Movement Behavior Assessment by Accelerometry: A Scoping Review

**DOI:** 10.1186/s40798-025-00820-1

**Published:** 2025-03-13

**Authors:** Bruno Rodrigues, António Videira-Silva, Luís Lopes, Eduarda Sousa-Sá, Susana Vale, Dylan P. Cliff, Romeu Mendes, Rute Santos

**Affiliations:** 1 Faculty of Sport, University of Porto (Research Centre in Physical Activity, Health and Leisure), Porto, Portugal; 2 SPRINT Sport Physical Activity and Health Research and Innovation Center, 2040-413 Rio Maior, Portugal; 3 ESDRM Sport Sciences School of Rio Maior, Santarém Polytechnic University, 2040-413 Rio Maior, Portugal; 4 Programa Nacional Para a Promoção de Atividade Física, Direção-Geral da Saúde, Lisbon, Portugal; 5 CIDEFES (Centro de Investigação em Desporto, Educação Física e Exercício e Saúde), Universidade Lusófona, Lisbon, Portugal; 6 Laboratory for Integrative and Translational Research in Population Health, Porto, Portugal; 7 Early Start, School of Education, Faculty of the Arts, Social Sciences and Humanities, University of Wollongong, Wollongong, NSW Australia; 8 Polytechnic Institute of Porto, Porto, Portugal; 9 EPIUnit-Instituto de Saúde Pública, Universidade Do Porto, Porto, Portugal; 10 Northern Region Health Administration, Porto, Portugal; 11 Research Centre in Child Studies, University of Minho, Braga, Portugal; 12 Institute of Education, University of Minho, Braga, Portugal

**Keywords:** Sleep, Sedentary behavior, Physical activity, Time-use, Methods, Surveillance

## Abstract

**Background:**

There are no reviews describing current measurement protocols and accelerometer processing decisions that are being used in 24-h MovBeh studies, across the lifespan. We aim to synthesise information on methods for assessing 24-h movement behaviors using accelerometry across all age groups.

**Main Body:**

PubMed, PsycINFO, SPORTDiscus, and EMBASE were searched until December 2022. Observational or intervention reports describing accelerometry methods in studies on combinations of movement behaviors, with a 24-h protocol across all ages, were included. This review included 102 studies: three studies in toddlers, 15 in preschoolers, 17 in children, 23 in adolescents and 44 in adults and older adults. The Actigraph GT3X was the most commonly used device; the majority of the included reports collected data for seven days, including three weekdays and one weekend day, with a ≥ 16 h/day per 24-h period for valid data. The criteria for non-wear time varied between ≥ 20 and  ≥ 90 min of consecutive zero counts, depending on the age group. The most common epoch used was 15 or 60 s for youth and adults, respectively. The choice of sleep algorithms and SB/PA cut-points, of the included reports, depended on age and the original validation/calibration study. To deal with non-compliant participants, exclusion of non-compliant participants from the analysis was most frequently used. Most studies used diaries/logs to complement the accelerometer data.

**Conclusions:**

Accelerometer protocols and methodological decisions varied considerably between reports. Therefore, consensus on methodological decisions is needed to improve precision and comparability between studies, which is challenging given the complexity of the procedures, the number of available brands and types of accelerometers, and the plethora of programming options.

**Supplementary Information:**

The online version contains supplementary material available at 10.1186/s40798-025-00820-1.

## Key Points

- There are a wide variety of options available in terms of accelerometry methods for assessing 24-h movement behaviors.
- A consensus is needed to facilitate the homogenisation of these methods, so that studies using accelerometry become more comparable over time and between countries.

## Background

The health benefits of achieving an adequate distribution of all three 24-h movement behaviors (MovBeh) [i.e., adequate levels of sleep, sedentary behavior (SB) and physical activity (PA)] have been well documented [1–5]. It is also known that the association between one MovBeh and health, may depend on the levels of the other behaviors [6]. This is because, according to the field of time-use epidemiology, any change in one behavior must be at the expense of another or the other two, making these components of the day time-dependent. This has led to the development of several country-specific 24-h movement guidelines for different age groups [7–11], as well as World Health Organization (WHO) guidelines for young children [12]. The integration of all MovBeh within the new guidelines necessitates an adaptation of the current monitoring and surveillance tools to accurately measure 24-h MovBeh in an integrated manner. This is essential for the assessment of guideline adherence, health associations, correlates and determinants, as well as the effectiveness and efficacy of interventions to promote 24-h MovBeh [13].

MovBeh can be measured by device-based methods such as accelerometry, which generally have advantages over self-report measures because of their greater accuracy [14–22] and also because they can assess all three MovBeh concurrently over consecutive 24-h periods. Other objective instruments such as, doubly labelled water, direct and indirect calorimetry, and direct observation of PA and SB, and polysomnography for sleep are currently in use; however, these do not assess all three MovBeh over 24-h periods in free-living conditions, and each has several limitations [15, 23].

Despite the limitations of accelerometry (e.g., they do not correctly measure muscle-strengthening exercises), they can detect body (non)movement (i.e., amount and intensity), continuously and over extended periods of time in free-living conditions; therefore, they have become one of the preferred methods to assess 24-h MovBeh [24]. Nevertheless, data collection and processing methods depend on individual decisions made by researchers (e.g. accelerometer brand/model and device placement, cut points or algorithms applied, data reduction performed, criteria for non-wear time, number and criteria of valid days required, etc.), which are necessary to collect and transform the data into estimates of MovBeh [23], thus leading to different results between studies [25]. To solve this issue, researchers are increasingly using original raw data as a basis for outcome calculations, leveraging software that can process and analyze acceleration data using diverse open-source methods. This approach has the potential to facilitate data harmonization and consistency across studies [26].

There are unique challenges in standardizing 24-h accelerometry data and procedures, such as distinguishing between SB, sleep and non-wear time, and the best place on the body to attach the accelerometer. It is therefore important to standardize methodological decisions using consensus guidelines for the collection and processing of accelerometer data. This is because different methodological choices for processing the same data can result in significantly different values for the same variables. The lack of standardization poses significant limitations, particularly in the context of large-scale epidemiological studies. These include issues related to generalizability, validity and sustainability, which are essential for ensuring comparability of results across studies [25]. In addition, questionnaires are still the most widely used form for recording MovBeh, given their practicality, simplicity, affordability, and low burden for participants [15, 20, 27]. Moreover, these are capable of gathering valuable contextual information (e.g., domains, settings, types) of the behaviors, that accelerometry is unable to [28]. For these reasons, questionnaires such as the 24-h Movement Questionnaire (QMov24h) [29], continue to be developed and validated, to assess movement behaviors per se or as a complement to accelerometry.

There is an overwhelming amount of information on accelerometry methodologies. Several reviews have summarized accelerometer measurement properties [30–32], analytical approaches [33], best practices in selecting and applying accelerometers to quantify the 24-h MovBeh [24, 34], methodological choices (e.g., epoch length, wear site, cut-points) for assessing PA in young children [35] and MovBeh in elderly [36], or across the lifespan, but were specific to a particular brand and focused on validation and calibration studies [27]. However, none of the previous reviews described current measurement protocols and accelerometer processing decisions to be used in 24-h MovBeh studies, across the lifespan. Therefore, we aim to synthesize the information on the methods used to assess 24-h movement behaviors using accelerometers, across all age groups.

## Main Text

### Methods

This review is reported in accordance with the PRISMA extension for Scoping Reviews guidelines [37]. The protocol is publicly available in the Open Science Framework repository (OSF Repository Files—10.17605/OSF.IO/GHCSY). The current scoping review is consistent with the methodology proposed by Arksey and O'Malley [38] and later updated by Levac et al. [39]. Scoping reviews have been given little attention when compared with other types of reviews, such as systematic reviews. Nevertheless, scoping reviews might be more appropriate when the research question is broader, and the aim does not comprise the evidence systematizing nor the studies quality assessment, such as in this study [39].

#### Eligibility Criteria

Inclusion criteria: (1) reports with babies, toddlers, children, adolescents, adults and older adults, non-pregnant, without disease at baseline, in free-living conditions; (2) observational and intervention reports that reported accelerometry methods for MovBeh combinations (sleep, SB, PA); (3) Studies in which participants were asked to wear accelerometers 24 h per day; (4) reports written in English, Spanish, French, Portuguese, German, or Italian.

Exclusion criteria: (1) reports conducted solely with clinical populations (e.g., established chronic disease(s), motor diseases, auto-immune diseases, infectious diseases, sleep disorders, cognitive impairment, etc.); (2) reports evaluating just one or two MovBeh; (3) reports evaluating just one or two MovBeh with accelerometry and the remaining with another method, such as self-report; (4) grey literature (e.g. theses, book chapters), case-reports, editorials, conference abstracts, methods papers, reviews, meta-analyses, validation reports, commentaries, cost-effectiveness reports and qualitative reports; (5) when multiple reports were published based on the same study and used the same accelerometer procedures, we included the study with the largest sample size.

#### Information Sources and Search Strategy

Four electronic databases were searched from inception to December 2022: PubMed, EMBASE, PsycINFO and SPORTDiscus. Additional reports were identified by manually searching references from the included reports and authors’ personal libraries.

The databases were searched for variants of the following terms: ‘movement behaviors’, ‘sleep’, ‘sedentary behavior’, ‘physical activity’, ‘accelerometer’ and ‘measurement properties’ (for a detailed search strategy see Additional file 1). The search terms were adjusted for each specific electronic database to ensure the quality of the systematic searching.

#### Study Selection Process

Two authors (BR; AVS) independently selected potentially relevant reports based on titles, abstracts, and full texts. Disagreements were discussed with a third author (RS). The study selection process was conducted through CADIMA software [40].

#### Data Charting Process

A standardized data extraction sheet was built to chart relevant information from the included reports. It included country, study name, type of study, participants’ age and sex and corresponding results: accelerometer brand/model; accelerometer placement on the body; software used for data reduction and analysis; number of days for data collection; number of required valid days, and time required to be considered as a valid 24-h period; non-wear time criteria; epoch length and data reduction (epoch integration); sampling rate (i.e., hertz); MovBeh outcome (i.e., sleep, light PA and moderate-to-vigorous PA, etc.) and reporting formats (e.g., minutes/day, steps/minutes, etc.); sleep measuring procedure and PA and SB cut-points used; procedures for identifying and handling non-compliant participants; log or diary use.

The data were collected independently by 2 authors (BR; AVS) and disagreements were resolved by discussion with another author (RS).

#### Synthesis of Results

A high-level summary of findings table was created to present total counts of each variable collected in all reports. Additionally, two narrative sub-analyses were carried out: (1) the most used procedures, and (2) the most used cut-points and algorithms depending on the body placement and the accelerometer brand.

### Results

#### Search Results

**Fig. 1:**
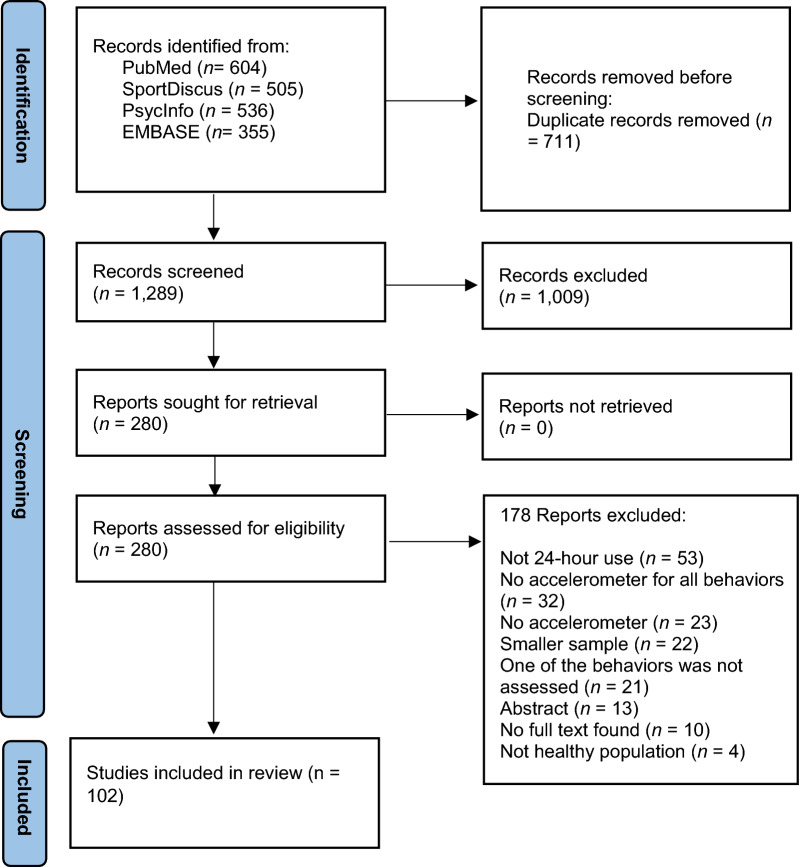
PRISMA Study Selection Process Flowchart

The search yielded 2000 records after removing duplicates. Of these, 1720 were excluded based on title and abstract, leaving 280 records for full-text screening. One hundred and two reports met eligibility criteria and were included. The flow diagram and the reasons for full texts’ exclusion are described in Fig. 1.

#### Reports’ Characteristics

Of the 102 reports included, 72 were cross-sectional, 21 longitudinal, 6 RCTs and 3 non-RCT interventions. Overall, this review included 140,643 participants (~ 55% female). Ninety-four reports included both sexes, seven reports included only girls/women and one study included only boys/men. Regarding the age groups, this review included three reports with toddlers [41–43], 15 reports with preschoolers [44–58], 17 reports with children [59–75], 22 reports with adolescents [76–96] and 44 reports with adults and older adults [97–141]. The most represented countries were the United States of America (*n* = 32), Australia (*n* = 10) and Spain (*n* = 9). The summary of reports’ characteristics is presented in Table 1.

**Table 1:** Characteristics of reports

Characteristics	Number of reports
*Study design*	
Cross-sectional	72
Longitudinal	21
RCT	6
Other interventions’ designs	3
*Sample size* (*n = 140,643*)	
< 100	26
100–499	48
500–1000	15
> 1000	13
*Participants*	
Sex (~ 55.23% girls/women)	
Both sexes	94
Boys/Men only	1
Girls/Women only	7
*Age*	
Toddlers (2–3 years)	3
Preschoolers (3–5 years)	15
Children (5–10 years)	17
Adolescents (10–17 years)	23
Adults and older adults (≥ 18 years)	44
*Countries*	
USA	32
Australia	10
Spain	9
UK	8
Canada	6
New Zealand	6
Brazil	5
Finland	5
China	4
Denmark	4
Belgium	2
Chile	2
Japan	2
Singapore	2
South Africa	2
Czech Republic	1
Colombia	1
Germany	1
Netherlands	1
Portugal	1
Slovenia	1
Switzerland	1

#### Synthesis of the Results

The results of each report, by age group, are presented in Additional file 2 and the high-level summary of findings on accelerometer methods and procedures are presented in Table 2.

**Table 2:** High level summary of findings on accelerometer methods and procedures

	Toddlers and preschoolers (2–5 years)	Children (5–10 years)	Adolescents (10 to 17 years)	Adults and older adults (≥ 18 years)
**Accelerometer model and device placement**	Actigraph GT3X on hip (7/18) [42, 45, 48–50, 56, 57], on wrist (2/18) [43, 46] Actical on hip (2/18) [51, 53], on ankle (1/18) [41] GENEActiv tri-axial on wrist (1/18) [44] ActivPAL on anterior tight midway and Axivity on wrist (1/18) [47] Actiwatch Spectrum on wrist (1/18) [52] ActiGraph GT3X on hip and Actiheart on chest (1/18) [54] ActiGraph wGT3X on hip and activPAL4 on the thigh (1/18) [55] ActiGraph GT9X on hip (1/18) [58]	ActiGraph wGT3X on hip (6/17) [59, 62, 63, 65, 66, 69], on wrist (2/17) [61, 70] GENEActiv on wrist (2/17) [71, 72] FitBit Charge 2 on wrist (2/17) [64, 67] Actiwatch 4 on wrist (1/17) [60] ActiGraph GT9X on wrist (1/17) [68] Axivity AX3 on thigh and lower back (1/17) [73] ActiGraph GMTI on hip for PA, and on wrist for Sleep (1/17) [74] Actical on hip (1/17) [75]	ActiGraph GT3X on hip (5/23) [76, 78, 90, 92, 145] GENEActiv on wrist (4/23) [77, 79, 89, 91] Actical on hip (1/23) [86], on wrist (1/23) [88] Actiwatch on hip (1/23) [93], on wrist (1/23) [96] ActiGraph GT9X on wrist (2/23) [82, 85] ActiGraph GT3X on hip for SB and PA and SleepWatch on wrist for Sleep (2/23) [80, 82] Actiheart on chest (1/23) [83] ActiGraph GT9X Link or wGT3X on wrist (1/23) [84] ActivPAL4 micro on anterior thigh (1/23) [87] ActiGraph wGT3x on hip for SB and PA, and SleepWatch on wrist for sleep (1/23) [94] ActiGraph wGT3x on hip for SB and PA, and Actiwatch on wrist for sleep (1/23) [95]	ActiGraph GT3X on wrist (6/44) [104, 110, 125, 132, 137, 141], on hip (2/44) [105, 107], on thigh (2/44) [120, 128] GENEActiv on wrist (5/44) [101, 114, 121, 123, 127] ActiGraph GT3X on waist for PA and on non-dominant wrist for sleep (5/44) [118, 129, 130, 140, 181] ActivPAL3 on thigh (2/44) [119, 126], on midline anterior aspect of the upper thigh (2/44) [99, 139] ActiGraph GT9X on wrist (3/44) [98, 103, 135] SenseWear Pro 3 Armband on arm (3/44) [117, 134, 136] SenseWear Mini Armband on the upper arm (2/44) [106, 112] Silmee on wrist (1/44) [97] Actiwatch-64 on wrist and the Actical on waist (1/44) [100] Fitbit on wrist (1/44) [108] Axivity AX3 on wrist (1/44) [109] ActiGraph GT3X on hip for SB and PA and ActiGraph GT9X on wrist for sleep (1/44) [111] ActiGraph GT3X for SB and PA and ActiSleep-BT for sleep on wrist (1/44) [113] IDEEA pattern-recognition activity monitor on waist (1/44) [115] UKK RM42 on hip for SB and PA and on wrist for sleep (1/44) [116] Actiwatch Spectrum on wrist (1/44) [124] ActiGraph GT3x on hip for SB and PA and Actiwatch2 on wrist for sleep (1/44) [130] ActiGraph GT1M on hip for SB and PA, and Actiwatch-2 on wrist for sleep (1/44) [133] Omrom Active Style Pro HJA-350IT on hip for PA and SB and actigraph on wrist for sleep (1/44) [138]
**Nº of days for data collection**	7 days (9/18) [42, 43, 45–47, 50, 51, 54, 57] 5 days (3/18) [49, 53, 56] 3 days (2/18) [48, 58] 3–7 days (1/18) [41] 6 days (1/18) [44] 16 days (1/18) [52] 4 days (1/18) [55]	7 days (11/17) [59–61, 63, 65, 66, 68–70, 73, 74] 8 days (3/17) [62, 67, 71] 6 weeks (1/17) [64] 6 days (1/17) [72] 3 days (1/17) [75]	7 days (12/23) [78, 80, 82, 84, 86, 88, 91, 92, 94–96, 145] 8 days (3/23) [76, 79, 90] N.R. (2/23) [89] 3 days (2/23) [85, 93] 6 days (1/23) [77] 5 days (1/23) [82] 4 days (1/23) [83] 14 days (1/23) [87]	7 days (27/44) [97, 99, 103–105, 109–111, 113, 114, 116–119, 121–126, 129, 131, 133–135, 138, 139, 141] 14 days (3/44) [100, 127, 140] 10 days (2/44) [106, 136] N.R. (2/44) [107, 108] 4 days (2/44) [128] 8 days (2/44) [130, 137] 2 days (1/44) [115] 3 days (1/44) [101] 1 days (1/44) [112] 25 days (1/44) [98] 5 days (1/44) [120] 2, 7 and 15 days (1/44) [132]
**Nº of required valid days**	- ≥ 3 wk days and ≥ 1 wknd day (4/18) [41, 45, 50, 54] - ≥ 3 days (3/18) [43, 51, 57] N.R. (3/18) [47, 48, 52] 5 days including 2 weekend days (2/18) [53, 56] 1 day (2/18) [42, 58] - ≥ 1 day (1/18) [55] 6 days (1/18) [44] 2 wk days and 1 wknd day (1/18) [46] 2 days (1/18) [49]	- ≥ 3 wk days and ≥ 1 wknd day (7/17) [59, 63, 64, 66, 69, 71, 74] - ≥ 3 days (3/17) [62, 65, 68] N.R. (2/17) [60, 67] - ≥ 2 wk days and 1 wknd day (2/17) [61, 75] - ≥ 5 nights and ≥ 4 days, including 1 weekend night and day (1/17) [70] - ≥ 6 days (1/17) [72] - ≥ 1 day (1/17) [73]	- ≥ 3 wk days and ≥ 1 wknd day (6/23) [77, 78, 80, 84, 89, 92] - ≥ 3 days (5/23) [82, 94–96, 145] N.R. (4/23) [81, 85, 88, 91] - ≥ 4 days (2/23) [79, 86] - ≥ 5 days including ≥ 1 weekend day (1/23) [76] - ≥ 1 day (1/23) [83] - ≥ 6 day (1/23) [87, 100] - ≥ 4 days and ≥ 3 nights (1/23) [90]	- ≥ 3 wk days and ≥ 1 wknd days (9/44) [102, 104, 105, 110, 114, 117, 119, 125, 141] N.R. (8/44) [98, 101, 107–109, 132, 135, 138] - ≥ 4 days (8/44) [111, 113, 116, 121, 130, 131, 133, 134] - ≥ 3 days (5/44) [123, 124, 126, 129] - ≥ 1 day (5/44) [99, 115, 118, 120] - ≥ 3 wk days and 2 wknd days (1/44) [117] - ≥ 7 wk days and ≥ 2 wknd day (2/44) [106, 127] - ≥ 5 day (1/44) [181] - ≥ 8 days (1/44) [140] - ≥ 6 days including 1 wknd day and ≥ 7 nights (1/44) [100] - ≥ 4 days including 3 nights (1/44) [137] - ≥ 5 days, including 2 weekend days (1/44) [136]
**Minutes required to be a valid 24-h period**	24-h (3/18) [41, 42, 58] N.R. (3/18) [43, 47, 53] ≧1,000 min/day (2/18) [45, 54] - ≥ 8 waking hours (2/18) [48, 51] - ≥ 10 waking h/day (2/18) [56, 57] - ≥ 600 min during awake time and an average sleep time ≥ 200 min (1/18) [44] - ≥ 16 h/day (1/18) [46] All sleep h and 8 waking h/day (1/18) [49] - ≥ 7 waking hours for PA and > 160 min for sleep (1/18) [50] - ≥ 480 min of wear time and full night (1/18) [52] - ≥ 6 waking h/day (1/18) [55]	- ≥ 10 waking hours/day (3/17) [59, 64, 65] - ≥ 16 h/day (3/17) [61, 68, 71] 24-h (2/17) [73, 75] - ≥ 10 waking h/day for PA and ≥ 160 min per night and > 90% estimated wear time for sleep (1/17) [59] - ≥ 10 waking hours/day and if the total time was between 20 and 28 h (1/17) [65] N.R. (1/17) [60] - ≥ 20 h/day (1/17) [62] - ≥ 9 waking h/day (1/17) [63] - ≥ 10 waking h/day with their respective sleeping periods (1/17) [66] - ≥ 10 waking h/day and with step estimates between 1000 and 30,000 steps (1/17) [67] - ≥ 10 waking hours and nights were considered valid if the participant provided 20 min of wear time before sleep onset (1/17) [70] - ≥ 600 min/ day in waking time and an average sleep time ≥ 200 min (1/17) [72]	- ≥ 10 waking hours/day (8/23) [76, 80, 81, 86, 92, 94–96] - ≥ 16 h/day (4/23) [77, 78, 82, 84] - ≥ 10 waking h/day or ≥ 200 min/day for sleep or ≥ 1000 min/d for SB (1/23) [79] - ≥ 10 waking h/day; total wear time or wear time plus imputed physical activity ≥ 20 h; ≥ 2 h of sleep (1/23) [145] - ≥ 16 h/day with these hours being roughly equally distributed between the morning (3 am-9 am), noon (9 am-3 pm), afternoon (3 pm-9 pm), and midnight (9 pm-3 am) parts of the day) (1/23) [83] - ≥ 13 waking h/day (1/23) [85] - ≥ 20 h/day (1/23) [87] N.R. (1/23) [88] Adhere to sleep intervention and > 10 h/day waking hours (1/23) 24-h/day (1/23) [89] - ≥ 10 waking h for PA and SB, and ≥ 160 min for sleep (1/23) [90] - ≥ 16 h/day and < 6 h or > 12 h of sleep (1/23) [91] - ≥ 1000 min/day (1/23) [93]	- ≥ 10 waking h/day (17/44) [99, 105, 108, 111, 113–115, 118, 119, 122–124, 126–128, 131, 133, 137, 138, 140, 141] N.R. (5/44) [98, 101, 132, 135, 139] - ≥ 16 h/day (5/44) [103, 104, 107, 110, 125] 24-h/day (3/44) - ≥ 600 min/day (2/44) - ≥ 8 waking h/day (2/44) - ≥ 1296 min (1/44) - ≥ 4 h of work and leisure and ≥ 4 of sleep (1/44) - ≥ 3 h/day (1/44) - ≥ 23 h/day (1/44) - ≥ 21 h/day (1/44) - ≥ 23 h/day (1/44) - ≥ 10 waking h/day and with the sleep period detected (1/44) - > 1200 min/day (1/44) - ≥ 10 and ≥ 8 waking/h day on wk days and wknd days, respectively (1/44) - ≥ 18.5/h day (1/44)
**Criteria for non-wear time**	- ≥ 20 min of consecutive zeros (9/18; 2 studies only on waking hours) [49–51, 53–58] N.R. (2/18) [44, 47] Choi algorithm (2/18) [43, 45] No non-wear time (1/18) [41] Through manually and visually Screened (1/18) [42] Based on the SD and value range of each axis, using a 60 min window with 15 min increments (1/18) [46] If no movement was recorded at night, it was assumed that the acc was removed prior to bedtime 60 consecutive min of nonmovement was defined as an invalid hour. (1/18) [48] Off-wrist detection with a button to mark events in the record (1/18) [52]	-N.R. (3/17) [64, 67, 72] - ≥ 20 min of consecutive zeros (3/17; 1 study only on waking hours) [59, 62, 65] - ≥ 10 min of consecutive zeros (3/17: 1 study only on waking hours) [60, 74, 75] Based on the SD and value range of each axis, using a 60 min window with 15 min increments (2/17) [61, 68] Troiano (2007) algorithm provided by ActiLife (1/17) [63] Tudor-Locke et al. 2015 filter (1/17) [66] - ≥ 15 min of consecutive zeros (1/17) [69] Defined by diary (1/17) [70] R-package GGIR detects non-wear time (1/17) [71] Using the in-built temperature sensor (1/17) [73]	N.R. (4/23) [79–81, 96] Based on the SD and value range of each axis, using a 60 min window with 15 min increments (4/23) [77, 82, 84, 91] - ≥ 20 min of consecutive zeros (3/23) [76, 90, 93] Defined by diary (2/23) [87, 145] - ≥ 60 consecutive minutes of zero counts, with allowance of 2 min of counts between 0 and 100 (2/23) [86, 92] combination of prolonged periods of zero acceleration accompanied by non-physiological heart rate data, and data were adjusted to minimize potential diurnal bias during summarization (1/23) [83] Choi algorithm (1/23) [85] Remotion of the accelerometer (1/23) [88] Detected by body temperature sensor (1/23) [89] Manufacturer’s algorithm and visually verified by condition-blind study staff (1/23) [94] - ≥ 90 min of consecutive zero counts (1/23) [95]	N.R. (17/44) [98, 100, 104, 107, 110, 115, 117, 123–125, 127, 131, 134–136, 140, 141] - ≥ 60 min of consecutive zeros (6/44; 2 study this was supported by diary and visual inspection) [108, 119, 120, 130, 133, 138] Based on the SD and value range of each axis, using a 60 min window with 15 min increments (4/44) [102, 113, 121, 137] Temperature outputs and visual inspection (1/44) [101] Choi’s algorithm 2007 and 2011 (3/44) [111, 118, 129] - ≥ 90 min of consecutive zeros (2/44) [132, 181] Defined by diary (2/44) [128, 139] When heart rate counts were zero (1/44) [97] No non-wear time (1/44) [99] - ≥ 60 consecutive minutes of zero counts, with allowance of 2 min of counts between 0 and 100 (1/44) [108] Periods of 60 min of less than 25 counts per minute (1/44) [114] > 120 min in quiescent time (1/44) [116] 2 h or any bout ≥ 5 h without detectable movement (1/44) [126] Directly measure non-wear time (2/44) [106, 112] Time periods where the SD of acceleration in all 3 axes fell below 13 mg for > 1 h and was excluded from analyses (1/44) [121]
**Epochs Length and data reduction (epochs integration)**	15-s epochs (10/18) [42, 45, 48–53, 55, 58] 1 min epochs (3/18) [41, 47, 54] Sampled at 30 Hz and reintegrated into 5-s epochs (2/18) [56] Sampled at 87.5 Hz and reintegrated into 1-s epochs (1/18) [44, 57] 80 Hz (1/18) [46] Sampled at 80 Hz and reintegrated into 15-s epochs for PA and 1 min epochs for sleep (1/18) [50] 1-s epochs (1/18) [43]	1 min epochs (5/17) [60, 63, 66, 69, 75] Sampled at 30 Hz and converted in 1-s (1/17) [68] and 1 min (2/17) [70, 74] 15-s epochs. (2/17) [62, 65] Sampled at 80 Hz, downloaded in 1-s epochs, and reintegrated to 15and 60-s epochs (1/17) [59] 80 Hz (1/17) [61] N.R. (1/17) [64] 1-s epochs for PA and 1 min epochs for sleep (1/17) [67] Sampled at 87.5 Hz and converted into 5-s by R-package GGIR (1/17) [71] 87.5 Hz (1/17) [72] Sampled at 100 Hz and reintegrated in 5-s epochs (1/17) [73]	N.R. (2/23) [82, 92] Sampled at 30 Hz and reintegrated into 15-s (1/23) [76], 5-s (1/23) [78], and 1 min epochs (1/23) [90] Sampled at 100 Hz and reintegrated into 5-s (2/23) [84, 91] and 1-s epochs (1/23) [89] 1 min epochs. (2/23) [93] Sampled at 75 Hz and reintegrated into 5-s epochs (1/23) [77] Sampled at 50 Hz and reintegrated into 1 min epochs (1/23) [79] Sampled at 30 Hz and the measured accelerations stored at 1 Hz after conversion into proprietary “activity count units” summed over a 1 s epochs For PA: 1 min epochs was used (1/23) [80] Sampled at 30 Hz and reintegrated into 15-s epochs and collapsed into 1 min-epochs (1/23) [145] Sampled at 100 Hz and converted to 1 omnidirectional measure of acceleration (ENMO), that was reintegrated into 5-s epochs (1/23) [82] 30-s epochs (1/23) [83] 5-s epochs (1/23) [85] 15-s epochs (1/23) [86] 20 Hz (1/23) [87] -30-s 1-s epochs for PA; 60-s 1-s epochs for sleep (1/23) [94] Sampled at 80 Hz and reintegrated into 1-s epochs (1/23) [95] 1 min epochs (1/23) [88, 96]	1 min epochs (10/44) [97, 98, 100, 105, 106, 108, 112, 132, 133, 136, 138] N.R. (9/44) [117, 118, 120, 128, 137, 139, 140, 181] Sampled at 100 Hz and reintegrated into 1-s (2/44) [104, 110] , 6-s (1/44) [116], and 1 min epochs (2/44) [109, 111] Sampled at 30 Hz. reintegrated into 5-s (1/44) [135], and 1 min epochs (3/44) [129–131] 20 Hz (3/44) [99, 119, 126] Sampled at 50 Hz, reintegrated into 1 min epochs (2/44) [114, 127] 50 Hz (1/44) [121] 40 Hz and reintegrated into 1 min epochs (1/44) [101] 100 Hz (1/44) [103] Sampled at 30 Hz and reintegrated into 1 min epochs for sleep and into 15-s epochs for PA (1/44) [107] 80 Hz (1/44) [113] 32 Hz (1/44) [115] 40 Hz (1/44) [123] 30-s epochs (1/44) [124] 5-s epochs (1/44) [125] 1-s epochs (1/44) [134] Sampled at 100 Hz and processed as 10-s epoch. Sleep was analyzed by 1 min epoch (1/44) [141]
**Movement Behavior outcome and Reporting formats**	Sleep, SB, LPA and MVPA (h or min /day) (5/18) [43, 46, 49, 52, 53] Sleep, SB, LPA, and MPA (h or min/day) (4/18)[50, 55–57] Sleep, SB, LPA, MPA, VPA and TPA (h or min/day) (2/18) [41, 44] Sleep, SB, TPA (% of 24-h) (1/18) [47] Sleep, SB, LPA, MVPA, TPA (h or min/day) (2/18) [42, 45] Sleep, SB, MPA and VPA (h or min/day) (1/18) [48] Sleep, SB, TPA (min/day) (1/18)[51] Sleep, SB, MVPA (h or min/day) (1/18) [54] Sleep, SB, Screen time, LPA, MPA and VPA (h or min/day) (1/18) [58]	Sleep, SB, LPA, MVPA (h or min/day) (6/17) [60, 64, 65, 68, 70, 73] Sleep, SB, LPA, MPA and VPA (h or min/day) (3/17) [61, 74, 75] Sleep, SB and MVPA (h or min/day) (3/17) [59, 66, 67] Sleep, SB, LPA, MVPA (% of 24-h period) (1/17) [62] Sleep, SB, LPA and MVPA (min day); TPA (cpm); Steps (min^−1^) (1/17) [63] Sleep, SB, LPA. MVPA (h or min day); TPA (cpm) (1/17) [69] Sleep, SB, LPA, MPA, VPA, MVPA (min/day) (1/17) [71] Sleep, SB, LPA, MVPA and TPA (min/day) (1/17) [72]	Sleep, SB, LPA, MVPA (min/day) (13/23) [78, 79, 82, 84–88, 90, 91, 94, 145] Sleep, SB, LPA, MPA, VPA, MVPA (h or min/day) (3/23) [76, 92, 93] Sleep, SB, and MVPA (h or min/day) (2/23) [95, 96] Sleep, SB, LPA, MVPA (min/day and % of 24-h) (1/23) [77] Sleep, SB and LPA (% of day), MVPA (min/day) (1/23) [80] Sleep, SB, LPA, MPA and VPA (h or min/day) (2/23) [83, 88] Sleep, SB and VPA (h or min/day) (1/23) [89]	Sleep, SB, LPA, MVPA (min/day) (20/44) [97, 99, 103, 107, 109, 111, 117, 120, 121, 123, 124, 127, 128, 132, 134–138] Sleep, SB, and MVPA (h or min/day) (3/44)^5^[130, 131, 181] Sleep, SB, LPA, MPA and VPA (h or min/day) (3/44) [125, 126, 133] Sleep, SB, LPA, MPA, VPA and MVPA (h or min/day) (3/44) [100, 112, 141] N.R. (2/44) [101, 110] Sleep, SB, LPA and MVPA (min day and % of 24-h) (3/44) [105, 114, 115] Sleep, SB, LPA, MVPA (proportion of the 24-h day) (1/44) [113] Sleep (h/day) SB and MVPA (time %), and number of steps per day (1/44) [98] Sleep, SB, LPA, MVPA, TPA (h or min/day) (1/44) [104] Sleep and SB (h/day); PA (steps/day) (1/44) [106] Sleep, SB, standing time, LPA, MVPA (% of 24-h) (1/44) [119] Sleep, SB, LPA, MPA, VPA, and VVPA (h or min/day) (1/44) [129] Sleep, SB, and standing (h/day), Stepping (steps/min) (1/44) [139] Sleep, SB, LPA, MPA, VPA, VVPA (% of time) (1/44) [140] Sleep, SB, standing, LPA, MPA, VPA (h or min/day) (1/44) [116] “Minutes asleep” for each sleep episode and “Total minutes asleep” within a day Daily total step counts, “Very active minutes”, “Fairly active minutes”, and “Lightly active minutes” (1/44) [108]
**Sleep measuring procedure**	Manually and visually screened, considering log and accelerometer files (4/18) [42, 49, 50, 55] Sleep algorithm (Hees et al. 2015) (2/18) [44, 46] Sleep algorithm (Sadeh et al. 1994) (2/18) [48, 53] Sleep algorithm (Hjorth et al. 2012) (2/18) [56, 57] number of min asleep between sleep onset and sleep offset.; Daytime napping: 30 successive epochs of sleep that occurred between 09:00 and 17:00 (1/18) [41] Sleep algorithm Tudor-Locke (2014) (1/18) [45] CREA algorithm (1/18) [47] Sleep onset, as the start of the first 15 continuous minutes of sleep preceded by 5 min of awake, to offset, as the last of 15 continuous minutes of sleep followed by 5 min of awake. Matlab script defines naps during daytime wake periods (9 a.m.–5 p.m.) as at least 30 min of continuous sleep, preceded by 5 min of awake) (1/18) [51] Combination of sleep diaries and button-marked events (1/18) [52] Identified by abrupt increases in activity and heart rate and supported by diaries (1/18) [54] Sadeh and Tudor-Locke algorithms (1/18) [43] At least 10 consecutive minutes with a vector magnitude > 0 (1/18) [58]	Sleep algorithm (Sadeh et al. 1994) (4/17) [63, 69, 70, 74] Sleep algorithm (Hees et al. 2015) (3/17) [68, 71, 72] Sleep algorithm (Tudor-Locke et al. 2014) (2/17) [59, 73] Sleep algorithm (Meredith-Jones et al. 2016) (2/17) [62, 65] Sleep algorithm (Hees et al. 2018) (1/17) [61] Sleep period lasting > 240 min with sleep onset between 7 PM and 6 AM and offset between 5 AM and 1 PM (1/17) [64] Sleep algorithm (Barreira et al. 2015) (1/17) [66] Sleep algorithm (Cole–Kripke et al. 1992) (1/17) [60] - > 160 consecutive minutes classified as “asleep” between 7:00 pm and 11:58 am (1/17) [67] The point when the cpm data changed to consecutive zeros lasting about 8–10 h and cross-checked with the participants’ wear log for “off’ times (1/17) [75]	Sleep algorithm (Sadeh et al. 1994) (5/23) [80, 82, 85, 90, 145] Manually and visually screened, considering log and accelerometer files (2/23) [86, 93] Sleep algorithm (Hees et al. 2018) (4/23) [78, 82, 91, 95] Sleep algorithm (Hees et al. 2015) (3/23) [77, 79, 84] Summing activPAL4 output called “primary lying time” and self-reported napping time (1/23) [87] Sleep algorithm (GENEActiv Post-Processing PC Software) with support of body temperature and luminosity sensors (1/23) [89] Sleep algorithm (Barreira et al. 2015) (1/23) [92] CREA algorithm (1/23) [76] Sleep algorithm (Meltzer et al. 2012) (1/23) [94] N.R. (2/23) [88, 96]	Sleep Algorithm (Cole–Kripke et al. 1992) (9/44) [98, 107, 111, 118, 130, 132, 138, 141, 181] Sleep Algorithm (Hees et al. 2015) (6/44) [104, 113, 121, 127, 135, 137] Manually and visually screened, considering log and accelerometer files (5/44) [105, 114, 120, 128, 140] Sleep Algorithm (Hees et al. 2018) (3/44) [103, 123, 125] Sleep Algorithm (Oakley 1997) (3/44) [124, 131, 133] SenseWear Sleep algorithm (3/44) [112, 134, 136] Sleep Algorithm (Shin et al. 2015) (1/44) [117] Sleep Algorithm (Winkler et al. 2016) (2/44) [99, 126] Number of minutes asleep between sleep onset and sleep offset: with no movement for more than 20 min; was measured between 6:00 pm and 5:59 am and corrected visually (1/44) [97] Pressing an event marker button (1/44) [100] N.R. (3/44) [101, 116, 134] Total nighttime sleep derived from minute-by minute sleep epochs (1/44) [106] Defined as having non-nap sleep duration > 3 h. Nap is a sleep episode with a start time between 8 am to 5 pm. Sleep duration was computed by subtracting total nap minutes from “total minutes asleep.” (1/44) [108] Machine learning model Doherty et al. 2018 (1/44) [109] Total amount of time spent in bed minus sleep onset latency (1/44) [110] Sleep Algorithm (Cabanas-Sanchez et al. 2018) (1/44) [115] The last registered non-sedentary epochs of the day, which was followed by a long uninterrupted sedentary period (> 2 h), was identified as sleep (1/44) [119] Sleep Algorithm (Sadeh et al. 1994) (1/44) [129] Prolonged periods (> 2 h) of continuous inactivity during sleeping hours (extracted from the activPAL raw output) (1/44) [139]
**PA and SB cut points**	Pate et al. 2006 (4/18) [48, 49, 58, 142] Hager et al. 2016 (1/18) [41] Trost et al. 2012 (1/18) [42] SB, LPA, MPA and VPA TPA (total recorded counts/wear time) (1/18) [44] SB (0–467 cp15s), LPA (468–2,207 cp15s); MPA (2,208–3,991 cp15s) and VPA (≥ 3,992 cp15s); No authors (1/18)[45] Hildebrand et al. 2014 and 2017 (1/18)[46] SB (If more than 45 s of the 60 s epochs were spent sitting or lying, these epochs were classified as SB); Activity (Yes if there was no SB and no Sleep) (1/18) [47] Janssen et al. 2003 and Pate et al. 2010 (1/18) [50] Adolph et al. 2012 and Trost et al. 2010 (1/18)[51] Ekblom et al. 2012 (1/18) [52] Evenson et al. 2008 (1/18) [53] Butte et al. 2014 (1/18) [54] Freedson et al. 1998 (1/18) [56] Hjorth et al. 2012 (1/18)[57] Trost et al. 2012 and Butte et al. 2014 (1/18) [43]	Evenson et al. 2008 (7/17); one study did not define the cut-point) [59, 62, 63, 65, 66, 73, 74] SB (0%-19.9% HRR), LPA (20%-49.9% HRR) and MVPA (≥ 50% HRR) (1/17) [64] SB (< 1.5 METs or < 332 cpm), LPA (1.5 to 3 METs or 332 to 1004 cpm), MPA (3 to 6 METs or 1004 to 2336 cpm) and VPA (> 6 METs or > 2336 cpm); no author or N.R. (1/17) [60] Hildebrand et al. 2014 and 2017 (3/17) [61, 71, 72] Chandler et al. 2016, Gavarry et al. 1998 (1/17) [67] Hildebrand et al. 2014 (1/17) [68] Trost et al. 2011 (1/17) [69] Not clear (1/17) [70] Puyau et al. 2004 (1/17) [75]	Hildebrand et al. 2014 and 2017 (3/23) [78, 84, 91] Trost et al. 2011 (2/23) [94] Romanzini et al. 2014 (2/23) [76, 90] Hildebrand et al. 2014 (1/23) [77, 82] Philips et al. 2013 (2/23) [79, 89] Sasaki et al. 2011 (1/23) [80] Kozey-Keadle et al. 2011), and Freedson et al. 1998 (1/23) [145] Hurter et al. 2018 and Hildebrand et al. 2014 (1/23) [82] SB (≤ 1.5 METs), LPA (1.5 to 4 METs), MPA (1.5 to 4 METs) and VPA (> 7 METs); no authors or N.R. (1/23) [83] Chandler et al. 2016 (1/23) [85] Payau et al. 2002 (1/23) [86] SB: summing activPAL4 output “sitting time” and “secondary lying time”, while deducting self-reported napping time; LPA: residual time to wear time (LPA = 24 h—Sleep—SB—MVPA—non-wear time); MVPA (100 steps per minute) (1/23) [87] Evenson et al. 2008 (1/23) [92] Puyau et al. 2004 (1/23) [93] Trost et al. 2012 (1/23) [95] Ekblom et al. 2012 (1/23) [96] N.R. (1/23) [88]	Freedson et al. 1998 (6/44)[98, 105, 111, 129, 133, 134] Hildebrand et al. 2014 (4/44)[104, 113, 125, 135] N.R. (3/44) [101, 110, 128] SB (< 1.5 METs), LPA (1.5–3 METs); MPA (3–6 METs); VPA (6–8.9 METs) Very VPA (9 METs) (2/44) [138, 140] Esliger et al. 2011 (2/44) [114, 127] Shin et al. 2015 (1/44) [117] Rowlands et al. 2018 (2/44) [123, 137] Sasaki et al. 2011 and Aguilar-Farias et al. 2014 (2/44) [130, 131] White et al. 2016 (1/44) [121] SB (≤ 1.5 METs), LPA (1.5–3.0 METs), MVPA (≥ 3 METs); algorithms developed by the Silmee™ (1/44) [97] Hamer et al. 2020 (1/44) [99] SB: subtracted the habitual sleep time from the 24-h sedentary time of each participant to calculate the amount of habitual sedentary time when he or she was awake; LPA, MPA and VPA: Heil 2006 (1/44) [100] Hildebrand et al. 2017 (1/44) [101] SB (≤ 1.5 METs), PA (Average daily steps) (1/44) [106] Evenson et al. 2015 (1/44) [107] SB (subtracting the active and light activity minutes from the total valid wear time); Active minutes: sum of the “very active” and “fairly active” minutes) (1/44) [108] Machine learning model Doherty et al. 2018 (1/44) [109] Behaviors were detected by using proprietary (SenseWear) pattern recognition algorithms that are refined over time (1/44) [112] Lying, reclining, passive sitting, active sitting, standing, walking, and other activities; Cut points were not clear. Walking was divided into two categories according to the 2.5-mph cutpoint, which identifies light (< 2.5 mph) and moderate-to-vigorous (≥ 2.5 mph) intensities (1/44) [115] Copeland et al. 2009 and Matthews et al. 2008 (1/44) [118] SB and standing time (calculated using the postural function of the monitor, through the proprietary software (ActiPal). LPA (24-h—(SB + standing time + MVPA); MVPA: Powell et al. 2017 (1/44) [119] Sitting and/or lying and standing and walking slow (SB and LPA; < 100 steps per min) and running, cycling and stair climbing (MVPA; > 100 steps per min) (1/44) [120] Troiano et al. 2008 (1/44) [181] Landry et al. 2015 (1/44) [124] Winkler et al. 2016 algorithm (1/44) [126] Lee et al. 2019 (1/44) [132] Reece et al. 2015 (1/44) [136] SB (Sleep duration was subtracted from total sedentary time to obtain waking hours‟ sedentary time); Stepping and standing (N.R.) (1/44)[139] Vähä-Ypyä et al. 2015 (1/44)[116] Crouter et al. 2015 (1/44) [141]
**Procedures for handling non-compliant participants**	Non-compliant participants were excluded from the analyses. (17/18) [41–45, 47–58] The invalid data were imputed using the mean value of valid data at same time points on other days (1/18) [46]	Non-compliant participants were excluded from the analyses (13/17) [59, 60, 62–64, 66, 67, 69, 71–75] For each 15 min period detected as non-wear time over the valid wearing days, the invalid data were imputed using the mean value of valid data at same time points on other days (2/17) [61, 68] Non-wear time was reallocated to other day-time components (sedentary, LPA, and MVPA) by multiplying the proportions of day-time wear by total daytime in minutes (1/17) [65] If epochs of low activity existed outside of the scoring interval or if non-wear time occurred during the interval, a consensus was reached by the research team (1/17) [70]	Non-compliant participants were excluded from the analyses. (17/23) [76–78, 80, 81, 83, 85, 86, 88–96] Non-wear data were imputed by the average at similar time points on other days of the week (2/23) [82, 84] For each 15 min period detected as non-wear time over the valid wearing days, missing data were imputed using the mean values calculated from valid data at the same time points on other days. Any days with ≤ 200 min of sleep duration or ≤ 1000 min of ST was considered invalid and were excluded from the analysis (1/23) [77] If the device was removed for “sport”, the corresponding period of non-wear was imputed with 50% MVPA, 30% LPA and 20% sedentary time (1/23) [79] Activities that were performed during non-wear time, were also recorded and then manually imputed (manually imputed data accounted for 0.5% of total wear time) (1/23) [145] If non-wear time existed, the data on lecture/leisure movement behaviors were proportionally rescaled to fit a specific time period (1/23) [87]	Non-compliant participants were excluded from the analyses. (40/44) [97–101, 104–108, 110–131, 133, 134, 136–141] Non-wear data or Abnormal high accelerations were imputed by the average at similar time points on other days of the week (1/44) [103] Intent-to-treat using chained equations (R Studio) (1/44) [135] Find all periods of accelerometer data on other days that are during the same time period and have no missing (1/44)^7^ Non-wear time reallocated to other wake components proportionally based on the time they contributed to the total day (1/44) [132]
**Log or diary**	No use (7/18) [43–47, 50, 51] Sleep and non-wear time diary/log (3/18) [42, 54, 57] Parents reported their child's movement behaviors on questionnaires (3/18) [48, 49, 55] Sleep diary (3/18) [52, 53, 56] EMA for sleep (1/18) [41] Movement behaviors and non-wear time diary (1/18) [58]	No use (9/17) [59–62, 64–67, 71] Sleep and non-wear time diary/log (3/17) [63, 70, 75] Sleep diary/log (3/17) [69, 72, 74] Non-wear time diary (1/17) [68] N.R. (1/17) [73]	Sleep and non-wear time diary (9/23) [79–81, 84, 87, 89, 93, 94, 145] No use (7/23) [77, 78, 82, 90–92, 95] Sleep diary/log (2/23) [88, 96] -Movement behaviors diary (2/23) [76, 86] Time bed questionnaire (1/23) [83] Non-wear time diary (1/23) [85]	Sleep diary/log (17/44) [98–100, 107, 116, 121–124, 129–133, 138, 140, 141] No use (7/44) [97, 102, 106, 108, 109, 115, 117] Sleep and non-wear time diary (7/44) [104, 105, 110, 111, 114, 118, 125] Work times, sleep and non-wear diary (3/44) [120, 125, 128] Movement behavior diary/log (4/44) [101, 112, 136, 137] Non-wear time diary (2/44) [119, 139] N.R. (2/44) [126, 134] Sleep questionnaire (1/44) [127]

SD: Standard Deviation; h: hours; min: minutes; s: seconds; SB: sedentary behavior; PA: physical activity; LPA: light physical activity; MPA: moderate physical activity; VPA: vigorous; MVPA: moderate to vigorous physical activity; TPA: total physical activity; cpm: counts per minute; N.R.: not reported; Hz: Hertz; cp15s: counts per 15 s; METs: Metabolic equivalents; ENMO: Euclidean Norm Minus One; HRR: Heart Rate Reserve

##### Infants, Toddlers and Pre-schoolers

This review included 18 reports [41–58] with toddlers and pre-schoolers (aged 2–5 years). There were no reports with infants (0–2 years).

*Accelerometer Brand and Body Placement Site* Eight different accelerometers or combinations (of accelerometers) were used. The most used accelerometer was the Actigraph GT3X (11 reports out of 18; 61.1%) [42, 43, 45, 46, 48–50, 54–57]. Overall, 10 reports placed the accelerometer on the hip (55.6%) [42, 45, 48–51, 53, 56–58], four on the wrist (22.2%) [43, 44, 46, 52] and one on the ankle [41]. The remaining reports (16.7%) placed the accelerometers in two different sites simultaneously (thigh and hip, or hip and chest or thigh and wrist) [47, 54, 55].

*Number of Data Collection and Required Valid Days* The number of data collection days varied substantially between reports (3 to 16 days), with the most frequent being 7 days (50%) [42, 43, 45–47, 50, 51, 54, 57]. Out of the 18 reports, three reports (16.7%) did not report the number of required valid days [47, 48, 52], four (20%) required ≥ 3 weekdays and ≥ 1 weekend day [41, 45, 50, 54], and three (17%) required ≥ 3 days[43, 51, 57], whereas the remaining ones (8; 44.4%) varied substantially. Regarding criteria for the 24-h period to be considered valid, three reports (17%) did not present this information [43, 47, 53], three (17%) considered one 24-h period as a valid day [41, 42, 58], whereas the remaining reports (12; 66.7%) varied substantially.

*Non-wear Time Criteria* Two reports (11.1%) did not describe the criteria for non-wear time[44, 47], and 50% of the reports considered ≥ 20 min of consecutive zero counts as non-wear time[49–51, 53–58].

*Epoch Length and Sampling Rate* Concerning the epoch length, regardless of the sampling rate (ranging from 30 to 87.5 Hz), the most used epoch length was 15 s [42, 45, 48–53, 55, 58] (10; 55.6%). One report (5.6%) used two different epochs depending on which movement behavior was assessed [50]. Nine reports (50.0%) described the sampling rate [42, 44, 46, 49, 50, 52, 55–57].

*Reporting Formats* The most used reporting formats of MovBeh were temporal units such as h/day or min/day (17; 94.4%) [41–46, 48–58]. One study (5.6%) used the percentage of time over 24-h [47].

*Sleep, SB and PA Algorithms and Cut-Points* To assess sleep, nine reports (50%) used different sleep algorithms (Table 3) and four reports (22.2%) manually and visually screened sleep data, considering a log and the accelerometer files. To assess PA and SB, the choice of the cut-points varied considerably, with that from Pate et al. 2006 [142] being the most frequently used 4; 22.2%) (Table 4).

**Table 3:** Algorithms by accelerometer model

Study	Age group	Brief explanation	Accelerometer/body placement
**Non-wear time**			
**Choi et al. 2012 [**164**]**	Toddlers and Preschoolers (2) [43, 45] Adolescents (1) [85] Adults and older adults (2) [118, 129]	At least 90 min of consecutive zero counts in the vertical axis, with an allowance of up to 2 min of interruptions if no activity counts are detected within 30 min of upstream and downstream from that interval	ActiGraph GT3X on hip (1), on hip and wrist (2) ActiGraph GT9X on wrist (1)
**Movement and posture**			
**VANE algorithm (ActivPAL-Software) [**182**]**	Toddlers and Preschoolers (1) [47]	The algorithm uses the thigh location of the activPAL to identify sitting, standing and stepping events. Upright and not-upright separation is identified by using the inclination function of the accelerometer. The dynamic accelerations during stepping caused by the movement of the thigh and foot strike are used to detect stepping events	ActivPAL on thigh (1)
**BodyMedia algorithm [**183**]**	Adults and older adults (1) [112]	Activity parameters are estimated using proprietary algorithms based on combined information of body movement (captured by a triaxial accelerometer) and physiological responses (assessed with heat flux, galvanic skin response, skin temperature, and near-body temperature sensors)	SenseWear Mini Armband on arm (1)
**Sleep**			
**Sadeh et al. 1994 [**143**]**	Toddlers and Preschoolers (4) [41, 43, 48, 53] Children (4) [63, 69, 70, 74] Adolescents (5) [80, 82, 85, 90, 145] Adults and older adults (1) [129]	Classifies sleep into three categories: Sleep onset, sleep efficiency and wake after sleep onset. These categories are used to calculate total sleep time. The algorithm considers parameters as the magnitude and duration of body movements and the Circadian rhythm, to determine the categories. The time when the person goes to bed is defined as the minute with < 10 cpm after four consecutive minutes of ≥ 10 cpm in the evening and the time, when the person is out of bed is defined as the min before four consecutive minutes of ≥ 10 cpm in the morning	ActiGraph GT3X on wrist (5), on hip (4) SleepWatch on wrist (2) Actical on ankle (1), on waist (1) ActiGraph GT9X on wrist (1)
**Hees et al. 2015 [**184**]**	Toddlers and Preschoolers (2) [44, 46] Children (3) [68, 71, 72] Adolescents (2) [79, 84] Adults and older adults (6) [104, 113, 121, 127, 135, 137]	Periods of time within the bedtime and waking times reported in the daily logs during which there was no change larger than 5° in the arm angle over at least 5 min. The algorithm classifies each 5 s epoch as either sleep or wake. Categorizes sleep into three categories: light sleep, deep sleep and REM sleep. These three categories are used to calculate the total duration of sleep in each state and the proportion of time spent in each of these stages. The algorithm considers parameters as the magnitude of body movements, to determine the depth of sleep	GENEActiv on wrist (7) ActiGraph GT3X on wrist (4) ActiGraph GT9X on wrist (3) ActiSleep on wrist (1)
**Cole–Kripke et al. 1992 [**148**]**	Children (1) [60] Adults and older adults (9) [98, 107, 111, 118, 130, 132, 138, 141, 181]	Categorizes each minute of the identified sleep period as either “awake” or “asleep”. Assisted by log. Classifies sleep into two categories: Effective sleep (without considering any periods of wakefulness) and wake after sleep onset. These categories are used to calculate total sleep time, which is the sum of actual sleep and awakening after sleep onset. The algorithm takes into account parameters as movement amplitude, mean activity and periodic limb movements	Actiwatch 4 on wrist (1) Actigraph GT3X on wrist (6), on hip (1) Actigraph GT9X on wrist (2)
**Hees et al. 2018 [**185**]**	Children (1) [61] Adolescents (4) [77, 82, 95, 185] Adults and older adults (3) [103, 123, 125]	Assessing at the distribution of change in Z-angle, which was applied to detect sustained inactivity bouts where the z-angle did not change by more than 5 degrees for at least 5 min. The algorithm classifies each 5 s epoch as either sleep or wake. Categorizes sleep into three categories: light sleep, deep sleep and REM sleep. These three categories are used to calculate the total duration of sleep in each state and the proportion of time spent in each of these stages. The algorithm considers parameters as the magnitude of body movements, to determine the depth of sleep	Actigraph GT3X on wrist (2) GENEActiv on wrist (3) Actigraph GT9X on wrist (2) Actiwatch on wrist (1)
**Tudor-Locke (2014) [**186**]**	Toddlers and Preschoolers (2) [43, 45] Children (2) [59, 73]	Automated algorithm that requires non accelerometer bedand wake-time inputs, relative to a criterion expert visual analysis of minute-by minute waist-worn accelerometer data. The sleep onset is defined by the inclinometer and also by 2 conditions: the first minute with a recorded vector magnitude of < 1000, followed by at least 4 min of a vector magnitude of < 500 and < 10 steps per min; and at least 90 min needed to elapse between bedtime and wake time. The sleep offset is defined by the inclinometer output and then defined as the first minute with either of the 2 following data patterns: a high amount of activity (> 1500 vector magnitude), accompanied by > 20 steps per min and at least 4 min with a vector magnitude of > 0; or at least 10 consecutive minutes with a vector magnitude of > 0	Actigraph GT3X on wrist (1), on hip (2) Axivity AX3 on thigh and lower back (1)
**Sensewear algorithm; Shin et al. 2015 [**187**]**	Adults and older adults (5) [106, 112, 117, 134, 136]	Detects sleep duration trough the characteristic combination of orientation, motion, temperature, and skin conductivity with each sleep stage	SenseWear armband on the upper arm (5)
**Hjorth et al. 2012 [**175**]**	Toddlers and Preschoolers (2) [56, 57]	Nocturnal activity (counts), total sleep time (min), and sleep efficiency (%) are the three measures of sleep. Nocturnal activity is all counts within the reported time span between lights off and lights on, total sleep time is the minutes scored as sleep within the reported time span between lights off and lights on, and sleep efficiency is the percent of sleeping time within the reported time span between lights off and lights on. Could be used to obtain a proxy measure of total sleep time for ranking purposes in epidemiological studies	Actigraph GT3X on hip (2)
**Meredith-Jones et al. 2016 [**188**]**	Children (2) [62, 65]	An automated script developed in MATLAB that removes the appropriate sleep period for each day for each child individually, to avoid sleep being misclassified as sedentary time	Actigraph GT3X on hip (2)
**Barreira et al. 2015 [**189**]**	Children (1) [66] Adolescents (1) [92]	Is a refinement of the Tudor-Locke algorithm. Sleep onset was defined as the first of five consecutive minutes scored as sleep from the Sadeh + inclinometer algorithm (SIA). Sleep offset was defined as the first of 10 consecutive minutes of SIA-scored wake minutes. In addition, sleep period time (SPT) was only identified if > 160 min elapsed between sleep onset and sleep offset. To only focus on nocturnal sleep, sleep onset could only occur between 7:00 p.m. and 5:59 a.m. Second, the definition of sleep offset was refined and identified as the first of 10 or 20 consecutive SIA-scored wake minutes, depending on the time of day (10 min—5:00 a.m. to 11:58 a.m.; 20 min—9:40 p.m. to 4:59 a.m.). Refinements allowed identification of extended episodes of wakefulness separating the SPT into distinct sleep episodes with multiple sleep onsets and offsets	Actigraph GT3X on hip (2)
**Oakley 1997 [**190**]**	Adults and older adults (3) [124, 131, 133]	Sleep duration is estimated by scoring the data during 30-s epochs as “sleep” or “wake”, after manually editing the sleep period using sleep diary data and event and light markers. Each epoch of data from the Actiwatch is assessed as sleep or wake, based on whether or not the activity score exceeds 40 counts. The average sleep duration is computed from nighttime sleep onset (sleep start time; 5 immobile minutes) and morning wakening (sleep end time; 0 immobile minutes), averaging sleep duration	Actiwatch on wrist (3)
**CREA algorithm (ActivPAL algorithm) [**191**]**	Adolescents (1) [87]	Identifies non-upright events lasting at least one hour and then expanding each event to adjacent non-upright events lasting at least one hour (thus allowing for bathroom breaks and other sleep interruptions), resulting in a container of predominantly non-upright events. The longest container is flagged as the single ‘Primary Lying Time’ variable, which is considered a proxy for time in bed. The beginning of ‘Primary Lying Time’ container is identified by the algorithm as ‘Time in Bed Start Time’, which is considered “bedtime”, and the end of the container is identified as ‘Time in Bed End Time’, which is considered “wake time	ActivPAL on thigh (1)
**GENEActiv Post-Processing PC Software[**192**]**	Adolescents (1) [89]	No information found	GENEActiv on wrist (1)
**Cabanas-Sanchez et al. 2018 [**193**]**	Adults and older adults (1) [115]	Sedentary bouts ≥ 300 min are classed as sleep bouts. If no sedentary bouts ≥ 300 min is found, the algorithm consider as sleep the longest sedentary bout per day that lasted ≥ 120 min. The algorithms then iteratively examine surrounding bouts, determining if they were likely to belong to sleep period time. Surrounding bouts are considered as any bout within a 15 min moving window before or after a sleep bout. Bedtime is established as the first register of the first sleep bout, wake time is considered as the first register after the last sleep bout, and the sleep period time is considered as the time (h) between bedtime and wake time	IDEEA pattern-recognition activity monitor on hip (1)
**Winkler et al. 2016 [**194**]**	Adults and older adults (2) [126]	The longest bout in a 24-h day that is ≥ 2 h or any bout ≥ 5 h without detectable movement was classified as non-wear/sleep. The algorithm also examines surrounding bouts and determines whether they are more likely additional sleep/non-wear or waking wear. Bouts are ‘surrounding’ if any portion is within a 15 min window before or after a sleep/ non-wear bout. All bouts in the sleep window were classed as sleep/non-wear when the window contains any of these: a sitting/lying or standing bout that is long (≥ 2 h), or moderately long (≥ 30 min) with very few (≤ 20) steps in between. This repeats until no more sleep/non-wear is found	ActivPAL on thigh (2)

min: minutes; h: hour; cpm: counts per minute

**Table 4:** Cut-points by accelerometer model and age group

Cut point (nº times)	Cut point/ Accelerometer and Body place	Age group			
		Toddlers/Preschoolers	Children	Adolescents	Adults/Older adults
**Evenson et al. 2008 [**144**] (8)**	SB (0–47 cpm), LPA (48–2031 cpm), MPA (2032–2875 cpm), VPA (≥ 2876) / Actical on waist (#1) Vertical (uniaxial) axis cut-points: SB (0–25 cp15s), LPA (26–573 cp15s). MPA (574–1002 cp15s) and VPA (≥ 1003 cp15s) / Actigraph GT3X on hip (#4) SB (0–100 cpm), LPA (101–2295 cpm), MPA (2296–4011 cpm) and VPA (≥ 4012 cpm) / Actigraph GT3X on hip (#1); ActiGraph GMTI on hip (#1) N.R. / Axivity AX3 on thigh and lower back (#1)	1[53]	3[59, 62, 65] 2[63, 66] 1[73]	1[92]	
**Hildebrand et al. 2014 (7) [**146**] and 2017 [**147**]**	SB (ENMO ≤ 35 mg), LPA (ENMO 35–200 mg*)*, MPA (ENMO 200 to 707 mg) and VPA (ENMO ≥ 707 mg*)*; ENMO values were averaged over 5 s epochs / ActiGraph GT3X on wrist (#4) SB (ENMOZ 0–56.3 mg), LPA (ENMOZ 56.3–191.6 mg*)*, MPA (ENMOZ 191.6–695.8 mg) and VPA (ENMOZ > 695.8 mg) / GENEActiv on wrist (#2) SB (0–40 mg (minus sleep)); LPA (41–199 mg); MVPA (≥ 200 mg) / GENEActiv on wrist (#1)	1[46]	1[61] 2[71, 72]	2[78, 84] 1[91]	
**Freedson et al. 1998 [**149**] (7)**	N.R. / ActiGraph GT3X on hip (#1) SB (0–99 cpm), LPA (100–1951 cpm), MPA (1952–5724 cpm), VPA (5725–9498 cpm), and VVPA (> 9499 cpm) / Actigraph GT9X on wrist (#1); ActiGraph GT3X on hip (#3), ActiGraph GT1M on hip (#1); SensewearPro on arm (#1)	1[56]			6[98, 105, 111, 129, 133, 134]
**Hildebrand et al. 2014 [**146**] (6)**	SB/LPA (2 METs) and MVPA (4 METs) / ActiGraph GT9X on wrist (#1) SB (ENMO < 52 mg); LPA (ENMO 52–191 mg); MVPA (ENMO ≥ 192 mg); ENMO values were averaged over 5 s epochs / GENEActiv on wrist (#1); ActiGraph GT3X on wrist (#1); ActiGraph GT9X on wrist (#1) SB (< 30 mg), LPA (30 < 99 mg), MPA (100 < 399 mg), and VPA (> 400 mg) / ActiGraph GT3X on wrist (#2)		1[68]	1[77]	2[104, 135] 2[113, 125]
**Pate et al. 2006 [**142**] (4)**	SB (≤ 199 cp15s), LPA (≥ 200 to 419 cp15s), MPA (≥ 420 to 841 cp15s) and VPA (≥ 842 cp15s cp15s) / Actigraph GT3X + on hip (#2) SB (< 800 cpm), LPA (800–1679 cpm), MPA (1680–3367 cpm) and VPA (≥ 3368 cpm) / ActiGraph GT3X on hip and activPAL4 on thigh (#1) SB (≤ 100 cpm), LPA (101–799 cpm), MPA (800–1679 cpm), and MVPA (≥ 1680 cpm) / ActiGraph GT9X on waist (#1)	2[48, 49] 1[55] 1[58]			
**Trost et al. 2011 [**165**] (3)**	SB (≤ 100 vertical cpm), LPA (101–2295 vertical cpm) and MVPA (≥ 2296 vertical) / ActiGraph GT3X on hip (#4)		1[69]	2[82, 94]	
**Trost et al. 2012 [**195**] (3)**	SB (< 25 cp15s); LPA (25–420 cp15s) and MVPA (> 420 cp15s) / Actigraph GT3X + on hip (#1), on wrist (#1) SB (< 1.5 MET) and MVPA (≥ 3.8 MET) / Actigraph GT3X + on hip (#1)	2[42, 43]		1[95]	
**Ekblom et al. 2012 [**196**] (2)**	SB (≤ 79 cp15s), LPA (80 to 261 cp15s), MVPA (≥ 262 cp15s) / Actiwatch Spectrum on wrist (#1) SB (0–320 cpm), LPA (321–1047 cpm), and MVPA (≥ 1048 cpm) / Actiwatch Spectrum on wrist (#1)	1[52]		1[96]	
**Butte et al. 2014 [**197**] (2)**	SB (< 821 cpm) and MVPA (> 3909) / ActiGraph GT3X on hip and Actiheart on chest (#1) N.R. / ActiGraph GT3X on wrist (#1)	1[54] 1[43]			
**Puyau et al. 2004 [**198**] (2)**	SB (AEE < 0.01 kcal/kg/min), LPA (AEE 0.01 < 0.04 kcal/kg/min), MPA (AEE 0.04 < 0.10 kcal/kg/min), and VPA (> 0.10 kcal/kg/min) / Actical on hip (#1) SB (< 1.5 PAR), LPA (1.5—2.9 PAR), MPA (3.0–6.0 PAR) and VPA (> 6.0) / ActiGraph GT3X on hip (#1)		1[75]	1[93]	
**Romanzini et al. 2014 [**199**] (2)**	SB (0–180 cp15s), LPA (181–756 cp15s), MPA (7571111 cp15s) and VPA (≥ 1112 cp15s) / ActiGraph GT3X on hip (#2)			2[76, 90]	
**Hildebrand et al. 2017 [**147**] (1)**	SB (ENMO < 45 mg); LPA (ENMO ≥ 45 mg and < 100 mg); MVPA (ENMO ≥ 100 mg); ENMO values were averaged over 5 s epochs / ActiGraph GT9X on wrist (#1), on hip (#1)				1[103]
**Esliger et al. 2011 [**192**] (2)**	SB (< 188 cpm), LPA (188 cpm), MPA (403 cpm), VPA (1131 cpm) / GENEActiv on wrist (#1) SB (< 241 g/min), LPA (241–338 g min), and MVPA (> 338 g min) / GENEActiv on wrist (#1)				1[114] 1[127]
**Shin et al. 2015 [**187**] (2)**	SB (≤ 1.5 MET), LPA (1.5—3 MET) and MVPA (> 3 MET) / SenseWear Pro 3 Armband on arm (#2)				2[112, 117]
**White et al. 2016 [**200**] (1)**	SB (< 48 mg), LPA (48 < 154 mg), MPA (154 < 389 mg) and VPA (> 389 mg) / GENEActiv on wrist (#1)				1[121]
**Sasaki et al. 2011 [**201**]and Aguilar-Farias et al. 2014 [**202**] (2)**	SB (0–199 cpm; Sasaki) and MVPA (> 2690 cpm; Aguilar-Farias) / Actigraph GT3X on hip (#2)				2[130, 131]
**Rowlands et al. 2018 [**203**] (2)**	SB (< 40 mg), LPA (48 < 100 mg), and MVPA (≥ 100 mg) / GENEActiv on wrist (#1) SB (< 30.0 mg), LPA (> 30.0–100.6 mg), and MVPA (> 100.6 mg) / Actigraph GT3X on wrist (#1)				1[123] 1[137]
**Hager et al. 2016 [**204**] (1)**	SB (0–40 cpm), LPA (41 2200 cpm), MVPA (≥ 2201 cpm) / Actical on ankle (#1)	1[41]			
**Janssen et al. 2003 [**205**] and Pate et al. 2010 [**206**] (1)**	SB (≤ 25 cp15s), LPA (> 25 cp15s) and MPA (> 420 cp15s) (Janssen); VPA (> 842 cp15s) (Pate) / Actigraph GT3X on hip (#1)	1[50]			
**Adolph et al. 2012 [**207**]and Trost et al. 2010[**195**] (1)**	SB (0–6 counts cp15s), LPA (7–286 cp15s), MVPA (≥ 287 cp15s) / Actical on waist (#1)	1[51]			
**Hjorth et al. 2012 [**175**] (1)**	SB (≤ 25 cp15s), LPA (26–419 cp15s) and MVPA (≥ 420 cp15s) / ActiGraph GT3X on hip (#1)	1[57]			
**Chandler et al. 2016 [**208**], Gavarry et al. 1998 [**209**] (1)**	SB (0%-19.9% HRR) and MVPA (≥ 50% HRR) / Fitbit Charge 2 on wrist (#1)		1[67]		
**Philips et al. 2013 [**210**] (1)**	SB (244 g.min), LPA (878 g.min) and MVPA (2175 g.min) / GENEActiv accelerometer on wrist (#1)			1[79]	
**Sasaki et al. 2011 [**201**] (1)**	SB (< 200 cpm), LPA (200 to < 3028 cpm), MPA (≥ 3028 and < 4448 cpm), VPA (≥ 4448) / ActiGraph GT3X on hip (#1)			1[80]	
**Kozey-Keadle et al. 2011 [**211**] and Freedson et al.,1998 [**149**] (1)**	SB (< 150 cpm; Kozey-Keadle), LPA (150 to 1951 cpm) and MVPA (≥ 1952 cpm; Freedson) / ActiGraph GT3X on hip (#1)			1[145]	
**Crouter et al. 2015 [**212**] (1)**	SB (0–275 cp5s), LPA (276–415 cp5s), MPA (≥ 778 cp5s) and VPA (≥ 416 cp5s) / ActiGraph GT3X on wrist (#1)				1[141]
**Hurter et al. 2018 [**213**] and Hildebrand et al. 2014 [**146**] (1)**	SB/LPA (ENMO 50 mg; Hurter) and MVPA (ENMO 200 mg; Hildebrand) / ActiGraph GT9X on wrist (#1)			1[82]	
**Chandler et al. 2016 [**208**] (1)**	SB (< 305 cp15s), LPA (306–817 cp15s), MPA (818–1968 cp15s), VPA (≥ 1969 cp15s) / ActiGraph GT9X on wrist (#1)			1[85]	
**Payau et al. 2002 [**214**] (1)**	SB (< 100 cpm), LPA (100–1499 cpm) and MVPA (> 1499 cpm) / Actical on hip (#1)			1[86]	
**Phillips et al. 2012 [**210**] (1)**	SB (< 7 g), VPA (> 60 g) / GENEActiv, on wrist (#1)			1[89]	
**Hamer et al. 2020 [**215**] (1)**	SB (0 steps), LPA (< 100 steps/min), MVPA ≥ 100 steps/min / ActivPAL3 on thigh (#1)				1[99]
**Heil, 2006 [**216**] (1)**	LPA (< 3.0 METs), MPA (≥ 3 to < 6.0 METs), VPA (≥ 6.0 METs) / Actical on waist (#1)				1[100]
**Evenson et al. 2015 [**217**] (1)**	SB (< 19 cp15s); LPA (19–518 cp15s) and MVPA (≥ 519 cp15s) / Actigraph GT3X on hip (#1)				1[107]
**Vähä-Ypyä et al. 2015 [**218**] (1)**	SB and standing (MAD < 22.5) (LPA (≥ 1.5 and < 3.0 (MAD = 22.5 and 91.5 mg)); MVPA (≥ 3.0 (MAD > 91.5 mg)) / UKK RM42 on hip (#1)				1[116]
**Copeland et al. 2009 [**219**] and Matthews et al. 2008[**220**] (1)**	SB (< 100 cpm), MVPA (> 1040 cpm) / ActiGraph GT3X on hip (#1)				1[118]
**Powell et al. 2017 [**221**] (1)**	MVPA (≥ 5123 cp15) / GENEActiv on wrist (#1)				1[119]
**Skotte et al. 2014 [**222**] (1)**	SB and LPA (< 100 steps per min) and MVPA (> 100 steps per min) / ActiGraph GT3X on thigh (#1)				1[120]
**Troiano et al. 2008 [**223**] (1)**	SB (0–99 cpm), MVPA (≥ 2020 cpm) / Actigraph GT3X on hip (#1)				1[181]
**Landry et al. 2015 [**224**] (1)**	SB (< 178.5 cpm), LPA (178.6–562.4 cpm), and MVPA (> 562.4 cpm) / Actiwatch Spectrum on wrist (#1)				1[124]
**Winkler et al. 2016 [**194**] (1)**	Algorithm / ActivPAL on thigh (#1)				1[126]
**Lee et al. 2019 [**225**] (1)**	SB (< 231 cpm), LPA (232–4514 cpm), and MVPA (> 4515 cpm) / Actigraph GT3X on wrist (#1)				1[132]
**Reece et al. 2015 [**226**] (1)**	SB (1.0–1.5 METs); LPA (1.6–2.9 METs) MVPA (≥ 3.0 METS) / SenseWear armband on arm (#1)				1[136]

SB: sedentary behavior; PA: physical activity; LPA: light physical activity; MPA: moderate physical activity; VPA: vigorous; MVPA: moderate to vigorous physical activity; TPA: total physical activity; cpm: counts per minute; N.R.: not reported; cp15s: counts per 15 s; METs: Metabolic equivalents; ENMO: Euclidean Norm Minus One

*Handling Non-compliant Participants Procedures* The most used procedure for handling non-compliant participants was the exclusion of the non-compliant participants from the analysis (17; 94.4%) [41–45, 47–58].

*Use of Logs or Diaries* Seven reports (38.9%) did not use logs or diaries [43–47, 50, 51] whereas the remaining (11; 61.1%) used different instruments to complement accelerometer data.

##### Children

This review included 17 [59–75] reports with children aged 5–10 years.

*Accelerometer Brand and Body Placement Site* Eight different accelerometers or combinations (of accelerometers) were used. The most used accelerometer was the Actigraph GT3X (8; 47.1%) [59, 61–63, 65, 66, 69, 70]. Overall, nine reports placed the accelerometer on the wrist (52.9%) [60, 61, 64, 67, 68, 70–72, 74] and seven on the hip (41.2%) [59, 62, 63, 65, 69, 74, 75]. The remaining reports (11.8%) placed the accelerometers in two different sites simultaneously: one on the thigh and lower back [73], one on the hip and wrist [74].

*Number of Data Collection and Required Valid Days* The number of data collection days varied substantially between reports (3 days to 6 weeks), with the most frequent being 7 days (64.7%) [59–61, 63, 65, 66, 68–70, 73, 74]. Two reports (11.8%) [60, 67] did not present the number of required valid days, seven (41.2%) [59, 63, 64, 66, 69, 71, 74] required ≥ 3 weekdays and ≥ 1 weekend day, and three reports (17.7%) [62, 65, 68] required ≥ 3 days, whereas the remaining varied substantially (5; 29.4%) [61, 70, 72, 73, 75]. Regarding criteria for the 24-h period to be considered valid, one report [60] did not present this information, three (17%) [59, 64, 65] defined that the accelerometer should be used during, at least 10 waking hours, another three reports (17.7%) [61, 68, 71] defined the criteria of 16 h/day, whereas the remaining varied substantially (10; 58.8%) [62, 63, 66, 67, 69, 70, 72–75].

*Non-wear Time Criteria* Three reports (23.5%) [64, 67, 72] did not describe the criteria for non-wear time, three reports (17.7%) [59, 62, 65] used ≥ 20 min of consecutive zero counts, whereas the remaining varied substantially (11; 58.8%) [60, 61, 63, 66, 68–71, 73–75].

*Epoch Length and Sampling Rate* Concerning the epoch length, regardless of the sampling rate (ranging from 30 to 100 Hz), the most used epoch length was 1 min (9; 52.9%) [59, 60, 63, 66, 67, 69, 70, 74, 75]. One report (5.9%) [67] used two different epochs according to the behaviors (8.7%). Eight reports (47.1%) [59, 61, 68, 70–74] presented the sampling rate.

*Reporting Formats* The most used reporting formats of MovBeh were temporal units such as h/day and min/day (16; 94.1%) [59–61, 63–75]. One report (5.9%) [62] used the percentage of time over 24 h.

*Sleep, SB and PA Algorithms and Cut-Points* To assess sleep, this review found 11 different options, with the most used one being that of Sadeh et al. 1994 [143] sleep algorithm, (4; 23.5%) [63, 69, 70, 74]. Fourteen reports (82.4%) [59–63, 65, 66, 68–74] used sleep algorithms (Table 3). To assess PA and SB, the cut-points choice varied considerably, with the Evenson et al. 2008 [144] one being the most frequently used (7; 41.2%) [59, 62, 63, 65, 66, 73, 74] (Table 4).

*Handling Non-compliant Participants Procedures* The most used procedure for handling non-compliant participants was the exclusion of the non-compliant participants from the analysis (13; 76.5%) [59, 60, 62–64, 66, 67, 69, 71–75].

*Use of Logs or Diaries* Nine reports (52.9%) [59–62, 64–67, 71] did not use logs or diaries and the remaining reports (8; 47.1%) [63, 68–70, 72–75] used different instruments to complement the accelerometer data.

##### Adolescents

This review included 22 reports [76–96, 145] with adolescents aged 10 to 17 years.

*Accelerometer Brand and Body Placement Site* Twelve different accelerometers or combinations of accelerometers were used. The most used accelerometer was the Actigraph GT3X (9; 40.9%) [76, 78, 80, 81, 90, 92, 94, 95, 145]. Overall, 10 reports placed the accelerometer on the wrist (45.5%) [77–79, 82, 84, 85, 88, 89, 91, 96], six on the hip (27.3%) [76, 86, 90, 92, 93, 145], one on the chest (4.6%) [83] and one on the thigh (4.6%) [87]. The remaining reports (4; 18.2%) placed the accelerometers on the hip and wrist [80, 81, 94, 95], simultaneously.

*Number of Data Collection and Required Valid Days* The number of data collection days varied substantially between reports (3 to 14 days), with the most frequent one being 7 days (52.2%) [78, 80, 82, 84, 86, 88, 91, 92, 94–96, 145]. Four reports (18.2%) [81, 85, 88, 91] did not present the number of required valid days, six reports (27.3%) [77, 78, 80, 84, 89, 92] required ≥ 3 weekdays and ≥ 1 weekend day, and five reports (22.7%) [82, 94–96, 145] required ≥ 3 days, whereas the remaining varied substantially (7; 31.8%). Regarding criteria for the 24-h period to be considered valid, eight reports (36.4%) [76, 80, 81, 86, 92, 94–96] used the 10 waking hours/day criteria, four reports (18.2%) [77, 78, 82, 84] defined the criteria as 16 h/day, whereas the remaining varied substantially (10; 45.5%).

*Non-wear Time Criteria* Four reports (18.2%) [79–81, 96] did not present the criteria for non-wear time, four reports (18.2%) [77, 82, 84, 91] based the criteria on the standard deviation and value range of each axis, using a 60 min window with 15 min increments, and three reports (13.6%) [76, 90, 93] used the criteria of ≥ 20 min of consecutive zero counts, whereas the remaining varied substantially (11; 50.0%).

*Epoch Length and Sampling Rate* Concerning the epoch length, regardless of the sampling rate (ranging from 30 to 100 Hz), the most used epoch length was 1 min (8; 36.4%) [79, 80, 88, 90, 93, 94, 96, 145]. Two reports used two different epochs according to the behaviors (9.1%) [80, 94]. Thirteen reports (59.1%) [76–80, 82, 84, 87, 89–91, 95, 145] presented the sampling rate.

*Reporting Formats* The most used reporting formats of MovBeh were temporal units, such as hours/day and minutes/day (20; 95.5%) [76, 78, 79, 81–96, 145]. Two reports (9.1%) [77, 80] also used the percentage of 24 h.

*Sleep, SB and PA Algorithms and Cut-Points* To assess sleep, this review found 10 different options, with the most used one being the Sadeh et al. 1994 [143] sleep algorithm (5; 22.7%) [80, 81, 85, 90, 145]. Fifteen reports (68.2%) [77–82, 84, 85, 89–92, 94, 95, 145] used sleep algorithms (Table 3). To assess PA and SB, the cut-points choice varied considerably, with those from Hildebrand et al. 2014 [146] and 2017 [147] being the most frequently used ones (*n* = 3; 13.6%) [78, 84, 91] (Table 4).

*Handling Non-compliant Participants Procedures* The most used procedure for handling non-compliant participants was the exclusion of the non-compliant participants from the analysis (17; 77.3%) [76–78, 80, 81, 83, 85, 86, 88–96].

*Use of Logs or Diaries* Nine reports (40.9%) [79–81, 84, 87, 89, 93, 94, 145] used logs or diaries for sleep assessment and non-wear time, whereas seven reports did not use logs nor diaries [77, 78, 82, 90–92, 95], and the remaining used different instruments to complement the accelerometer data (6; 27.3%) [76, 83, 85, 86, 88, 96].

##### Adults and Older Adults

This review included 44 reports [97–141] for adults and older adults (aged ≥ 18 years).

*Accelerometer Brand and Body Placement Site* Twenty different accelerometers or combinations of accelerometers were used. The most used accelerometer was the Actigraph GT3X (10; 22.7%) [104, 105, 107, 110, 120, 125, 128, 132, 137, 141]. In 20 reports the accelerometer was placed on the wrist (45.5%) [97, 98, 101, 103, 104, 108–110, 113, 114, 121, 123–125, 127, 132, 135, 137, 141], five on the arm (11.4%) [106, 111, 117, 134, 136], six on the thigh (13.6%) [99, 119, 120, 126, 128, 139], and two on the hip (4.6%) [105, 107]. The remaining reports placed the accelerometers in two different sites simultaneously (12; 27.3%) [100, 111, 115, 116, 118, 122, 129–131, 133, 138, 140].

*Number of Data Collection and Required Valid Days* The number of data collection days varied substantially between reports (1 to 25 days), with the most frequent one being seven days (28; 63.6%) [97, 99, 103–105, 109–111, 113, 114, 116–119, 121–126, 129, 131, 133–135, 138, 139, 141]. Eight reports (18.2%) [98, 101, 107–109, 132, 135, 138] did not present the number of required valid days, 9 reports (20.5%) [102, 104, 105, 110, 114, 117, 119, 125, 141] required ≥ 3 weekdays and ≥ 1 weekend day, eight reports (18.2%) [111, 113, 116, 121, 130, 131, 133, 134] required ≥ 4 days, whereas the remaining varied substantially (19; 43.2%). Regarding criteria for the 24-h period to be considered valid, 21 reports (47.7%) [99, 105, 108, 111, 113–115, 118, 119, 122–124, 126–128, 131, 133, 137, 138, 140, 141] defined that the accelerometer should be used during 10 waking hours and another five reports (11.4%) [103, 104, 107, 110, 125] defined the criteria of 16 h/day, whereas the remaining varied substantially (19 out of 44; 43.2%) [97, 98, 100–102, 106, 109, 112, 116, 117, 120, 121, 129, 130, 132, 134–136, 139]. Five reports (11.4%) [98, 101, 132, 135, 139] did not present the required minutes for a day to be considered valid.

*Non-wear Time Criteria* Seventeen reports (38.6%) [98, 100, 104, 107, 110, 115, 117, 123–125, 127, 131, 134–136, 140, 141] did not present the criteria for non-wear time, four reports (9.1%) [102, 113, 121, 137] based the criteria on the standard deviation and value range of each axis, using a 60 min window with 15 min increments, and another six reports (13.6%) [108, 119, 120, 130, 133, 138] used the criteria of ≥ 60 min of consecutive zero counts, whereas the remaining varied substantially (17; 38.6%).

*Epoch Length and Sampling Rate* Concerning the epoch length, regardless of the sampling rate (ranging from 30 to 100 Hz), the most used epoch length was 1 min (20; 45.5%) [97, 98, 100, 105–109, 111, 112, 114, 127, 129–133, 136, 138, 141]. Two reports used two different epochs according to the behaviors (4.6%) [122, 141]. Twenty-two reports (50%) [99, 101, 103, 104, 107, 109–111, 113–116, 119, 121, 123, 126, 127, 129–131, 135, 141] presented the sampling rate, and eight reports (18.2%) [117, 118, 120, 122, 128, 137, 139, 140] did not present the epoch, nor the sampling rate.

*Reporting Formats* The most used reporting formats of MovBeh were temporal units such as hours/day and minutes/day (32; 72.7%) [97, 99, 100, 103, 104, 107, 109, 111, 112, 116–118, 120–138, 141]. The remaining reports differed substantially (13; 29.6%) [98, 101, 103, 105, 106, 108, 110, 113–116, 119, 139, 140], with six using more than one reporting format [98, 105, 106, 114, 115, 139] (e.g., percentage of time and steps).

*Sleep, SB and PA Algorithms and Cut-Points* To assess sleep, this review found 19 different options, with the most used one being that from Cole–Kripke et al. 1992 [148] sleep algorithm, (9; 20.5%) [98, 107, 111, 118, 122, 130, 132, 138, 141]. Thirty reports (68.2%) [98, 99, 103, 104, 107, 109, 111–113, 115, 117, 118, 121–127, 129–138, 141] used sleep algorithms (Table 3). To assess PA and SB, the cut-points choice varied considerably, with the Freedson et al. 1998 [149] cut-point being the most frequently used (6; 13.6%) [98, 105, 111, 129, 133, 134] (Table 4).

*Handling Non-compliant Participants Procedures* The most used procedure for handling non-compliant participants was the exclusion of the non-compliant participants from the analysis (40; 90.9%) [97–101, 104–108, 110–131, 133, 134, 136–141].

*Use of Logs or Diaries* Seventeen reports (38.6%) [98–100, 107, 116, 121–124, 129–133, 138, 140, 141] used logs or diaries for sleep, seven reports (15.9%) [104, 105, 110, 111, 114, 118, 125] used logs and diaries for sleep and non-wear time, other seven reports (15.9%) [97, 102, 106, 108, 109, 115, 117] did not use logs nor diaries, and the remaining used different instruments to complement the accelerometer data (13; 29.6%) [101, 112, 113, 119, 120, 125–128, 134–136, 139].

**Fig. 2:**
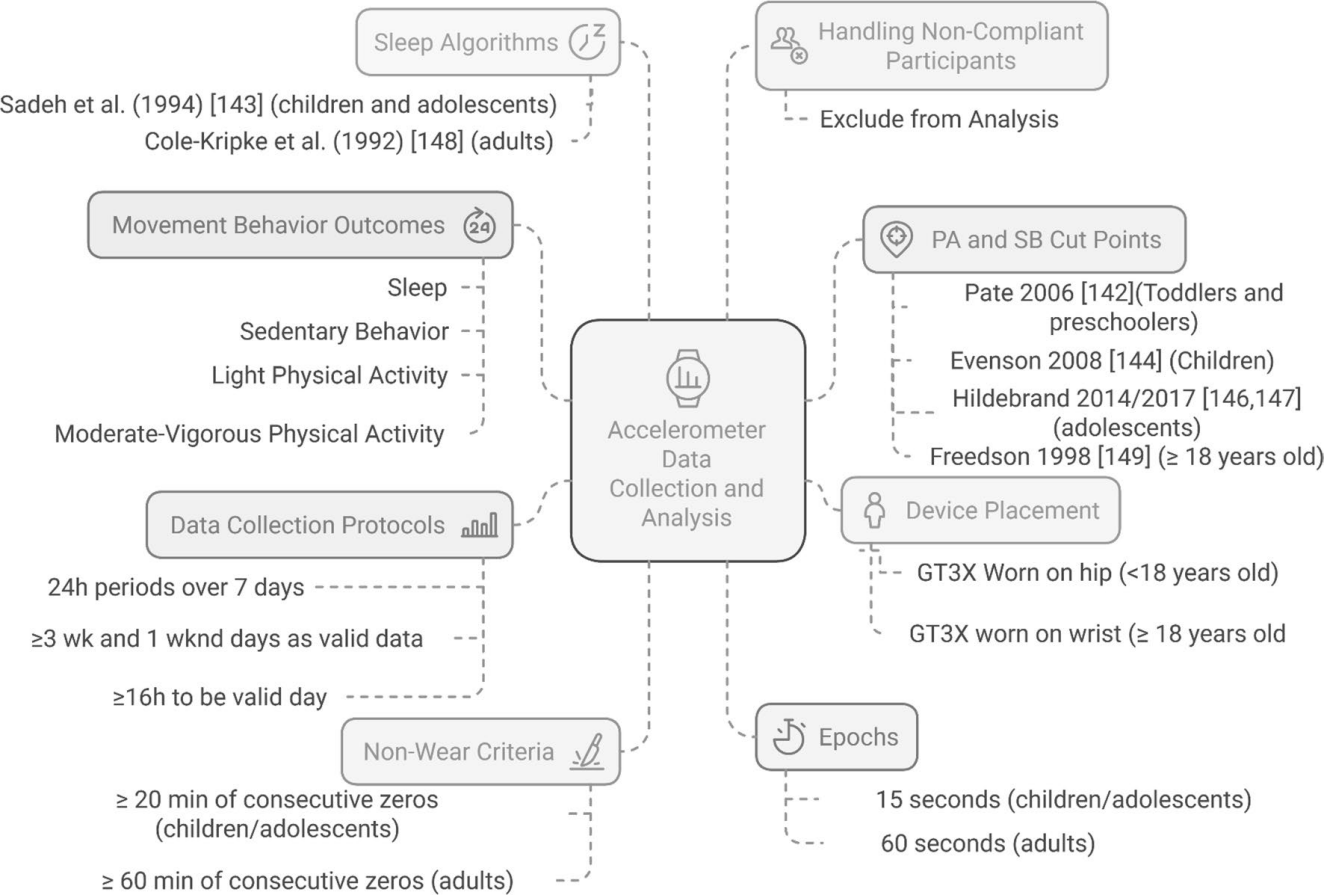
Main results, min: minutes; wk: week; wknd: weekend; PA: Physical Activity; SB: Sedentary Behavior

A summary of most commonly used methods and procedures in studies measuring 24-h MovBeh with accelerometry, across all-age groups are presented in Table 5 and Fig. 2.

**Table 5:** Most commonly used methods and procedures in studies measuring 24-h MovBeh with accelerometry

Accelerometer model and placement on the body	The Actigraph GT3X worn on the hip in toddlers, children and adolescents (< 18 years); The Actigraph GT3X worn on the wrist in adults and older people (≥ 18 years) Some researchers choose to have participants wear one accelerometer on the wrist and another on the hip simultaneously
**Number of days for data collection and number of required valid days; Hours required for a valid day**	24-h periods over seven days; at least three weekdays and one weekend day as valid data; 16 h/day per 24-h period
**Criteria for non-wear time**	≥ 20 min of consecutive zero counts for studies with toddlers, children and adolescents; • ≥ 60 min of consecutive zero counts for adults
**Epochs**	• ≤ 15 s for toddlers, children, and adolescents 60 s for adults
**Movement behavior outcomes and reporting formats**	Sleep, SB, LPA and MVPA; Minutes/hours per day/week
**Sleep algorithms**	The Sadeh et al. (1994) [143] for children and adolescents Cole-Kripke et al. (1992) [148] for adults
**PA and SB cut points**	Pate et al. 2006 [142] for toddlers and preschoolers; Evenson et al. 2008 [144] for children; Hildebrand et al. 2014 [146] and 2017 [147] for adolescents; Freedson et al. 1998 [149] for adults and older adults
**Procedures handling non-compliant participants**	Exclude non-compliant participants from the analysis
**Use of diaries or logs**	Most of the included studies supplemented accelerometer data with diaries or logs

PA: Physical Activity; SB: Sedentary Behavior

### Discussion

This scoping review summarizes the most commonly used methodologies for assessing 24-h MovBeh using accelerometry across the lifespan and included 102 studies. There was considerable heterogeneity between the reports, demonstrating the wide range of options available in terms of the choice of accelerometry to assess 24-h MovBeh and the lack of consensus between researchers.

This review found that the most commonly used accelerometer was the Actigraph GT3X, placed on the wrist in adults and older adults, and on the hip in other age groups. The choice of an appropriate accelerometer and body site depends mainly on the type of MovBeh being measured, as different accelerometers and body sites have different strengths and weaknesses. For example, wrist-worn accelerometers seem to be more accurate in assessing sleep quality metrics, whereas the hip-worn accelerometer may be better for sleep timing and quantity metrics [150]. Hip-worn accelerometers have also been shown to be the best option for PA behaviors (mainly MVPA) and thigh-worn accelerometers (e.g. ActivPal) for postural behaviors (e.g. SB) [151], although the devices used in each of these locations may provide different metrics for each of the behaviors [19, 152]. The choice of accelerometer wearing position is crucial and varies according to age group and the context of application. While the wearing position may seem trivial, it has significant implications due to the variability in measurement results and the specific needs of different populations. For instance, in young children who crawl or roll, a sensor on the thigh can be impractical and may negatively impact data accuracy, whereas adolescents may face compliance challenges when wearing devices that do not align with their aesthetic preferences, such as an ActiGraph worn on the hip. In low- and middle-income countries, wearing a device on the thigh may challenge cultural and aesthetic barriers, making wrist-worn devices more acceptable and easier to implement. Additionally, the cost of devices is also a relevant factor, as advanced sensors can be very expensive, while simpler and more affordable accelerometers, while promising, often lack validation for various wearing positions. This variability in the choice of position and device type requires a careful and context-specific approach to ensure data accuracy and participant’s compliance. In terms of measurement properties, and despite the lack of consensus [27], both the hip- and wrist-worn accelerometers appear to be suitable to classify PA intensities in toddlers and preschoolers [153, 154]. In adults, some data suggest that wrist-worn accelerometers have better measurement characteristics, particularly for household activities, whereas hip-worn accelerometers are more suitable for locomotion [155, 156].

Ideally, evidence should focus on understanding the best accelerometer brand/model and body placement that can capture sleep, SB and PA in an integrated way, without having to change body placement. However, there are currently no valid algorithms to do this, and hip and wrist placements provide quantitatively different information about sleep and PA. Therefore, new algorithms and the potential integration of supplementary sensors (e.g. inclinometers, galvanic skin response or heart rate monitors) are required. This is because, these new features could assist in accurately quantifying and differenting between sleep duration and PA, as well as the non-wear time.

Regarding the number of days of data collection, the most common number of days across all age groups in the studies included in this review was 7 days. MovBeh are inherently variable because people do not exhibit the same behaviors every day, especially children and adolescents; therefore, such a decision depends on the main objective of the study and should reflect a cost–benefit balance between data validity and reliability and participant burden. For example, short data collection periods (e.g. 1–3 days) may be useful if the aim is to measure MovBeh in a specific context, such as a working day or a physical education class. On the other hand, long-term data collection (e.g. at least 7 days) may be useful to accurately measure MovBeh in everyday life [157, 158], increasing the likelihood of obtaining a sufficient number of valid days and the chance of obtaining both work/week and non-work/weekend days [27, 34, 157].

The most common minimum number of valid days required in the included reports was 3 weekdays and one weekend day, which is in line with previous recommendations [27] This recommendation emphasises the importance of capturing both work/weekday and non-work/weekend days. This recommendation emphasises the importance of including both work/weekday and non-work/weekend days. This is critical given the typical differences in patterns between these types of days [159, 160].

The number of minutes required for a valid day varied considerably between the included reports. A recording period of 24 h is recommended, as this increases both the recording time and the wear time compliance [161]; similarly, the criteria for a valid day should be specific to both waking and sleeping hours. In fact, some of the included reports only set a minimum wear time during waking hours. Regarding what defines a day, different methods for defining a 24-h day in accelerometry-based MovBeh studies can impact data collection and interpretation. Common approaches include the midnight-to-midnight method, which aligns with the standard calendar day and is practical for synchronizing with other datasets but may not accurately represent individual sleep–wake cycles. The wake-to-wake method, which spans from one waking time to the next, provides a more individualized assessment aligned with personal activity patterns, though it can introduce complexity in data processing, especially with irregular sleep schedules. The sleep-to-sleep method focuses on the period between sleep onsets, which can be useful for examining sleep's impact on daily behaviors but requires careful handling of naps and varied sleep timing. The choice of method should align with the study’s objectives and balance practical considerations with the need for personalized data insight; however, this information is not described in the protocols of the studies included in our review.

In this review, the most common non-wear time criteria were ≥ 20 min of consecutive zero counts; and based on the standard deviation and range of values for each axis, using a 60 min window with 15 min increments. Given that participants occasionally remove their accelerometers, it is necessary to account for non-wear time, either through self-report or non-wear time algorithms. Indeed, manual input of non-wear time with self-report data is time consuming making the analysis of accelerometry more subjective. On the other hand, the use of non-wear time algorithms is less time consuming and more objective [24]. However, there are different algorithms for different age groups and further studies are needed to evaluate them [27]. In children and adolescents, the most used criterion is ≥ 20 min of consecutive zero counts to flag as non-wear time. In adults, it seems that the criterion of non-wear time of ≥ 20 min or ≥ 60 min of consecutive zero counts is the most commonly used and sufficiently accurate to detect non-wear time [162]. In older adults, Choi’s algorithm is the most commonly used and the most recommended by previous evidence [163]. The discussion on non-wear time should also consider the limitations inherent in the various algorithms used to detect non-wear periods. These algorithms, often based on specific thresholds of consecutive minutes of zero acceleration, can misclassify non-wear time as sedentary behavior, particularly in populations with low movement patterns, such as older adults or individuals with mobility limitations [27, 164]. Additionally, algorithms that rely on fixed time thresholds may not accurately capture non-wear periods across diverse age groups or activity levels. For instance, while a 60 min threshold might be appropriate for detecting non-wear time in adults, it may not effectively capture periods of non-wear in children, who might exhibit more frequent short bursts of activity followed by rest [165]. Furthermore, advanced machine learning-based algorithms, though potentially more accurate, require substantial data processing power and may not be feasible for all research settings due to cost or expertise limitations [166]. These nuances highlight the importance of selecting non-wear time algorithms that align with the target population and study objectives to ensure data accuracy and consistency.

In this review, sampling rates ranged from 30 to 100 Hz, although some studies did not report this information. For the assessment of MovBeh, a sampling rate of 30 Hz is considered adequate [24], because sampling rates in multiples of 30 produce the most accurate estimates, whereas other sampling rates (i.e. not in multiples of 30) could result in an increase of activity counts [167]. Therefore, the current recommendation is to use the highest possible sampling rate [27].

This review found that the most commonly used epoch was 15 s for toddlers, and 60 s epoch for all other age groups; however, there was a range of different epochs were used, possibly because there is no internationally accepted consensus for this matter [24]. In fact, given the spontaneous and intermittent nature of children's MovBeh [168], epochs of 1–15 s are recommended [27, 169] to accurately capture the childrens’ MovBeh. In adults, the most commonly used epoch length was 60 s, but shorter epochs are recommended as they provide more accurate estimates [169]. For sleep, a 60 s epoch is recommended after the most common sleep algorithms were validated using this epoch length, probably because of the stable movement pattern during sleep [143, 148, 170]. The choice of epoch length can drastically alter the final result [34, 171] and shorter epochs can be reintegrated into larger epochs. It is therefore important to develop a consensus on these issues in order to compare studies.

There was considerable variation in the methods used to assess sleep duration. In toddlers and preschoolers, the most commonly used method was manual and visual screening, using logs and accelerometer files. Some reported the use of sleep algorithms. In children and adolescents, the most commonly used sleep algorithm was the Sadeh et al. 1994 sleep algorithm [143]. In adults and older adults, the most commonly used algorithm was the Cole-Kripke et al. 1992 sleep algorithm [60]. All of these algorithms are consistent with previous findings [34]. However, it is well known that sleep measurements from different accelerometers are not comparable, even if the same sleep algorithms are used [172].

Previous studies have reported that wrist-worn accelerometers are better suited for measuring sleep activity, as compared with other body placement locations, because of the existence of algorithms [173–175]. However, the use of accelerometers on the wrist should not be justified solely on the basis of the existence of algorithms, as visual analysis and manual scoring of sleep behavior, with the assistance of data from diaries or logs, can be equally accurate. The reports included in this review used different cut-points to assess PA and SB. The most commonly used cut-points were Pate et al. 2006 [142] for toddlers and preschoolers, Evenson et al. 2008 [144] for children, Hildebrand et al. 2014 [146] and Hildebrand et al. 2017 [147] and Trost et al. 2011 [165] for adolescents, and Freedson et al. 1998 [149] for adults and older adults. When applying a specific cut point from a calibration study, it is important to follow the same data collection protocol used in the original study (e.g., model, epoch length, placement, age group) [24, 27].

In this review, most of the studies with toddlers, pre-schoolers and children did not use logs or diaries; whereas studies with adolescents, adults, and older adults, used diary logs, mainly to detect non-wear time, or sleep time. In fact, the use of diaries or logs is strongly recommended [34]. However, there is no consensus on the best diary to use for reporting MVPA, and its use in large sample studies may not be feasible.

Accelerometry has several advantages over self-report methods [22]. However, predetermined methodological decisions, such as data collection procedures and processing criteria, have a significant impact on the interpretation of accelerometer data [24, 27, 34]. Despite this, our review confirmed that several studies did not report all the methodological decisions, which is consistent with previous work [27]. Such reports cannot be accurately compared, making it difficult to interpret how different methodological decisions may have influenced the results. It is important that all methodological decisions regarding accelerometry are reported in future research reports to improve data comparability and reproducibility. However, with the continued advances in technology, the use of raw data from accelerometers has become increasingly prevalent in research, enabling a more nuanced and standardized analysis of MovBeh [176]. Raw data provides a direct measurement of acceleration in gravitational units (g). This shift towards raw data allows researchers to apply consistent processing algorithms across different devices, improving cross-study comparability [177–179]. The benefits of using raw data include greater flexibility in defining activity metrics such as intensity cut-points and the ability to apply sophisticated data processing methods like machine learning to enhance the classification of movement patterns [178]. Moreover, software tools like GGIR have facilitated this approach, offering an open-source solution that standardizes raw data processing and supports replication and transparency in research. Despite these advances, challenges remain in ensuring uniformity in sensor calibration, sampling frequency, and wear location, as these factors can introduce variability in raw data analysis [180].

A future consensus on methodological choices in accelerometry could combine the findings of this review and others that focus more on the quality of individual methodological choices [27, 30–32].

#### Strengths and Limitations

To the best of our knowledge, this is the first paper to review how researchers manage and describe the methodological procedures of accelerometry in studies measuring 24-h MovBeh across all age groups, using a scoping review design. This review comprehensively summarized the key methodological decisions regarding accelerometry in terms of data collection procedures and data processing, including a description of the cut-points and algorithms used in all included reports. This review adds another layer to the decisions that need to be made when preparing to collect data on 24-h MovBeh by helping researchers to define accelerometry protocols. The review also did not impose a date limit on the literature search, resulting in a review with a high number of included papers. As this review included all types of accelerometers (brands and models), the results should be interpreted with the understanding that different accelerometers have specific characteristics. It is also important to note that this review was limited to reporting the most common procedures for methodological decisions for accelerometry, without considering measurement properties and validation/calibration studies. Therefore, this review should not be considered as a list of best practices for the use and processing of accelerometry data. Rather, it should be seen as a compilation of the most commonly used methods in studies that measure all MovBeh over 24-h periods, which can be used as a useful resource in any future consensus on this topic.

## Conclusions

This review showed considerable heterogeneity between reports, highlighting the wide range of options available for assessing 24-h MovBeh using accelerometry and the lack of consensus between researchers. This review also showed that several reports did not disclose all methodological choices made to assess MovBeh. Therefore, in order to improve accuracy and allow comparability between studies and consensus on methodological choices, authors should report the main decisions made regarding accelerometry, i.e. the placement of the accelerometer on the body, the number of days for data collection, the number of valid days required, the time required for data to be considered valid; criteria for non-wear time, the epoch length, the sampling rate (i.e. Hertz) and the MovBeh measurement procedure, which is challenging given the complexity of the procedures, the number of brands and types of accelerometers available, and the plethora of programming options. A recommended next step, could be a future consensus on methodological choices in accelerometry, that combines the findings of this review and others that focus more on the quality of individual methodological choices.

## Supplementary Information

Additional file1.

Additional file2.

## Data Availability

The data are available upon reasonable request from the authors.

## Abbreviations

MovBeh: Movement behaviors
SB: Sedentary behaviors
PA: Physical activity
WHO: World health organization

## References

[1] Pedišić Ž, Dumuid D, Olds TS. Integrating sleep, sedentary behaviour, and physical activity research in the emerging field of time-use epidemiology: definitions, concepts, statistical methods, theoretical framework, and future directions. Kinesiology. 2017;49(2):252–69.

[2] Carson V, Tremblay MS, Chaput J-P, Chastin SF. Associations between sleep duration, sedentary time, physical activity, and health indicators among Canadian children and youth using compositional analyses. Appl Physiol Nutr Metab. 2016;41(6):S294–302. 27306435 10.1139/apnm-2016-0026

[3] Carson V, Chaput J-P, Janssen I, Tremblay MS. Health associations with meeting new 24-hour movement guidelines for Canadian children and youth. Prev Med. 2017;95:7–13. 27923668 10.1016/j.ypmed.2016.12.005

[4] Rollo S, Antsygina O, Tremblay MS. The whole day matters: understanding 24-hour movement guideline adherence and relationships with health indicators across the lifespan. J Sport Health Sci. 2020;9(6):493–510. 32711156 10.1016/j.jshs.2020.07.004 PMC7749249

[5] Janssen I, Clarke AE, Carson V, Chaput J-P, Giangregorio LM, Kho ME, et al. A systematic review of compositional data analysis studies examining associations between sleep, sedentary behaviour, and physical activity with health outcomes in adults. Appl Physiol Nutr Metab. 2020;45:S248–57. 33054342 10.1139/apnm-2020-0160

[6] Ekelund U, Steene-Johannessen J, Brown WJ, Fagerland MW, Owen N, Powell KE, et al. Does physical activity attenuate, or even eliminate, the detrimental association of sitting time with mortality? A harmonised meta-analysis of data from more than 1 million men and women. Lancet. 2016;388(10051):1302–10. 27475271 10.1016/S0140-6736(16)30370-1

[7] Okely AD, Ghersi D, Hesketh KD, Santos R, Loughran SP, Cliff DP, et al. A collaborative approach to adopting/adapting guidelines-the Australian 24-hour movement guidelines for the early years (birth to 5 years): an integration of physical activity, sedentary behavior, and sleep. BMC Public Health. 2017;17(5):869. 29219094 10.1186/s12889-017-4867-6 PMC5773882

[8] Tremblay MS, Carson V, Chaput J-P, Connor Gorber S, Dinh T, Duggan M, et al. Canadian 24-hour movement guidelines for children and youth: an integration of physical activity, sedentary behaviour, and sleep. Appl Physiol Nutr Metab. 2016;41(6):S311–27. 27306437 10.1139/apnm-2016-0151

[9] Tremblay MS, Chaput J-P, Adamo KB, Aubert S, Barnes JD, Choquette L, et al. Canadian 24-hour movement guidelines for the early years (0–4 years): an integration of physical activity, sedentary behaviour, and sleep. BMC Public Health. 2017;17(5):874. 29219102 10.1186/s12889-017-4859-6 PMC5773896

[10] Ross R, Chaput J-P, Giangregorio LM, Janssen I, Saunders TJ, Kho ME, et al. Canadian 24-hour movement guidelines for adults aged 18–64 years and adults aged 65 years or older: an integration of physical activity, sedentary behaviour, and sleep. Appl Physiol Nutr Metab. 2020;45:S57–102. 33054332 10.1139/apnm-2020-0467

[11] Draper CE, Tomaz SA, Biersteker L, Cook CJ, Couper J, de Milander M, et al. The South African 24-hour movement guidelines for birth to 5 years: an integration of physical activity, sitting behavior, screen time, and sleep. J Phys Act Health. 2020;17(1):109–19. 31877557 10.1123/jpah.2019-0187

[12] WHO. Guidelines on physical activity, sedentary behaviour and sleep for children under 5 years of age: world health organization. 2019. 31091057

[13] Troiano RP, Stamatakis E, Bull FC. How can global physical activity surveillance adapt to evolving physical activity guidelines? Needs, challenges and future directions. Br J Sports Med. 2020;54(24):1468–73. 33239352 10.1136/bjsports-2020-102621 PMC7719905

[14] Lubans DR, Hesketh K, Cliff D, Barnett L, Salmon J, Dollman J, et al. A systematic review of the validity and reliability of sedentary behaviour measures used with children and adolescents. Obes Rev. 2011;12(10):781–99. 21676153 10.1111/j.1467-789X.2011.00896.x

[15] Strath SJ, Kaminsky LA, Ainsworth BE, Ekelund U, Freedson PS, Gary RA, et al. Guide to the assessment of physical activity: clinical and research applications: a scientific statement from the American heart association. Circulation. 2013;128(20):2259–79. 24126387 10.1161/01.cir.0000435708.67487.da

[16] Meltzer LJ, Montgomery-Downs HE, Insana SP, Walsh CM. Use of actigraphy for assessment in pediatric sleep research. Sleep Med Rev. 2012;16(5):463–75. 22424706 10.1016/j.smrv.2011.10.002 PMC3445439

[17] Aminian S, Hinckson EA. Examining the validity of the ActivPAL monitor in measuring posture and ambulatory movement in children. Int J Behav Nutr Phys Act. 2012;9(1):1–9. 23031188 10.1186/1479-5868-9-119 PMC3490870

[18] Basterfield L, Adamson AJ, Pearce MS, Reilly JJ. Stability of habitual physical activity and sedentary behavior monitoring by accelerometry in 6-to 8-year-olds. J Phys Act Health. 2011;8(4):543–7. 21597127 10.1123/jpah.8.4.543

[19] Trost SG, Mciver KL, Pate RR. Conducting accelerometer-based activity assessments in field-based research. Med Sci Sports Exerc. 2005;37(11):S531–43. 16294116 10.1249/01.mss.0000185657.86065.98

[20] Sadeh A. The role and validity of actigraphy in sleep medicine: an update. Sleep Med Rev. 2011;15(4):259–67. 21237680 10.1016/j.smrv.2010.10.001

[21] Kinder JR, Lee KA, Thompson H, Hicks K, Topp K, Madsen KA. Validation of a hip-worn accelerometer in measuring sleep time in children. J Pediatr Nurs. 2012;27(2):127–33. 22341191 10.1016/j.pedn.2010.11.004 PMC3285433

[22] Warren JM, Ekelund U, Besson H, Mezzani A, Geladas N, Vanhees L. Assessment of physical activity–a review of methodologies with reference to epidemiological research: a report of the exercise physiology section of the European association of cardiovascular prevention and rehabilitation. Eur J Prev Cardiol. 2010;17(2):127–39. 10.1097/HJR.0b013e32832ed875 20215971

[23] Hills AP, Mokhtar N, Byrne NM. Assessment of physical activity and energy expenditure: an overview of objective measures. Front Nutr. 2014;1:5. 25988109 10.3389/fnut.2014.00005 PMC4428382

[24] Burchartz A, Anedda B, Auerswald T, Giurgiu M, Hill H, Ketelhut SI, et al. Assessing physical behavior through accelerometry–state of the science, best practices and future directions. Psychol Sport Exerc. 2020;49:101703. 10.1016/j.psychsport.2020.101742 PMC7430559 32831643

[25] Pedišić Ž, Bauman A. Accelerometer-based measures in physical activity surveillance: current practices and issues. Br J Sports Med. 2015;49(4):219–23. 25370153 10.1136/bjsports-2013-093407

[26] Clevenger KA, Montoye AH, Van Camp CA, Strath SJ, Pfeiffer KA. Methods for estimating physical activity and energy expenditure using raw accelerometry data or novel analytical approaches: a repository, framework, and reporting guidelines. Physiol Meas. 2022;43(9):09NT1. 10.1088/1361-6579/ac89c9 35970174

[27] Migueles C-SC, Ekelund U, Nyström CD, Mora-Gonzalez J, Löf M, et al. Accelerometer data collection and processing criteria to assess physical activity and other outcomes: a systematic review and practical considerations. Sports Med. 2017;47(9):1821–45. 28303543 10.1007/s40279-017-0716-0 PMC6231536

[28] Lippke S, Voelcker-Rehage C, Bültmann U. Assessing your client’s physical activity behavior, motivation, and individual resources. ACSM's behavioral aspects of physical activity and exercise Philadelphia: Wolters Kluwer Health/Lippincott Williams & Wilkins. 2013:39–69.

[29] Rodrigues B, Júdice PB, Marques A, Carraça EV, Lopes L, Sousa-Sá E, et al. 24-hour movement questionnaire (QMov24h) for adults: development process and measurement properties. Int J Behav Nutr Phys Act. 2024;21(1):116. 39385225 10.1186/s12966-024-01667-7 PMC11466043

[30] de Vries SI, Bakker I, Hopman-Rock M, Hirasing RA, van Mechelen W. Clinimetric review of motion sensors in children and adolescents. J Clin Epidemiol. 2006;59(7):670–80. 16765269 10.1016/j.jclinepi.2005.11.020

[31] Lettink A, Altenburg TM, Arts J, van Hees VT, Chinapaw MJM. Systematic review of accelerometer-based methods for 24-h physical behavior assessment in young children (0–5 years old). Int J Behav Nutr Phys Act. 2022;19(1):116. 36076221 10.1186/s12966-022-01296-y PMC9461103

[32] Lynch BA, Kaufman TK, Rajjo TI, Mohammed K, Kumar S, Murad MH, et al. Accuracy of accelerometers for measuring physical activity and levels of sedentary behavior in children: a systematic review. J Prim Care Comm Health. 2019;10:2150132719874252. 10.1177/2150132719874252 PMC6740055 31509061

[33] Migueles JH, Aadland E, Andersen LB, Brønd JC, Chastin SF, Hansen BH, et al. GRANADA consensus on analytical approaches to assess associations with accelerometer-determined physical behaviours (physical activity, sedentary behaviour and sleep) in epidemiological studies. Br J Sports Med. 2022;56(7):376–84. 33846158 10.1136/bjsports-2020-103604 PMC8938657

[34] Quante M, Kaplan ER, Rueschman M, Cailler M, Buxton OM, Redline S. Practical considerations in using accelerometers to assess physical activity, sedentary behavior, and sleep. Sleep Health. 2015;1(4):275–84. 29073403 10.1016/j.sleh.2015.09.002

[35] Cliff DP, Reilly JJ, Okely AD. Methodological considerations in using accelerometers to assess habitual physical activity in children aged 0–5 years. J Sci Med Sport. 2009;12(5):557–67. 19147404 10.1016/j.jsams.2008.10.008

[36] Murphy SL. Review of physical activity measurement using accelerometers in older adults: considerations for research design and conduct. Prev Med. 2009;48(2):108–14. 19111780 10.1016/j.ypmed.2008.12.001 PMC10071821

[37] Tricco AC, Lillie E, Zarin W, O’Brien KK, Colquhoun H, Levac D, et al. PRISMA extension for scoping reviews (PRISMA-ScR): checklist and explanation. Ann Intern Med. 2018;169(7):467–73. 30178033 10.7326/M18-0850

[38] Arksey H, O’Malley L. Scoping studies: towards a methodological framework. Int J Soc Res Methodol. 2005;8(1):19–32.

[39] Levac D, Colquhoun H, O’Brien KK. Scoping studies: advancing the methodology. Implement Sci. 2010;5:1–9. 20854677 10.1186/1748-5908-5-69 PMC2954944

[40] Kohl C, McIntosh EJ, Unger S, Haddaway NR, Kecke S, Schiemann J, et al. Online tools supporting the conduct and reporting of systematic reviews and systematic maps: a case study on CADIMA and review of existing tools. Environ Evid. 2018;7(1):1–17.

[41] Armstrong B, Covington LB, Hager ER, Black MM. Objective sleep and physical activity using 24-hour ankle-worn accelerometry among toddlers from low-income families. Sleep Health. 2019;5(5):459–65. 31171491 10.1016/j.sleh.2019.04.005 PMC6801033

[42] Santos R, Zhang Z, Pereira JR, Sousa-Sá E, Cliff DP, Okely AD. Compliance with the Australian 24-hour movement guidelines for the early years: associations with weight status. BMC Public Health. 2017;17(Suppl 5):867. 29219095 10.1186/s12889-017-4857-8 PMC5773912

[43] Haines J, Douglas S, Mirotta JA, O’Kane C, Breau R, Walton K, et al. Guelph family health study: pilot study of a home-based obesity prevention intervention. Can J Public Health. 2018;109(4):549–60. 29981086 10.17269/s41997-018-0072-3 PMC6964565

[44] Alonso-Martínez AM, Ramírez-Vélez R, García-Alonso Y, Izquierdo M, García-Hermoso A. Physical activity, sedentary behavior, sleep and self-regulation in Spanish preschoolers during the covid-19 lockdown. Int J Environ Res Public Health. 2021;18(2):693. 33467399 10.3390/ijerph18020693 PMC7830291

[45] Chang Z, Lei W. A study on the relationship between physical activity, sedentary behavior, and sleep duration in preschool children. Front Public Health. 2021;9:618962. 33898373 10.3389/fpubh.2021.618962 PMC8059703

[46] Chen B, Bernard JY, Padmapriya N, Ning Y, Cai S, Lança C, et al. Associations between early-life screen viewing and 24 hour movement behaviours: findings from a longitudinal birth cohort study. Lancet Child Adolesc Health. 2020;4(3):201–9. 32004497 10.1016/S2352-4642(19)30424-9

[47] De Craemer M, Decraene M, Willems I, Buysse F, Van Driessche E, Verbestel V. Objective measurement of 24-hour movement behaviors in preschool children using wrist-worn and thigh-worn accelerometers. Int J Environ Res Public Health. 2021;18(18):9482. 34574402 10.3390/ijerph18189482 PMC8471723

[48] Duraccio KM, Jensen CD. Associations between physical and sedentary activity regularity and sleep in preschoolers and kindergartners. Sleep Health. 2017;3(4):263–8. 28709513 10.1016/j.sleh.2017.04.001

[49] Ng JYY, He Q, Chong KH, Okely AD, Chan CHS, Ha AS. The impact of COVID-19 on preschool-aged children’s movement behaviors in hong kong: a longitudinal analysis of accelerometer-measured data. Int J Environ Res Public Health. 2021;18(22):11907. 34831662 10.3390/ijerph182211907 PMC8623956

[50] Tomaz SA, Jones RA, Hinkley T, Twine R, Kahn K, Norris SA, et al. Physical activity in early childhood education and care settings in a low-income, rural South African community: an observational study. Rural Remote Health. 2019;19(4):5249. 31670971 10.22605/RRH5249

[51] Meredith-Jones K, Galland B, Haszard J, Gray A, Sayers R, Hanna M, et al. Do young children consistently meet 24-h sleep and activity guidelines? A longitudinal analysis using actigraphy. Int J Obes (Lond). 2019;43(12):2555–64. 31477783 10.1038/s41366-019-0432-y

[52] St Laurent CW, Burkart S, Rodheim K, Marcotte R, Spencer RMC. Cross-sectional associations of 24-hour sedentary time, physical activity, and sleep duration compositions with sleep quality and habits in preschoolers. Int J Environ Res Public Health. 2020;17(19):7148. 33003598 10.3390/ijerph17197148 PMC7579350

[53] Williams SM, Farmer VL, Taylor BJ, Taylor RW. Do more active children sleep more? A repeated cross-sectional analysis using accelerometry. PLoS ONE. 2014;9(4):e93117. 24695112 10.1371/journal.pone.0093117 PMC3973701

[54] Butte NF, Puyau MR, Wilson TA, Liu Y, Wong WW, Adolph AL, et al. Role of physical activity and sleep duration in growth and body composition of preschool-aged children. Obesity. 2016;24(6):1328–35. 27087679 10.1002/oby.21489 PMC4882246

[55] Hossain MS, Deeba IM, Hasan M, Kariippanon KE, Chong KH, Cross PL, et al. International study of 24-h movement behaviors of early years (SUNRISE): a pilot study from Bangladesh. Pilot Feasibility Stud. 2021;7(1):176. 34526148 10.1186/s40814-021-00912-1 PMC8440144

[56] Kang AW, Gans KM, Minkel J, Risica PM. Correlates of objectively measured sleep and physical activity among Latinx 3-To-5-year old children. J Pediatr Nurs. 2021;60:40–5. 33618176 10.1016/j.pedn.2021.01.010

[57] Kuzik N, Naylor PJ, Spence JC, Carson V. Movement behaviours and physical, cognitive, and social-emotional development in preschool-aged children: cross-sectional associations using compositional analyses. PLoS ONE. 2020;15(8):e0237945. 32810172 10.1371/journal.pone.0237945 PMC7433874

[58] Guan H, Zhang Z, Wang B, Okely AD, Tong M, Wu J, et al. Proportion of kindergarten children meeting the WHO guidelines on physical activity, sedentary behaviour and sleep and associations with adiposity in urban Beijing. BMC Pediatr. 2020;20(1):70. 32061263 10.1186/s12887-020-1969-6 PMC7023817

[59] Manyanga T, Barnes JD, Chaput JP, Katzmarzyk PT, Prista A, Tremblay MS. Prevalence and correlates of adherence to movement guidelines among urban and rural children in mozambique: a cross-sectional study. Int J Behav Nutr Phys Act. 2019;16(1):94. 31661004 10.1186/s12966-019-0861-y PMC6819612

[60] Ekstedt M, Nyberg G, Ingre M, Ekblom Ö, Marcus C. Sleep, physical activity and BMI in six to ten-year-old children measured by accelerometry: a cross-sectional study. Int J Behav Nutr Phys Act. 2013;10:82. 23800204 10.1186/1479-5868-10-82 PMC3691618

[61] Padmapriya N, Chen B, Goh C, Shek LPC, Chong YS, Tan KH, et al. 24-hour movement behaviour profiles and their transition in children aged 5.5 and 8 years—findings from a prospective cohort study. Int J Behav Nutr Phys Act. 2021;18(1):145. 34742314 10.1186/s12966-021-01210-y PMC8572484

[62] Taylor RW, Haszard JJ, Farmer VL, Richards R, Te Morenga L, Meredith-Jones K, et al. Do differences in compositional time use explain ethnic variation in the prevalence of obesity in children? Analyses using 24-hour accelerometry. Int J Obes (Lond). 2020;44(1):94–103. 31089262 10.1038/s41366-019-0377-1

[63] Vuholm S, Teisen MN, Mølgaard C, Lauritzen L, Damsgaard CT. Sleep and physical activity in healthy 8-9-year-old children are affected by oily fish consumption in the FiSK Junior randomized trial. Eur J Nutr. 2021;60(6):3095–106. 33515093 10.1007/s00394-021-02490-7

[64] Burkart S, Parker H, Weaver RG, Beets MW, Jones A, Adams EL, et al. Impact of the COVID-19 pandemic on elementary schoolers’ physical activity, sleep, screen time and diet: a quasi-experimental interrupted time series study. Pediatr Obes. 2022;17(1):12846. 10.1111/ijpo.12846 PMC8420216 34409754

[65] Haszard JJ, Meredith-Jones K, Farmer V, Williams S, Galland B, Taylor R. Non-wear time and presentation of compositional 24-hour time-use analyses influence conclusions about sleep and body mass index in children. J Meas Phys Behav. 2020;3(3):204–10.

[66] Toledo-Vargas M, Perez-Contreras P, Chandia-Poblete D, Aguilar-Farias N. Compliance of the 24-hour movement guidelines in 9- to 11-year-old children from a low-income town in Chile. J Phys Act Health. 2020;17(10):1034–41. 32866944 10.1123/jpah.2019-0672

[67] Armstrong B, Beets MW, Starrett A, Brazendale K, Turner-McGrievy G, Saelens BE, et al. Dynamics of sleep, sedentary behavior, and moderate-to-vigorous physical activity on school versus nonschool days. Sleep J Sleep Sleep Disord Res. 2021;44(2):1–12. 10.1093/sleep/zsaa174 PMC7982135 32893864

[68] Fairclough SJ, Dumuid D, Taylor S, Curry W, McGrane B, Stratton G, et al. Fitness, fatness and the reallocation of time between children’s daily movement behaviours: an analysis of compositional data. Int J Behav Nutr Phys Act. 2017;14(1):64. 28486972 10.1186/s12966-017-0521-z PMC5424384

[69] Hjorth MF, Sørensen LB, Andersen R, Dyssegaard CB, Ritz C, Tetens I, et al. Normal weight children have higher cognitive performance—independent of physical activity, sleep, and diet. Physiol Behav. 2016;165:398–404. 27570193 10.1016/j.physbeh.2016.08.021

[70] Moreno JP, Razjouyan J, Lester H, Dadabhoy H, Amirmazaheri M, Reesor-Oyer L, et al. Later sleep timing predicts accelerated summer weight gain among elementary school children: a prospective observational study. Int J Behav Nutr Phys Act. 2021;18(1):94. 34247639 10.1186/s12966-021-01165-0 PMC8273994

[71] Antczak D, Sanders T, Del Pozo CB, Parker P, Lonsdale C. Day-to-day and longer-term longitudinal associations between physical activity, sedentary behavior, and sleep in children. Sleep. 2021;44(4):zsaa219. 33103724 10.1093/sleep/zsaa219

[72] García-Alonso Y, García-Hermoso A, Izquierdo M, Legarra-Gorgoñon G, Ramírez-Vélez R, Alonso-Martínez AM. Relationship between parents’ and children’s objectively assessed movement behaviours prior to and during the COVID-19 pandemic. Pediatr Obes. 2022;17(9):e12923. 35488779 10.1111/ijpo.12923 PMC9347505

[73] Hedayatrad L, Stewart T, Paine SJ, Marks E, Walker C, Duncan S. Sociodemographic differences in 24-hour time-use behaviours in New Zealand children. Int J Behav Nutr Phys Act. 2022;19(1):131. 36195954 10.1186/s12966-022-01358-1 PMC9531491

[74] Lucas-de la Cruz L, Martínez-Vizcaíno V, Cañete García-Prieto J, Arias-Palencia N, Diez-Fernandez A, Milla-Tobarra M, et al. Movement behaviors and cardiometabolic risk in schoolchildren. PLoS ONE. 2018;13(11):e0207300. 30427939 10.1371/journal.pone.0207300 PMC6235312

[75] Martinez SM, Tschann JM, McCulloch CE, Sites E, Butte NF, Gregorich SE, et al. Temporal associations between circadian sleep and activity patterns in Mexican American children. Sleep Health. 2019;5(2):201–7. 30928122 10.1016/j.sleh.2018.10.012 PMC6442936

[76] Caetano IT, Miranda VPN, Dos Santos FK, Dos Santos Amorim PR. Adolescent’s movement behaviors and built environment: a latent class analysis. BMC Public Health. 2021;21(1):1937. 34696762 10.1186/s12889-021-11974-4 PMC8547093

[77] Chong KH, Parrish AM, Cliff DP, Dumuid D, Okely AD. Cross-sectional and longitudinal associations between 24-hour movement behaviours, recreational screen use and psychosocial health outcomes in children: a compositional data analysis approach. Int J Environ Res Public Health. 2021;18(11):5995. 34204928 10.3390/ijerph18115995 PMC8199728

[78] da Costa BGG, Chaput JP, Lopes MVV, Malheiros LEA, Silva KS. How do adolescents with short sleep duration spend their extra waking hours? a device-based analysis of physical activity and sedentary behaviour in a Brazilian sample. Sleep Sci. 2021;14(Spec 2):163–6. 35082986 10.5935/1984-0063.20200100 PMC8764950

[79] Dumuid D, Wake M, Burgner D, Tremblay MS, Okely AD, Edwards B, et al. Balancing time use for children’s fitness and adiposity: evidence to inform 24-hour guidelines for sleep, sedentary time and physical activity. PLoS ONE. 2021;16(1):e0245501. 33465128 10.1371/journal.pone.0245501 PMC7815105

[80] Negele L, Flexeder C, Koletzko S, Bauer CP, von Berg A, Berdel D, Schikowski T, Standl M, Peters A, Schulz H. Association between objectively assessed physical activity and sleep quality in adolescence. Results from the GINIplus and LISA studies. Sleep Med. 2020;72:65–74. 32554326 10.1016/j.sleep.2020.03.007

[81] Van Dyk TR, Krietsch KN, Saelens BE, Whitacre C, McAlister S, Beebe DW. Inducing more sleep on school nights reduces sedentary behavior without affecting physical activity in short-sleeping adolescents. Sleep Med. 2018;47:7–10. 29880148 10.1016/j.sleep.2018.03.007

[82] Fairclough SJ, Tyler R, Dainty JR, Dumuid D, Richardson C, Shepstone L, et al. Cross-sectional associations between 24-hour activity behaviours and mental health indicators in children and adolescents: a compositional data analysis. J Sports Sci. 2021;39(14):1602–14. 33615990 10.1080/02640414.2021.1890351

[83] Collings PJ, Wijndaele K, Corder K, Westgate K, Ridgway CL, Sharp SJ, et al. Magnitude and determinants of change in objectively-measured physical activity, sedentary time and sleep duration from ages 15 to 175y in UK adolescents: the roots study. Int J Behav Nutr Phys Act. 2015;12:1–10. 25971606 10.1186/s12966-015-0222-4 PMC4437669

[84] Gába A, Dygrýn J, Štefelová N, Rubín L, Hron K, Jakubec L, et al. How do short sleepers use extra waking hours? A compositional analysis of 24-h time-use patterns among children and adolescents. Int J Behav Nutr Phys Act. 2020;17(1):104. 32795287 10.1186/s12966-020-01004-8 PMC7427741

[85] Kim Y, Umeda M, Lochbaum M, Sloan RA. Examining the day-to-day bidirectional associations between physical activity, sedentary behavior, screen time, and sleep health during school days in adolescents. PLoS ONE. 2020;15(9):e0238721. 32881930 10.1371/journal.pone.0238721 PMC7470331

[86] Talarico R, Janssen I. Compositional associations of time spent in sleep, sedentary behavior and physical activity with obesity measures in children. Int J Obes (Lond). 2018;42(8):1508–14. 29568110 10.1038/s41366-018-0053-x

[87] Starbek P, Kastelic K, Šarabon N. The impact of online-schooling during COVID-19 on device-measured 24-hour movement behaviours among high school students: a compositional data analysis. Children (Basel). 2022;9(5):667. 35626844 10.3390/children9050667 PMC9139799

[88] Grant VM, Tomayko EJ, Kingfisher RD. Sleep and physical activity patterns in urban American Indian children. Am J Health Behav. 2020;44(1):67–75. 31783933 10.5993/AJHB.44.1.7 PMC7373171

[89] Beltran-Valls MR, Adelantado-Renau M, Mota J, Moliner-Urdiales D. Longitudinal associations of healthy behaviors on fitness in adolescents: DADOS study. Am J Prev Med. 2021;61(3):410–7. 34210583 10.1016/j.amepre.2021.04.009

[90] Domingues SF, Diniz da Silva C, Faria FR, de Sá SH, Dos Santos Amorim PR. Sleep, sedentary behavior, and physical activity in Brazilian adolescents: achievement recommendations and BMI associations through compositional data analysis. PLoS ONE. 2022;17(4):e0266926. 35404979 10.1371/journal.pone.0266926 PMC9000056

[91] Harrington DM, Ioannidou E, Davies MJ, Edwardson CL, Gorely T, Rowlands AV, et al. Concurrent screen use and cross-sectional association with lifestyle behaviours and psychosocial health in adolescent females. Acta Paediatr. 2021;110(7):2164–70. 33570799 10.1111/apa.15806 PMC9134851

[92] Aguilar-Farias N, Martino-Fuentealba P, Chandia-Poblete D. Correlates of device-measured physical activity, sedentary behaviour and sleeping in children aged 9–11 years from Chile: ESPACIOS study. / Factores asociados con actividad física, conducta sedentaria y sueño medidos con acelerómetros en niños de 9–11 años de Chile: estudio ESPACIOS. Retos: Nuevas Perspectivas de Educación Física, Deporte y Recreación. 2020;37:1–10.

[93] Butte NF, Puyau MR, Adolph AL, Vohra FA, Zakeri I. Physical activity in nonoverweight and overweight Hispanic children and adolescents. Med Sci Sports Exerc. 2007;39(8):1257–66. 17762358 10.1249/mss.0b013e3180621fb6

[94] Krietsch KN, Duraccio KM, Zhang N, Saelens BE, Howarth T, Combs A, et al. Earlier bedtimes and more sleep displace sedentary behavior but not moderate-to-vigorous physical activity in adolescents. Sleep Health. 2022;8(3):270–6. 35461788 10.1016/j.sleh.2022.01.003 PMC12459484

[95] Master L, Nye RT, Lee S, Nahmod NG, Mariani S, Hale L, et al. Bidirectional, daily temporal associations between sleep and physical activity in adolescents. Sci Rep. 2019;9(1):7732. 31118441 10.1038/s41598-019-44059-9 PMC6531611

[96] Merikanto I, Kuula L, Lahti J, Räikkönen K, Pesonen AK. Eveningness associates with lower physical activity from pre- to late adolescence. Sleep Med. 2020;74:189–98. 32858279 10.1016/j.sleep.2020.07.021

[97] Ataka T, Kimura N, Eguchi A, Matsubara E. Changes in objectively measured lifestyle factors during the COVID-19 pandemic in community-dwelling older adults. BMC Geriatr. 2022;22(1):326. 35421951 10.1186/s12877-022-03043-1 PMC9008373

[98] Betson JR, Kirkcaldie MTK, Zosky GR, Ross RM. Transition to shift work: sleep patterns, activity levels, and physiological health of early-career paramedics. Sleep Health. 2022;8(5):514–20. 35907709 10.1016/j.sleh.2022.06.001

[99] Blodgett JM, Mitchell JJ, Stamatakis E, Chastin S, Hamer M. Associations between the composition of daily time spent in physical activity, sedentary behaviour and sleep and risk of depression: compositional data analyses of the 1970 British cohort study. J Affect Disord. 2022;320:616–20. 36183826 10.1016/j.jad.2022.09.110

[100] Booth JN, Bromley LE, Darukhanavala AP, Whitmore HR, Imperial JG, Penev PD. Reduced physical activity in adults at risk for type 2 diabetes who curtail their sleep. Obesity. 2012;20(2):278–84. 21996665 10.1038/oby.2011.306 PMC3262101

[101] Buman MP, Hu F, Newman E, Smeaton AF, Epstein DR. Behavioral periodicity detection from 24 h wrist accelerometry and associations with cardiometabolic risk and health-related quality of life. Biomed Res Int. 2016;2016:4856506. 26942195 10.1155/2016/4856506 PMC4752978

[102] Cabanas-Sánchez V, Martínez-Gómez D, Esteban-Cornejo I, Castro-Piñero J, Conde-Caveda J, Veiga ÓL. Reliability and validity of the youth leisure-time sedentary behavior questionnaire (YLSBQ). J Sci Med Sport. 2018;21(1):69–74. 29129459 10.1016/j.jsams.2017.10.031

[103] Cabanas-Sánchez V, Esteban-Cornejo I, Migueles JH, Banegas JR, Graciani A, Rodríguez-Artalejo F, et al. Twenty four-hour activity cycle in older adults using wrist-worn accelerometers: the seniors-ENRICA-2 study. Scand J Med Sci Sports. 2020;30(4):700–8. 31834945 10.1111/sms.13612

[104] Carneiro-Barrera A, Amaro-Gahete FJ, Acosta FM, Ruiz JR. Body composition impact on sleep in young adults: the mediating role of sedentariness, physical activity, and diet. J Clin Med. 2020;9(5):1560. 32455844 10.3390/jcm9051560 PMC7290677

[105] Dumuid D, Lewis LK, Olds TS, Maher C, Bondarenko C, Norton L. Relationships between older adults’ use of time and cardio-respiratory fitness, obesity and cardio-metabolic risk: a compositional isotemporal substitution analysis. Maturitas. 2018;110:104–10. 29563028 10.1016/j.maturitas.2018.02.003

[106] Ellingson LD, Meyer JD, Shook RP, Dixon PM, Hand GA, Wirth MD, et al. Changes in sedentary time are associated with changes in mental wellbeing over 1 year in young adults. Prev Med Rep. 2018;11:274–81. 30116698 10.1016/j.pmedr.2018.07.013 PMC6082791

[107] Full KM, Gallo LC, Malhotra A, Bellettiere J, Kerr J, Arredondo E, et al. Modeling the cardiometabolic benefits of sleep in older women: exploring the 24-hour day. Sleep. 2020;43(1):zsz205. 31553045 10.1093/sleep/zsz205 PMC6955642

[108] Liao Y, Robertson MC, Winne A, Wu IHC, Le TA, Balachandran DD, et al. Investigating the within-person relationships between activity levels and sleep duration using fitbit data. Transl Behav Med. 2021;11(2):619–24. 32667039 10.1093/tbm/ibaa071 PMC7963288

[109] Millard LAC, Tilling K, Gaunt TR, Carslake D, Lawlor DA. Association of physical activity intensity and bout length with mortality: an observational study of 79,503 UK Biobank participants. PLoS Med. 2021;18(9):e1003757. 34525088 10.1371/journal.pmed.1003757 PMC8480840

[110] Mochón-Benguigui S, Carneiro-Barrera A, Castillo MJ, Amaro-Gahete FJ. Role of physical activity and fitness on sleep in sedentary middle-aged adults: the FIT-AGEING study. Sci Rep. 2021;11(1):539. 33436671 10.1038/s41598-020-79355-2 PMC7804461

[111] Park C, Larsen B, Kwon S, Xia Y, Dickson VV, Kim SS, et al. Acculturation, discrimination and 24-h activity in Asian American immigrant women. J Immigr Minor Health. 2022;24(4):1005–12. 35434771 10.1007/s10903-022-01361-5

[112] Park JH, Kim Y, Welk GJ, Silva P, Lee JM. Association with temperature variability and physical activity, sedentary behavior, and sleep in a free-living population. Int J Environ Res Public Health. 2021;18(24):13077. 34948687 10.3390/ijerph182413077 PMC8701207

[113] Suorsa K, Leskinen T, Pasanen J, Pulakka A, Myllyntausta S, Pentti J, et al. Changes in the 24-h movement behaviors during the transition to retirement: compositional data analysis. Int J Behav Nutr Phys Act. 2022;19(1):121. 36109809 10.1186/s12966-022-01364-3 PMC9479436

[114] Curtis RG, Dumuid D, Olds T, Plotnikoff R, Vandelanotte C, Ryan J, et al. The association between time-use behaviors and physical and mental well-being in adults: a compositional isotemporal substitution analysis. J Phys Act Health. 2020;17(2):197–203. 31918406 10.1123/jpah.2018-0687

[115] Cabanas-Sánchez V, Martínez-Gómez D, Izquierdo-Gómez R, Segura-Jiménez V, Castro-Piñero J, Veiga OL. Association between clustering of lifestyle behaviors and health-related physical fitness in youth: the UP&DOWN study. J Pediatr. 2018;199:41-8.e1. 29803300 10.1016/j.jpeds.2018.03.075

[116] Husu P, Tokola K, Vähä-Ypyä H, Sievänen H, Suni J, Heinonen OJ, et al. Physical activity, sedentary behavior, and time in bed among finnish adults measured 24/7 by triaxial accelerometry. J Meas Phys Behav. 2021;4(2):163–73.

[117] Knaeps S, De Baere S, Bourgois J, Mertens E, Charlier R, Lefevre J. Substituting sedentary time with light and moderate to vigorous physical activity is associated with better cardiometabolic health. J Phys Act Health. 2018;15(3):197–203. 28872401 10.1123/jpah.2017-0102

[118] Mitchell JA, Godbole S, Moran K, Murray K, James P, Laden F, et al. No evidence of reciprocal associations between daily sleep and physical activity. Med Sci Sports Exerc. 2016;48(10):1950–6. 27285490 10.1249/MSS.0000000000001000 PMC5026562

[119] Powell C, Browne LD, Carson BP, Dowd KP, Perry IJ, Kearney PM, et al. Use of compositional data analysis to show estimated changes in cardiometabolic health by reallocating time to light-intensity physical activity in older adults. Sports Med. 2020;50(1):205–17. 31350674 10.1007/s40279-019-01153-2

[120] Gupta N, Dencker-Larsen S, Lund Rasmussen C, McGregor D, Rasmussen CDN, Thorsen SV, et al. The physical activity paradox revisited: a prospective study on compositional accelerometer data and long-term sickness absence. Int J Behav Nutr Phys Act. 2020;17:1–9. 32690043 10.1186/s12966-020-00988-7 PMC7370435

[121] Verhoog S, Braun KVE, Bano A, van Rooij FJA, Franco OH, Koolhaas CM, et al. Associations of activity and sleep with quality of life: a compositional data analysis. Am J Prev Med. 2020;59(3):412–9. 32713616 10.1016/j.amepre.2020.03.029

[122] Full KM, Moran K, Carlson J, Godbole S, Natarajan L, Hipp A, et al. Latent profile analysis of accelerometer-measured sleep, physical activity, and sedentary time and differences in health characteristics in adult women. PLoS ONE. 2019;14(6):e0218595. 31247051 10.1371/journal.pone.0218595 PMC6597058

[123] Galmes-Panades AM, Varela-Mato V, Konieczna J, Wärnberg J, Martínez-González M, Salas-Salvadó J, et al. Isotemporal substitution of inactive time with physical activity and time in bed: cross-sectional associations with cardiometabolic health in the PREDIMED-Plus study. Int J Behav Nutr Phys Act. 2019;16(1):137. 31870449 10.1186/s12966-019-0892-4 PMC6929461

[124] German C, Makarem N, Fanning J, Redline S, Elfassy T, McClain A, et al. Reallocating sedentary behavior with sleep or physical activity is associated with favorable cardiovascular health in the multi-ethnic study of atherosclerosis: MESA. Circulation. 2020;141(SUPPL 1):1.

[125] Gilson ND, Mielke GI, Coombes JS, Feter N, Smith E, Duncan MJ, et al. VO(2peak) and 24-hour sleep, sedentary behavior, and physical activity in Australian truck drivers. Scand J Med Sci Sports. 2021;31(7):1574–8. 33793972 10.1111/sms.13965

[126] Goncin N, Linares A, Lloyd M, Dogra S. Does sedentary time increase in older adults in the days following participation in intense exercise? Aging Clin Exp Res. 2020;32(12):2517–27. 32130714 10.1007/s40520-020-01502-6

[127] Gubelmann C, Heinzer R, Haba-Rubio J, Vollenweider P, Marques-Vidal P. Physical activity is associated with higher sleep efficiency in the general population: the CoLaus study. Sleep J Sleep Sleep Disord Res. 2018;41(7):1–9. 10.1093/sleep/zsy070 29617980

[128] Gupta N, Dumuid D, Korshøj M, Jørgensen MB, Søgaard K, Holtermann A. Is daily composition of movement behaviors related to blood pressure in working adults? Med Sci Sports Exerc. 2018;50(10):2150–5. 30222689 10.1249/MSS.0000000000001680

[129] Hargens TA, Scott MC, Olijar V, Bigman M, Edwards ES. Markers of poor sleep quality increase sedentary behavior in college students as derived from accelerometry. Sleep Breath. 2021;25(1):537–44. 32948936 10.1007/s11325-020-02190-2

[130] Heiland EG, Ekblom Ö, Bojsen-Møller E, Larisch LM, Blom V, Ekblom MM. Bi-directional, day-to-day associations between objectively-measured physical activity, sedentary behavior, and sleep among office workers. Int J Environ Res Public Health. 2021;18(15):7999. 34360287 10.3390/ijerph18157999 PMC8345408

[131] Imes CC, Bizhanova Z, Kline CE, Rockette-Wagner B, Chasens ER, Sereika SM, et al. Bidirectional relationship between sleep and sedentary behavior in adults with overweight or obesity: a secondary analysis. Sleep Adv. 2021;2(1):zpab004. 33870194 10.1093/sleepadvances/zpab004 PMC8038645

[132] Le F, Yap Y, Tung NYC, Bei B, Wiley JF. The associations between daily activities and affect: a compositional isotemporal substitution analysis. Int J Behav Med. 2022;29(4):456–68. 34608593 10.1007/s12529-021-10031-z

[133] Lee J, Walker ME, Matthews KA, Kuller LH, Ranjit N, Gabriel KP. Associations of physical activity and sleep with cardiometabolic risk in older women. Prev Med Rep. 2020;18:101071. 32226729 10.1016/j.pmedr.2020.101071 PMC7093830

[134] Madden KM, Ashe MC, Lockhart C, Chase JM. Sedentary behavior and sleep efficiency in active community-dwelling older adults. Sleep Sci. 2014;7(2):82–8. 26483908 10.1016/j.slsci.2014.09.009 PMC4521656

[135] McDonough DJ, Helgeson MA, Liu W, Gao Z. Effects of a remote, YouTube-delivered exercise intervention on young adults’ physical activity, sedentary behavior, and sleep during the covid-19 pandemic: randomized controlled trial. J Sport Health Sci. 2022;11(2):145–56. 34314877 10.1016/j.jshs.2021.07.009 PMC8487769

[136] Meyer JD, Ellingson LD, Buman MP, Shook RP, Hand GA, Blair SN. Current and 1-year psychological and physical effects of replacing sedentary time with time in other behaviors. Am J Prev Med. 2020;59(1):12–20. 32418803 10.1016/j.amepre.2020.02.018

[137] Pasanen J, Leskinen T, Suorsa K, Pulakka A, Virta J, Auranen K, et al. Effects of physical activity intervention on 24-h movement behaviors: a compositional data analysis. Sci Rep. 2022;12(1):8712. 35610297 10.1038/s41598-022-12715-2 PMC9130120

[138] Seol J, Abe T, Fujii Y, Joho K, Okura T. Effects of sedentary behavior and physical activity on sleep quality in older people: a cross-sectional study. Nurs Health Sci. 2020;22(1):64–71. 31523925 10.1111/nhs.12647

[139] Tigbe WW, Granat MH, Sattar N, Lean MEJ. Time spent in sedentary posture is associated with waist circumference and cardiovascular risk. Int J Obes (Lond). 2017;41(5):689–96. 28138134 10.1038/ijo.2017.30

[140] Wang R, Blom V, Nooijen CFJ, Kallings LV, Ekblom Ö, Ekblom MM. The role of executive function in the effectiveness of multi-component interventions targeting physical activity behavior in office workers. Int J Environ Res Public Health. 2021;19(1):266. 35010526 10.3390/ijerph19010266 PMC8751160

[141] Santos AM, Ribeiro SL, Sousa AV, Machado DD, Monteiro PA, Moura P, Martins CM, Freitas IF, Santos MA, Rossi FE. Are there differences between male and female badminton athletes in sleep, physical activity and sedentary time? Rev Br Med Esporte. 2021;27(2):174–8.

[142] Pate RR, Almeida MJ, McIver KL, Pfeiffer KA, Dowda M. Validation and calibration of an accelerometer in preschool children. Obesity. 2006;14(11):2000–6. 17135617 10.1038/oby.2006.234

[143] Sadeh A, Sharkey M, Carskadon MA. Activity-based sleep-wake identification: an empirical test of methodological issues. Sleep. 1994;17(3):201–7. 7939118 10.1093/sleep/17.3.201

[144] Evenson KR, Catellier DJ, Gill K, Ondrak KS, McMurray RG. Calibration of two objective measures of physical activity for children. J Sports Sci. 2008;26(14):1557–65. 18949660 10.1080/02640410802334196

[145] Tye LS, Scott T, Haszard JJ, Peddie MC. Physical activity, sedentary behaviour and sleep, and their association with bmi in a sample of adolescent females in New Zealand. Int J Environ Res Public Health. 2020;17(17):6346. 32878296 10.3390/ijerph17176346 PMC7503577

[146] Hildebrand MV, van Hees VT, Hansen BH, Ekelund U. Age group comparability of raw accelerometer output from wrist-and hip-worn monitors. Med Sci Sports Exerc. 2014;46(9):1816–24. 24887173 10.1249/MSS.0000000000000289

[147] Hildebrand M, Hansen BH, van Hees VT, Ekelund U. Evaluation of raw acceleration sedentary thresholds in children and adults. Scand J Med Sci Sports. 2017;27(12):1814–23. 27878845 10.1111/sms.12795

[148] Cole RJ, Kripke DF, Gruen W, Mullaney DJ, Gillin JC. Automatic sleep/wake identification from wrist activity. Sleep. 1992;15(5):461–9. 1455130 10.1093/sleep/15.5.461

[149] Freedson PS, Melanson E, Sirard J. Calibration of the computer science and applications, inc. accelerometer. Med Sci Sports Exerc. 1998;30(5):777–81. 9588623 10.1097/00005768-199805000-00021

[150] Smith C, Galland B, Taylor R, Meredith-Jones K. ActiGraph GT3X+ and actical wrist and hip worn accelerometers for sleep and wake indices in young children using an automated algorithm: validation with polysomnography. Front Psych. 2020;10:958. 10.3389/fpsyt.2019.00958 PMC6970953 31992999

[151] Rosenberger ME, Buman MP, Haskell WL, McConnell MV, Carstensen LL. Twenty-four hours of sleep, sedentary behavior, and physical activity with nine wearable devices. Med Sci Sports Exerc. 2016;48(3):457–65. 26484953 10.1249/MSS.0000000000000778 PMC4760880

[152] Ancoli-Israel S, Cole R, Alessi C, Chambers M, Moorcroft W, Pollak CP. The role of actigraphy in the study of sleep and circadian rhythms. Sleep. 2003;26(3):342–92. 12749557 10.1093/sleep/26.3.342

[153] Johansson E, Ekelund U, Nero H, Marcus C, Hagstromer M. Calibration and cross-validation of a wrist-worn Actigraph in young preschoolers. Pediatr Obes. 2015;10(1):1–6. 24408275 10.1111/j.2047-6310.2013.00213.x

[154] Costa S, Barber SE, Cameron N, Clemes SA. Calibration and validation of the ActiGraph GT3X+ in 2–3 year olds. J Sci Med Sport. 2014;17(6):617–22. 24365695 10.1016/j.jsams.2013.11.005

[155] Ellis K, Kerr J, Godbole S, Lanckriet G, Wing D, Marshall S. A random forest classifier for the prediction of energy expenditure and type of physical activity from wrist and hip accelerometers. Physiol Meas. 2014;35(11):2191. 25340969 10.1088/0967-3334/35/11/2191 PMC4374571

[156] Staudenmayer J, He S, Hickey A, Sasaki J, Freedson P. Methods to estimate aspects of physical activity and sedentary behavior from high-frequency wrist accelerometer measurements. J Appl Physiol. 2015;119(4):396–403. 26112238 10.1152/japplphysiol.00026.2015 PMC4538283

[157] Addy CL, Trilk JL, Dowda M, Byun W, Pate RR. Assessing preschool children’s physical activity: how many days of accelerometry measurement. Pediatr Exerc Sci. 2014;26(1):103–9. 24092773 10.1123/pes.2013-0021

[158] Barreira TV, Schuna J, Tudor-Locke C, Chaput J-P, Church TS, Fogelholm M, et al. Reliability of accelerometer-determined physical activity and sedentary behavior in school-aged children: a 12-country study. Int J Obes Supplem. 2015;5(2):S29–35. 10.1038/ijosup.2015.16 PMC4850617 27152181

[159] Trost SG, Pate RR, Freedson PS, Sallis JF, Taylor WC. Using objective physical activity measures with youth: how many days of monitoring are needed? Med Sci Sports Exerc. 2000;32(2):426. 10694127 10.1097/00005768-200002000-00025

[160] Ward DS, Evenson KR, Vaughn A, Rodgers AB, Troiano RP. Accelerometer use in physical activity: best practices and research recommendations. Med Sci Sports Exerc. 2005;37(11 Suppl):S582–8. 16294121 10.1249/01.mss.0000185292.71933.91

[161] Tudor-Locke C, Barreira TV, Schuna JM, Mire EF, Chaput J-P, Fogelholm M, et al. Improving wear time compliance with a 24-hour waist-worn accelerometer protocol in the international study of childhood obesity, lifestyle and the environment (ISCOLE). Int J Behav Nutr Phys Act. 2015;12:1. 25881074 10.1186/s12966-015-0172-x PMC4328595

[162] Peeters G, van Gellecum Y, Ryde G, Farías NA, Brown WJ. Is the pain of activity log-books worth the gain in precision when distinguishing wear and non-wear time for tri-axial accelerometers? J Sci Med Sport. 2013;16(6):515–9. 23294696 10.1016/j.jsams.2012.12.002

[163] Choi L, Ward SC, Schnelle JF, Buchowski MS. Assessment of wear/nonwear time classification algorithms for triaxial accelerometer. Med Sci Sports Exerc. 2012;44(10):2009. 22525772 10.1249/MSS.0b013e318258cb36 PMC3443532

[164] Choi L, Liu Z, Matthews CE, Buchowski MS. Validation of accelerometer wear and nonwear time classification algorithm. Med Sci Sports Exerc. 2011;43(2):357–64. 20581716 10.1249/MSS.0b013e3181ed61a3 PMC3184184

[165] Trost SG, Loprinzi PD, Moore R, Pfeiffer KA. Comparison of accelerometer cut points for predicting activity intensity in youth. Med Sci Sports Exerc. 2011;43(7):1360–8. 21131873 10.1249/MSS.0b013e318206476e

[166] Moore R, Archer KR, Choi L. Statistical and machine learning models for classification of human wear and delivery days in accelerometry data. Sensors. 2021;21(8):2726. 33924388 10.3390/s21082726 PMC8069625

[167] Brønd JC, Arvidsson D. Sampling frequency affects the processing of Actigraph raw acceleration data to activity counts. J Appl Physiol. 2016;120(3):362–9. 26635347 10.1152/japplphysiol.00628.2015

[168] Banda JA, Haydel KF, Davila T, Desai M, Bryson S, Haskell WL, et al. Effects of varying epoch lengths, wear time algorithms, and activity cut-points on estimates of child sedentary behavior and physical activity from accelerometer data. PLoS ONE. 2016;11(3):e0150534. 26938240 10.1371/journal.pone.0150534 PMC4777377

[169] Aibar A, Bois J, Zaragoza J, Generelo E, Julián J, Paillard T. Do epoch lengths affect adolescents’ compliance with physical activity guidelines? J Sports Med Phys Fitness. 2014;54(3):326–34. 24739295

[170] Tudor-Locke C, Barreira TV, Schuna JM Jr, Mire EF, Katzmarzyk PT. Fully automated waist-worn accelerometer algorithm for detecting children’s sleep-period time separate from 24-h physical activity or sedentary behaviors. Appl Physiol Nutr Metab. 2014;39(1):53–7. 24383507 10.1139/apnm-2013-0173

[171] Toftager M, Kristensen PL, Oliver M, Duncan S, Christiansen LB, Boyle E, et al. Accelerometer data reduction in adolescents: effects on sample retention and bias. Int J Behav Nutr Phys Act. 2013;10(1):1–12. 24359480 10.1186/1479-5868-10-140 PMC3880051

[172] Pollak CP, Stokes PE, Wagner DR. Direct comparison of two widely used activity recorders. Sleep. 1998;21(2):207–12. 9542804 10.1093/sleep/21.2.207

[173] Zinkhan M, Berger K, Hense S, Nagel M, Obst A, Koch B, et al. Agreement of different methods for assessing sleep characteristics: a comparison of two actigraphs, wrist and hip placement, and self-report with polysomnography. Sleep Med. 2014;15(9):1107–14. 25018025 10.1016/j.sleep.2014.04.015

[174] Middelkoop H, Neven AK, Van Hilten J, Ruwhof C, Kamphuisen H. Wrist actigraphic assessment of sleep in 116 community based subjects suspected of obstructive sleep apnoea syndrome. Thorax. 1995;50(3):284–9. 7660344 10.1136/thx.50.3.284 PMC1021194

[175] Hjorth MF, Chaput J-P, Damsgaard CT, Dalskov S-M, Michaelsen KF, Tetens I, et al. Measure of sleep and physical activity by a single accelerometer: can a waist-worn Actigraph adequately measure sleep in children? Sleep Biol Rhythms. 2012;10:328–35.

[176] Montoye AH, Moore RW, Bowles HR, Korycinski R, Pfeiffer KA. Reporting accelerometer methods in physical activity intervention studies: a systematic review and recommendations for authors. Br J Sports Med. 2018;52(23):1507–16. 27539504 10.1136/bjsports-2015-095947

[177] Migueles JH, Rowlands AV, Huber F, Sabia S, van Hees VT. GGIR: a research community–driven open source R package for generating physical activity and sleep outcomes from multi-day raw accelerometer data. J Meas Phys Behav. 2019;2(3):188–96.

[178] Narayanan A, Desai F, Stewart T, Duncan S, Mackay L. Application of raw accelerometer data and machine-learning techniques to characterize human movement behavior: a systematic scoping review. J Phys Act Health. 2020;17(3):360–83. 32035416 10.1123/jpah.2019-0088

[179] Lee I-M, Moore CC, Evenson KR. Maximizing the utility and comparability of accelerometer data from large-scale epidemiologic studies. J Meas Phys Behav. 2023;6(1):6–12. 37206661 10.1123/jmpb.2022-0035 PMC10194828

[180] Rowlands AV, Dawkins NP, Maylor B, Edwardson CL, Fairclough SJ, Davies MJ, et al. Enhancing the value of accelerometer-assessed physical activity: meaningful visual comparisons of data-driven translational accelerometer metrics. Sports Med-open. 2019;5(1):47. 31808014 10.1186/s40798-019-0225-9 PMC6895365

[181] Full KM, Moran K, Carlson J, Godbole S, Natarajan L, Hipp A, et al. Latent profile analysis of accelerometer-measured sleep, physical activity, and sedentary time and differences in health characteristics in adult women. PLoS ONE. 2018;14(6):e0218595. 10.1371/journal.pone.0218595 PMC6597058 31247051

[182] ActivPAL. VANE algorithm PAL KNOWLEDGE BASE: ActivPAL; 2021 Available from: https://kb.palt.com/articles/vane/#:~:text=The%20VANE%20algorithm%20is%20the,sitting%2C%20standing%20and%20stepping%20events.

[183] Fruin ML, Rankin JW. Validity of a multi-sensor armband in estimating rest and exercise energy expenditure. Med Sci Sports Exerc. 2004;36(6):1063–9. 15179178 10.1249/01.mss.0000128144.91337.38

[184] Van Hees VT, Sabia S, Anderson KN, Denton SJ, Oliver J, Catt M, et al. A novel, open access method to assess sleep duration using a wrist-worn accelerometer. PLoS ONE. 2015;10(11):e0142533. 26569414 10.1371/journal.pone.0142533 PMC4646630

[185] van Hees VT, Sabia S, Jones SE, Wood AR, Anderson KN, Kivimäki M, et al. Estimating sleep parameters using an accelerometer without sleep diary. Sci Rep. 2018;8(1):12975. 30154500 10.1038/s41598-018-31266-z PMC6113241

[186] Tudor-Locke C, Barreira TV, Schuna JM Jr, Mire EF, Katzmarzyk PT. Fully automated waist-worn accelerometer algorithm for detecting children’s sleep-period time separate from 24-h physical activity or sedentary behaviors. Appl Physiol Nutr Metab. 2014;39(1):53–7. 24383507 10.1139/apnm-2013-0173

[187] Shin M, Swan P, Chow CM. The validity of Actiwatch2 and SenseWear armband compared against polysomnography at different ambient temperature conditions. Sleep Sci. 2015;8(1):9–15. 26483937 10.1016/j.slsci.2015.02.003 PMC4608890

[188] Meredith-Jones K, Williams S, Galland B, Kennedy G, Taylor R. 24 h accelerometry: impact of sleep-screening methods on estimates of sedentary behaviour and physical activity while awake. J Sports Sci. 2016;34(7):679–85. 26194337 10.1080/02640414.2015.1068438

[189] Barreira TV, Schuna JM Jr, Mire EF, Katzmarzyk PT, Chaput J-P, Leduc G, et al. Identifying children’s nocturnal sleep using 24-h waist accelerometry. Med Sci Sports Exerc. 2015;47(5):937–43. 25202840 10.1249/MSS.0000000000000486

[190] Oakley NR. Validation with polysomnography of the Sleepwatch sleep/wake scoring algorithm used by the Actiwatch activity monitoring system Mini Mitter Co. Sleep. 1997;2:1–140.

[191] ActivPAL. CREA Algorithm PAL Knowledge Base: ActivPal; 2021 Available from: https://kb.palt.com/articles/crea/.

[192] Esliger DW, Rowlands AV, Hurst TL, Catt M, Murray P, Eston RG. Validation of the GENEA accelerometer. Med Sci Sports Exerc. 2011;43(6):1085–93. 21088628 10.1249/MSS.0b013e31820513be

[193] Cabanas-Sánchez V, Higueras-Fresnillo S, De la Cámara MÁ, Veiga OL, Martinez-Gomez D. Automated algorithms for detecting sleep period time using a multi-sensor pattern-recognition activity monitor from 24 h free-living data in older adults. Physiol Meas. 2018;39(5):055002. 29667936 10.1088/1361-6579/aabf26

[194] Winkler EA, Bodicoat DH, Healy GN, Bakrania K, Yates T, Owen N, et al. Identifying adults’ valid waking wear time by automated estimation in activPAL data collected with a 24 h wear protocol. Physiol Meas. 2016;37(10):1653. 27652827 10.1088/0967-3334/37/10/1653

[195] Trost SG, Fees BS, Haar SJ, Murray AD, Crowe LK. Identification and validity of accelerometer cut-points for toddlers. Obesity. 2012;20(11):2317–9. 22173573 10.1038/oby.2011.364

[196] Ekblom O, Nyberg G, Bak EE, Ekelund U, Marcus C. Validity and comparability of a wrist-worn accelerometer in children. J Phys Act Health. 2012;9(3):389–93. 22454440

[197] Butte NF, Wong WW, Lee JS, Adolph AL, Puyau MR, Zakeri IF. Prediction of energy expenditure and physical activity in preschoolers. Med Sci Sports Exerc. 2014;46(6):1216–26. 24195866 10.1249/MSS.0000000000000209 PMC4010568

[198] Puyau MR, Adolph AL, Vohra FA, Zakeri I, Butte NF. Prediction of activity energy expenditure using accelerometers in children. Med Sci Sports Exerc. 2004;36(9):1625–31. 15354047

[199] Romanzini M, Petroski EL, Ohara D, Dourado AC, Reichert FF. Calibration of ActiGraph GT3X, Actical and RT3 accelerometers in adolescents. Eur J Sport Sci. 2014;14(1):91–9. 24533499 10.1080/17461391.2012.732614

[200] White T, Westgate K, Wareham NJ, Brage S. Estimation of physical activity energy expenditure during free-living from wrist accelerometry in UK adults. PLoS ONE. 2016;11(12):e0167472. 27936024 10.1371/journal.pone.0167472 PMC5147924

[201] Sasaki JE, John D, Freedson PS, Sasaki JE, John D, Freedson PS. Validation and comparison of ActiGraph activity monitors. J Sci Med Sport. 2011;14(5):411–6. 21616714 10.1016/j.jsams.2011.04.003

[202] Aguilar-Farías N, Brown WJ, Peeters GG. ActiGraph GT3X+ cut-points for identifying sedentary behaviour in older adults in free-living environments. J Sci Med Sport. 2014;17(3):293–9. 23932934 10.1016/j.jsams.2013.07.002

[203] Rowlands AV, Edwardson CL, Davies MJ, Khunti K, Harrington DM, Yates T. Beyond cut points: accelerometer metrics that capture the physical activity profile. Med Sci Sports Exerc. 2018;50(6):1323–32. 29360664 10.1249/MSS.0000000000001561

[204] Hager ER, Gormley CE, Latta LW, Treuth MS, Caulfield LE, Black MM. Toddler physical activity study: laboratory and community studies to evaluate accelerometer validity and correlates. BMC Public Health. 2016;16(1):1–10. 27600404 10.1186/s12889-016-3569-9 PMC5011903

[205] Janssen X, Cliff DP, Reilly JJ, Hinkley T, Jones RA, Batterham M, et al. Predictive validity and classification accuracy of ActiGraph energy expenditure equations and cut-points in young children. PLoS ONE. 2013;8(11):e79124. 24244433 10.1371/journal.pone.0079124 PMC3823763

[206] Pate RR, O’Neill JR, Mitchell J. Measurement of physical activity in preschool children. Med Sci Sports Exerc. 2010;42(3):508–12. 20068498 10.1249/MSS.0b013e3181cea116

[207] Adolph AL, Puyau MR, Vohra FA, Nicklas TA, Zakeri IF, Butte NF. Validation of uniaxial and triaxial accelerometers for the assessment of physical activity in preschool children. J Phys Act Health. 2012;9(7):944–53. 22207582 10.1123/jpah.9.7.944

[208] Chandler J, Brazendale K, Beets M, Mealing B. Classification of physical activity intensities using a wrist-worn accelerometer in 8–12-year-old children. Pediatr Obes. 2016;11(2):120–7. 25893950 10.1111/ijpo.12033

[209] Gavarry O, Bernard T, Giacomoni M, Seymat M, Euzet J, Falgairette G. Continuous heart rate monitoring over 1 week in teenagers aged 11–16 years. Eur J Appl Physiol. 1997;77:125–32. 10.1007/s004210050310 9459532

[210] Phillips LR, Parfitt G, Rowlands AV. Calibration of the GENEA accelerometer for assessment of physical activity intensity in children. J Sci Med Sport. 2013;16(2):124–8. 22770768 10.1016/j.jsams.2012.05.013

[211] Kozey-Keadle S, Libertine A, Lyden K, Staudenmayer J, Freedson PS. Validation of wearable monitors for assessing sedentary behavior. Med Sci Sports Exerc. 2011;43(8):1561–7. 21233777 10.1249/MSS.0b013e31820ce174

[212] Crouter SE, Flynn JI, Bassett DR Jr. Estimating physical activity in youth using a wrist accelerometer. Med Sci Sports Exerc. 2015;47(5):944–51. 25207928 10.1249/MSS.0000000000000502 PMC4362848

[213] Hurter L, Fairclough SJ, Knowles ZR, Porcellato LA, Cooper-Ryan AM, Boddy LM. Establishing raw acceleration thresholds to classify sedentary and stationary behaviour in children. Children. 2018;5(12):172. 30572683 10.3390/children5120172 PMC6306859

[214] Puyau MR, Adolph AL, Vohra FA, Butte NF. Validation and calibration of physical activity monitors in children. Obes Res. 2002;10(3):150–7. 11886937 10.1038/oby.2002.24

[215] Hamer M, Stamatakis E, Chastin S, Pearson N, Brown M, Gilbert E, et al. Feasibility of measuring sedentary time using data from a thigh-worn accelerometer: the 1970 British cohort study. Am J Epidemiol. 2020;189(9):963–71. 32219368 10.1093/aje/kwaa047 PMC7443760

[216] Heil DP. Predicting activity energy expenditure using the Actical^®^ activity monitor. Res Q Exerc Sport. 2006;77(1):64–80. 16646354 10.1080/02701367.2006.10599333

[217] Evenson KR, Wen F, Herring AH, Di C, LaMonte MJ, Tinker LF, et al. Calibrating physical activity intensity for hip-worn accelerometry in women age 60 to 91 years: the women’s health initiative OPACH calibration study. Prevent Med Rep. 2015;2:750–6. 10.1016/j.pmedr.2015.08.021 PMC4625400 26527313

[218] Vähä-Ypyä H, Vasankari T, Husu P, Mänttäri A, Vuorimaa T, Suni J, et al. Validation of cut-points for evaluating the intensity of physical activity with accelerometry-based mean amplitude deviation (MAD). PLoS ONE. 2015;10(8):e0134813. 26292225 10.1371/journal.pone.0134813 PMC4546343

[219] Copeland JL, Esliger DW. Accelerometer assessment of physical activity in active, healthy older adults. J Aging Phys Act. 2009;17(1):17–30. 19299836 10.1123/japa.17.1.17

[220] Matthews CE, Chen KY, Freedson PS, Buchowski MS, Beech BM, Pate RR, et al. Amount of time spent in sedentary behaviors in the United States, 2003–2004. Am J Epidemiol. 2008;167(7):875–81. 18303006 10.1093/aje/kwm390 PMC3527832

[221] Powell C, Carson BP, Dowd KP, Donnelly AE. Simultaneous validation of five activity monitors for use in adult populations. Scand J Med Sci Sports. 2017;27(12):1881–92. 27905148 10.1111/sms.12813

[222] Skotte J, Korshøj M, Kristiansen J, Hanisch C, Holtermann A. Detection of physical activity types using triaxial accelerometers. J Phys Act Health. 2014;11(1):76–84. 23249722 10.1123/jpah.2011-0347

[223] Troiano RP, Berrigan D, Dodd KW, Masse LC, Tilert T, McDowell M. Physical activity in the United States measured by accelerometer. Med Sci Sports Exerc. 2008;40(1):181. 18091006 10.1249/mss.0b013e31815a51b3

[224] Landry GJ, Falck RS, Beets MW, Liu-Ambrose T. Measuring physical activity in older adults: calibrating cut-points for the MotionWatch 8^©^. Front Aging Neurosci. 2015;7:165. 26379546 10.3389/fnagi.2015.00165 PMC4548198

[225] Lee P, Tse CY. Calibration of wrist-worn ActiWatch 2 and ActiGraph wGT3X for assessment of physical activity in young adults. Gait Posture. 2019;68:141–9. 30476691 10.1016/j.gaitpost.2018.11.023

[226] Reece JD, Barry V, Fuller DK, Caputo J. Validation of the sensewear armband as a measure of sedentary behavior and light activity. J Phys Act Health. 2015;12(9):1229–37. 25460142 10.1123/jpah.2014-0136

