# Supplementary material for: Methodological Choices on 24-h Movement Behavior Assessment by Accelerometry: A Scoping Review
Source: Sports Med Open. 2025 Mar 13;11:25. doi: 10.1186/s40798-025-00820-1 (PMC11906950; doi:10.1186/s40798-025-00820-1)
Supplement: Supplementary file 1 — Additional file1. [file 40798_2025_820_MOESM1_ESM.docx]

**Additional file 1 – Search Strategy**

**PubMed**

((("sedentary behavior"[Mesh] OR "sitting position"[Mesh] OR "screen time"[Mesh] or Sedent*[Title/Abstract]) AND (Sleep[Mesh] OR Sleep[Title/Abstract])) AND (Exercise[Mesh] OR Physical activity[Title/Abstract] OR Energy Expenditure[Title/Abstract] OR "health enhancing physical activity"[Title/Abstract] OR "active play"[Title/Abstract] OR "tummy time"[Title/Abstract] OR "prone position"[Title/Abstract] OR "floor time"[Title/Abstract]))) OR ("Movement Behavi*"[Title/Abstract])

AND

(acceleromet*[Title/Abstract] OR wearable[Title/Abstract] OR "activity monitor"[Title/Abstract] OR actigraph*[Title/Abstract] OR actical[Title/Abstract] OR RT3[Title/Abstract] OR tritrac[Title/Abstract] OR armband[Title/Abstract] OR activpal[Title/Abstract] OR sensewear[Title/Abstract] OR biotrainer[Title/Abstract] OR dynapor[Title/Abstract] OR caltrac[Title/Abstract] OR actiheart[Title/Abstract] OR tracmoor[Title/Abstract] OR genea[Title/Abstract] OR geneactive[Title/Abstract] OR Minilogger[Title/Abstract] OR actiwatch[Title/Abstract] OR actitrac[Title/Abstract] OR hookie[Title/Abstract])

**PsycInfo and SportDiscus**

("exp sedentary".mp. OR "sedentary behavi*r" OR sedentary OR "sedentariness" OR sedentarism OR "sedentary lifestyle" OR "sitting" OR "inactiv*" OR "physical* inactiv*" OR "low energy expenditure") AND ("exp sleep".mp. OR sleep* OR "sleep time" OR bedtime OR bedtime* OR "wake up time" OR "exp sleep duration".mp. OR sleepiness OR "time in bed" OR "sleep hour*" OR "sleep quantity" OR "insufficient sleep") AND (exp Exercise OR "Energy Expenditure" OR "physical activit*" OR walking OR training OR run* OR swim* OR "active commut*" OR "active behavi?r" OR jog* OR bicycl* OR cycle OR "physical exertion" OR "health enhancing physical activity" OR "recreational activit*" OR "active play".mp. OR "tummy time".mp. OR "prone position".mp. OR "floor time".mp.) OR (Movement Behavio*.mp.)

AND

(acceleromet*.mp. OR wearable.mp. OR "activity monitor".mp. OR actigraph*.mp. OR actical.mp. OR RT3.mp. OR tritrac.mp. OR armband.mp. OR activpal.mp. OR sensewear.mp. OR biotrainer.mp. OR dynapor.mp. OR caltrac.mp. OR actiheart.mp. OR tracmoor.mp. OR genea.mp. OR geneactive.mp. OR Minilogger.mp. OR actiwatch.mp. OR actitrac.mp. OR hookie.mp.)

**EMBASE**

('exp sedentary':ab,ti OR 'sedentary behavi*r':ab,ti OR sedentary:ab,ti OR sedentariness:ab,ti OR sedentarism:ab,ti OR 'sedentary lifestyle':ab,ti OR sitting:ab,ti OR inactiv*:ab,ti OR 'physical* inactiv*':ab,ti OR 'low energy expenditure':ab,ti) AND ('exp sleep':ab,ti OR sleep*:ab,ti OR 'sleep time':ab,ti OR bedtime:ab,ti OR bedtime*:ab,ti OR 'wake up time':ab,ti OR 'sleep duration':ab,ti OR 'time in bed':ab,ti OR 'sleep hour*':ab,ti OR 'sleep quantity':ab,ti OR 'insufficient sleep':ab,ti) AND (('exp exercise':ab,ti OR 'energy expenditure':ab,ti OR 'physical activit*':ab,ti OR walking:ab,ti OR training:ab,ti OR run*:ab,ti OR swim*:ab,ti OR 'active commut*':ab,ti OR 'active behavi?r':ab,ti OR jog*:ab,ti OR bicycl*:ab,ti OR cycle:ab,ti OR 'physical exertion':ab,ti OR 'health enhancing physical activity':ab,ti OR 'recreational activit*':ab,ti OR 'active play':ab,ti OR 'tummy time':ab,ti OR 'prone position':ab,ti OR 'floor time':ab,ti) OR ('movement behavio*':ab,ti)

AND

('exp acceleromet*':ab,ti OR wearable:ab,ti OR 'activity monitor':ab,ti OR actigraph*:ab,ti OR actica:ab,ti OR RT3:ab,ti OR tritrac:ab,ti OR armband:ab,ti OR activpal:ab,ti OR sensewear:ab,ti OR biotrainer:ab,ti OR dynapor:ab,ti OR caltrac:ab,ti OR actiheart:ab,ti OR tracmoor:ab,ti OR genea:ab,ti OR geneactive:ab,ti OR Minilogger:ab,ti OR actiwatch:ab,ti OR actitrac OR hookie:ab,ti)
