# Supplementary material for: Methodological Choices on 24-h Movement Behavior Assessment by Accelerometry: A Scoping Review
Source: Sports Med Open. 2025 Mar 13;11:25. doi: 10.1186/s40798-025-00820-1 (PMC11906950; doi:10.1186/s40798-025-00820-1)
Supplement: Supplementary file 2 — Additional file2. [file 40798_2025_820_MOESM2_ESM.docx]

**Additional file 1 – Individual results**

Table 1. Characteristics of individual reports

| Authors and year | Country; Study | Type of study | N | Age | Sex (% of girls/woman) |
| --- | --- | --- | --- | --- | --- |
| **Toddlers (2-3 years)** | | | | | |
| Armstrong et al. 2019^1^ | USA; N.R. | Cross-sectional | 101 | 12 to 32 months | 39% |
| Santos et al., 2017^2^ | Australia; GetUp study) | Cross-sectional | 202 | 12 to 28 months | 51.5% |
| Haines et al. 2018^3^ | Canada; The Guelph Family Health Study) | RCT | 42 | 18 to 60 months | N.R. |
| **Preschoolers (3-5 years)** | | | | | |
| Alonso-Martínez et al. 2021^4^ | Spain; N.R. | Cross-sectional | 268 | 4 to 6 years | 46.6% |
| Chang et al. 2021^5^ | China; N.R. | Cross-sectional | 458 | 3 to 6 years | 50.6% |
| Chen et al., 2020^6^ | Singapore; Growing Up in Singapore Towards healthy Outcomes (GUSTO) | Cross-sectional | 552 | 5 years | 48% |
| Craemer et al., 2021^7^ | Belgium; N.R. | Cross-sectional | 16 | 3 to 5 years | 50% |
| Duraccio et al., 2017^8^ | USA; N.R. | Cross-sectional | 131 | 4 to 6 years | 43.5% |
| Ng et al., 2021^9^ | China; SUNRISE | Cross-sectional | 25 | 4.4 (0.3) years | N.R. |
| Tomaz et al., 2019^10^ | South Africa; N.R. | Cross-sectional | 78 | 3 to 5 years | 50% |
| Meredith-Jones et al., 2019^11^ | New Zealand; Prevention of Overweight in Infancy (POI) study | Longitudinal | 547 | 1, 2 and 5 years | N.R. |
| Laurent et al., 2020^12^ | USA; N.R. | Longitudinal | 288 | 3 to 5 years | 47.2% |
| Williams et al., 2014^13^ | New Zealand; FLAME - Family Lifestyle, Activity, Movement and Eating | Cross-sectional | 234 | 3 to 7 years | 42.74% |
| Butte et al., 2016^14^ | China; N.R | Cross-sectional | 254 | ≥ 4.0 years [4.1 (6.3) years] | 46.9% |
| Hossain et al. 2021^15^ | Bangladesh; SUNRISE study | Cross-sectional | 65 | 3 to 5 years [4.5 (0.3)] | 47.8% |
| Kang et al. 2021^16^ | USA; N.R. | Cross-sectional | 30 | 3 to 5 years [3.8 (1.0)] | 43.0% |
| Kuzik et al. 2020^17^ | Canada; The Parent-Child Movement Behaviours and Pre-School Children’s Development study | Cross-sectional | 95 | 3 to 5 years [4.5 (0.07)] | 30.5% |
| Guan et al. 2020^18^ | USA; N.R. | Longitudinal | 111 | 3 to 5 years | 47.75% |
| **Children (5-10 years)** | | | | | |
| Manyanga et al., 2019^19^ | Australia, Brazil, Canada, China, Colombia, Finland,India, Kenya, Portugal, South Africa, the UK and USA; International Study of Childhood Obesity, Lifestyle and the Environment (ISCOLE) | Cross-sectional | 8,055 | 9–11 years | 54.7% |
| Ekstedt et al., 2013^20^ | Sweden; The Stockholm Obesity Prevention Project (STOPP) | Cross-sectional | 1,231 | 6-10 years | N.R. |
| Padmapriya et al., 2021^21^ | Singapore; GUSTO study | Longitudinal | 634 | 5.5 and 8 years | 48.4% |
| Taylor et al., 2020^22^ | New Zealand; N.R. | Cross-sectional | 690 | 6 to 10 years | 51.5% |
| Vuholm et al., 2021^23^ | Denmark; Fish, children, health, and cognition (FiSK) | RCT | 194 | 8 to 9 years | 50% |
| Burkart et al., 2021^24^ | USA; N.R. | Quasi-experimental | 231 | 7 to 12 years | 47% |
| Haszard et al., 2020^25^ | New Zealand; PLAY study | Cross-sectional | 742 | 8.0 (1.1) | 51.4% |
| Toledo-Vargas et al., 2020^26^ | Chile; N.R. | Cross-sectional | 258 | 9 to 11 years | 50.5% |
| Armstrong et al., 2021^27^ | USA; N.R. | Cross-sectional | 196 | 5 to 10 years | 53% |
| Fairclough et al., 2017^28^ | UK; Active Schools: Skelmersdale (AS:Sk) study | Cross-sectional | 169 | 9 to 10 years | 50.3% |
| Hjorth et al., 2016^29^ | (Denmark; OPUS Optimal well-being, development, and health for Danish children through a healthy New Nordic Diet School Meal Study | Cross-sectional | 828 | 8 to 11 years | 49% |
| Moreno et al., 2021^30^ | USA; N.R. | Longitudinal | 119 | 5 to 8 years | 57% |
| Antczak et al., 2021^31^ | Australia; Internet-based professional Learning to help teachers support Activity in Youth” (iPLAY) | Longitudinal | 1059 | 8 to 9 years | 50% |
| García-Alonso et al., 2022^32^ | Spain; Observatorio de Actividad Física en escolares de 3 a 9 años | Longitudinal | 110 | 4 to 7 years | 50.91% |
| Hedayatrad et al. 2022^33^ | New Zealand; Growing Up in New Zealand study (GUiNZ) | Cross-sectional | 623 | 8.0 years [7.8 (0.2)] | 51.5% |
| Lucas-de la Cruz et al. 2018^34^ | Spain; Prevention of excess weight in schoolchildren (MOVI-2) | Cross-sectional | 146 | 8 to 11 years [9.4 (0.7)] | 54.8% |
| Martinez et al. 2019^35^ | USA; N.R. | Longitudinal | 308 | 8 to 10 years [8.9 (0.8)] | 53.0% |
| **Adolescents (10-17 years)** | | | | | |
| Caetano et al. 2021^36^ | Brazil; N.R. | Cross-sectional | 309 | 14 to 16 years | 57% |
| Chong et al. 2021^37^ | Australia; N.R. | Longitudinal | 88 | 10 to 13 years | 59.1% |
| Costa et al., 2020^38^ | Brazil; Estudo Longitudinal do Estilo de Vida de Adolescentes’ (ELEVA) | Cross-sectional | 688 | 14–18 years | 50.4% |
| Dumuid et al., 2021^39^ | Australia; Child Health CheckPoint data and Longitudinal Study of Australian Children (LSAC) | Cross-sectional | 1,182 | 11 to 12 | 49% |
| Negele et al., 2020^40^ | Germany; GINIplus and LISA studies | Longitudinal | 1223 | 15 years | 55% |
| Tye et al; 2020 | Tye et al., 2020 New Zealand; N.R. | Cross-sectional | 119 girls | 15 to 18 years | NA |
| Van Dyk et al., 2018^41^ | USA; N.R. | Randomized intervention without control | 18 | 14 to 17 years | 66.7% |
| Fairclough et al., 2021^41^ | UK; N.R. | Cross-sectional | 359 | 9 to 13 years | 50.7% |
| Collings et al., 2015^42^ | UK; the ROOTS study | Cross-sectional | 144 | 15 to 17.5 years | 50% |
| Gába et al., 2020^43^ | Czech Republic; N.R. | Cross-sectional | 659 | 8 to 18 years | 58% |
| Talarico et al., 2018^44^ | Canada; N.R. | Cross-sectional | 434 | 10 to 13 years | 50.2% |
| Starbek et al., 2022^45^ | Slovenia; N.R. | Cross-sectional | 51 | 15 to 19 years | 51% |
| Grant et al. 2020^46^ | USA; N.R. | Cross-Sectional | 36 | N.R (6th to 8th grades) | 58.3% |
| Krietsch et al. 2022^47^ | USA; N.R. | Longitudinal | 90 | 14-17 years  [15.7 (1.1)] | 67.0% |
| Master et al. 2017^48^ | USA: The Fragile Families & Child Wellbeing Study (FFCWS) | Cross-sectional | 417 | 15 years  [15.5 (0.6)] | 53.0% |
| Merikanto et al. 2020^49^ | Finland; The Genome-wide association study (GWAS) | Longitudinal | 353 | 12 years  [12.3 (0.5)] | 53.0% |
| Beltran-Valls et al., 2021^50^ | Spain; DADOS (Deporte, ADOlescencia y Salud) | Longitudinal | 189 | 13.9 (0.3) years | 48.2% |
| Domingues et al., 2022^51^ | Brazil; N.R. | Cross-sectional | 185 | 15 to 18 years | 49.2% |
| Harrington et al., 2021^52^ | UK; N.R.) | Cross-sectional | 816 girls | 11 to 14 years | NA |
| Aguilar-Farias et al., 2020^53^ | Chile; ESPACIOS | Cross-sectional | 148 | 9 to 11 years | 52.7% |
| Butte et al., 2007^54^ | USA; VIVA LA FAMILIA Study | Cross-sectional | 897 | 4 to 19 years | 50.8% |
| Kim et al., 2020^55^ | USA; N.R. | Cross-sectional | 263 | 12.24 (0.99) years | 48.7% |
| **Adults and older adults (≥ 18 years)** | | | | | |
| Ataka et al. 2022^56^ | Japan; N.R. | Cross-sectional | 56 | 74.2 (3.9) | 58.9% |
| Betson et al. 2022^57^ | Australia; N.R. | Cross-sectional | 28 | 25.5 (4.5) | 53.6% |
| Blodgett et al. 2022^58^ | UK; 1970 British Cohort Study | Cross-sectional | 6,562 | 45 yrs | N.R. |
| Booth et al. 2012^59^ | USA; N.R. | Cross-sectional | 48 | 21 to 40 | 56.3% |
| Buman et al. 2016^60^ | USA; N.R. | Longitudinal | 20 | 35 to 65 | 15% |
| Cabanas-Sánchez et al. 2019^61^ | Spain; Seniors-ENRICA-2 study | Cross-sectional | 3273 | 65 to 94 years | 53.1% |
| Carneiro-Barrera et al. 2020^62^ | Spain; N.R. | Cross-sectional | 187 | 18-25 years | 64.7% |
| Dumuid et al., 2018^63^ | Australia; N.R. | Cross-sectional | 122 | 60 to 70 | 61% |
| Ellingson et al., 2018^64^ | USA; N.R. | Cross-sectional | 271 | 21 to 35 years | 48.7% |
| Full et al., 2020^65^ | USA; Women’s Health Initiative OPACH Study | Cross-sectional | 3,329 women | 63 to 99 years | NA |
| Liao et al., 2020^66^ | USA; N.R. | Longitudinal | 105 | 23 to 68 years | 95% |
| Millard et al., 2021^67^ | UK; UK BIOBANK | Longitudinal | 79,503 | 40 to 69 years | 54.5% |
| Mochón‑Benguigui et al., 2021^68^ | Spain; FIT-AGEING study« | Cross-sectional | 74 | 40–65 years | 52.7% |
| Park et al., 2022^69^ | USA; N.R. | Cross-sectional | 75 women | 18 to 75 years | NA |
| Suorsa et al., 2022^70^ | USA; Physical Activity Measurement Survey (PAMS) | Cross-sectional | 1,235 | 21 to 70 | 56.6% |
| Curtis et al., 2020^71^ | Finland; Finnish Retirement and Aging Study (FIREA) | Longitudinal | 551 | 63.3 (1.1) years | 86% |
| Cabanas-Sanchez et al., 2018^72^ | Australia; Active Team | Cross Sectional | 430 | 18 to 65 years | 74% |
| Husu et al., 2021^73^ | Spain; IMPACT65+ | Cross Sectional | 607 | 65 to 92 years | 65.7% |
| Knaeps et al., 2017^74^ | Finland; FinFit 2017 study | Cross Sectional | 2,256 | 20 to 69 years | 59% |
| Mitchell et al., 2016^75^ | Belgium; N.R. | Cross Sectional | 410 | 29 to 82 years | 36% |
| Powell et al., 2020^76^ | USA; Nurses Health Study II | Cross Sectional | 353 women | 21 to 75 years | N/A |
| Gupta et al., 2020^77^ | (Denmark; Physical wOrk Demands and Prospective register-based Sickness Absence study (PODESA) cohort; ‘Danish Physical ACTivity cohort with Objective measurements’ (DPhacto); ‘New method for Objective Measurements of physical Activity in Daily living’ (NOMAD) cohort.) | Longitudinal | 929 | 44.9 (9.7) | 45% |
| Verhoog et al., 2020^78^ | Netherlands; Rotterdam Study | Cross-Sectional | 1,954 | 51 to 94 years | 51.5% |
| Full et al., 2019^79^ | USA; Transdisciplinary Research in Energetics and Cancer (TREC) | Cross-Sectional | 372 women | 21–75 years  [55.38 (10.16)] | N/A |
| Galmes-Panades et al. 2019^80^ | Spain; The PREDIMED-Plus study | Cross-Sectional | 2189 | 55–75 years  [65.0 (4.95)] | 47.1% |
| German et al. 2020^81^ | USA; Multi-Ethnic Study on Atherosclerosis [MESA] Sleep Ancillary Study) | Cross-Sectional | 1718 | 45-84 years  [68 (n.r.)] | 54.0% |
| Gilson et al. 2021^82^ | Australia; Truck-Fit study | RCT | 37 men | Age range N.R.  45.9 (10.2) | N/A |
| Goncin et al. 2020^83^ | Canada; N.R. | Longitudinal | 28 | > 60 years  [69.7 (6.5)] | 50.0% |
| Gubelmann et al. 2018^84^ | Switzerland; CoLaus study | Cross-sectional | 2649 | 45–86 years [61.6 (9.8)] | 53.5% |
| Gupta et al. 2018^85^ | Denmark; DPHACTO cohort | Cross-sectional | 827 | ≥ 18 years  [45 (10) years] | 46.2% |
| Hargens et al. 2021^86^ | USA; N.R. | Cross-sectional | 81 | N.R.  [20.2 (1.5)] | 65.0% |
| Heiland et al. 2021^87^ | Sweden; Physical Activity and Healthy Brain Functions | Cross-sectional | 324 | ≥ 18 years  [42.4 (9.0)] | 62.7% |
| Imes et al. 2021^88^ | USA; EMPOWER Study | Cross-sectional | 108 | ≥ 18 years  [51.2 (10.6)] | 88.9% |
| Le et al. 2021^89^ | (Australia; ACES, DESTRESS, and SHS) | Cross-Sectional | 361 | ≥ 18 years  [22.6 (5.3)] | 72.6% |
| Lee et al. 2020^90^ | USA; Healthy Women Study | Cross-Sectional | 136 women | 42-50 years  [73.3 (1.7)] | N.A. |
| Madden et al. 2014^91^ | Canada; N.R. | Cross-Sectional | 51 | ≥ 65 years  [71.5 (0.6)] | 52.9% |
| McDonough et al. 2022^92^ | USA; N.R. | RCT | 96 | 18 - 35 years  [22.9 (3.7)] | 75.0% |
| Meyer et al. 2020^93^ | USA; The Energy Balance Study | Longitudinal | 348 | 20-35 years  [27.7 (3.7)] | 50.0% |
| Pasanen et al. 2022^94^ | Finland; REACT | RCT | 345 | N.R.  [65.2 (1.1)] | 82.3% |
| Seol et al., 2019^95^ | Japan; N.R. | Cross-Sectional | 70 | 65 to 84 years | 77.1% |
| Tigbe et al., 2017^96^ | UK; N.R. | Cross-Sectional | 111 | 22 to 60 years | 13.5% |
| Wang et al., 2022^97^ | Sweeden; N.R. | Single-blind, parallel-group, randomized trial | 245 | 18 to 70 years | 73.1% |
| Santos et al., 2021^98^ | Brazil; N.R. | Longitudinal | 27 | At least 15 | 60% |

**Table 2. Results of individual reports**

| ID - Authors and year (Country/ study) | Acc model, Placement on the body; software used | Nº of days for data collection; nº of required valid days (minutes required) | Criteria for non-wear time | Epoch Length and data reduction (epoch integration) | Movement Behavior outcome and Reporting formats | Sleep measuring procedure and PA and SB cut points | Procedures handling non-compliant participants | Log or diary |  |
| --- | --- | --- | --- | --- | --- | --- | --- | --- | --- |
| **Toddlers (2-3 years)** | | | | | | | | | |
| Armstrong et al. 2019^1^ | Actical on non-dominant ankle, nonremovable; Actical software. | 3-7 days; ≥ 3 days with a daily average of 80 counts per minute, including 1 weekend day (24 h). | N/A | 1-min epoch. | Sleep duration (Hours/day).  SB, LPA, MPA, VPA and TPA (min/day). | Sleep duration (Total sleep time: number of minutes asleep between sleep onset and sleep offset.; Daytime napping: 30 successive epochs of sleep that occurred between 09:00 and 17:00.); Sleep algorithm (Sadeh et al., 1994)^99^, assisted by log.  SB (0-40 cpm), LPA (41 2200 cpm), MVPA (≥ 2201 cpm); Hager et al. 2016^100^. | Participants who responded to 5 or fewer survey prompts with corresponding Actical data were excluded. | Ecological momentary assessment for sleep. |  |
| Santos et al., 2017^2^ | Actigraph GT3X+ on right hip; ActiLife. | 7 days; ≥ 1 24h period. | Through manually and visually  Screened. | Sampled at 30 Hz and reintegrated into 15-s epoch. | Sleep, SB, LPA, MVPA, TPA (min/day). | Sleep duration (including nighttime and daytime naps) was manually and visually screened, considering log and accelerometer files.  SB (< 25 cp15s); LPA (25–420 cp15s) and MVPA (>420 cp15s); Trost et al., 2012^101^. | Non-compliant participants were excluded from the analyses. | Sleep and non-wear time diary. |  |
| Haines et al. 2018^3^ | ActiGraph GT3X on non-dominant wrist; Actilife | 7 days (except water-based activities); ≥3 days (N.R.) | Choi’s algorithm^102^ | 1-sec epochs | Sleep, SB, LPA, and MVPA (min/day) | Sleep (duration) defined by algorithm Sadeh and Tudor-Locke algorithms.  SB, LPA, and MVPA (N.R.).  < 3 years - Trost et al. 2012^101^  ≥ 3 years - Butte et al. 2014^103^ | Non-compliant participants were excluded from the analyses. | No use. |  |
| **Preschoolers (3-5 years)** | | | | | | | | | |
| Alonso-Martínez et al. 2021^4^ | GENEActiv tri-axial on non-dominant  wrist; GGIR package in R | 6 days; 6 days (≥600 min during awake time and an average sleep time ≥200 min) | N.R. | Raw data were sampled at 87.5 Hz and then reintegrated into 1-s epochs | Sleep duration (Hours/day)  SB, LPA, MPA, VPA and TPA (min/day) | Sleep (sleep onset to offset) defined by the algorithm Hees et al. 2015^104^.  SB, LPA, MPA and VPA, TPA (total recorded counts/wear time) ^105^ | Non-compliant participants were excluded from the analyses. | No use. |  |
| Chang et al. 2021^5^ | ActiGraph GT3X-BT on right hip; ActiLife | 7 days except in water activities; 4 days including 1 weekend day (≧1,000 min/day) | Choi algorithm | 15-s epoch | Sleep, SB, LPA, MVPA, TPA (min/day) | Sleep (Sleep duration: Visual diagnosis by experts per minute) defined by algorithm Tudor-Locke (2014)^106^, and mode of low frequency extension: SB (0–467 cp15s), LPA (468–2,207 cp15s); MPA (2,208–3,991 cp15s) and VPA (≥3,992 cp15s); The origin of these cut-off points is not clear. | Non-compliant participants were excluded from the analyses. | No use. |  |
| Chen et al., 2020^6^ | ActiGraph wGT3X-BT on non-dominant wrist, nonremovable; Actilife | 7 days, 2 weekdays and 1 weekend day (16h/day) | Non-wear time was estimated based on the standard deviation and value range of each accelerometer axis, using a 60-min window with 15-min increments. | 80 Hz | Sleep, SB, LPA and MVPA (h/day) | Sleep (Sleep duration) defined by the algorithm (Hees et al., 2015)^104^.  SB (≤35 mg), LPA (35–200 mg and MVPA (>200 mg); ENMO values were averaged over 5 s epochs, Hildebrand et al., 2014^107^ and 2017^108^. | The invalid data were imputed using the mean value of valid data at same time points on other days | No use. |  |
| Craemer et al., 2021^7^ | ActivPAL on right anterior thigh midway and the Axivity on non-dominant wrist; ActivPAL; Open Movement software | 7 days, except in water activities (just for Axivity; N.R. (N.R.) | N.R. | 1-min epoch | Sleep, SB, TPA (% of 24h) | Sleep (Time in bed: start time and end time) defined by the algorithm.  SB (If more than 45 s  of the 60 s epoch were spent sitting or lying, this epoch was classified as SB)  Activity (Yes if there was no SB and no Sleep) (VANE algorithm) | Non-compliant participants were excluded from the analyses. | No use. |  |
| Duraccio et al., 2017^8^ | Actigraph GT3X+ on right hip; ActiLife and MeterPlus4. | 3 days; N.R. (≥ 8 h/day of PA. | If no movement was recorded during the night, it was assumed that the accelerometer was removed prior to bedtime.  60 consecutive min of nonmovement was defined as an invalid hour. | 15-s epoch. | Sleep (h/day).  SB, MPA and VPA (min/day). | Sleep (Sleep duration) defined by algorithm (Sadeh et al., 1994) ^99^.  SB (0-199 cp15s), MPA (420-841 cp15s) and VPA (≥842 cp15s); Pate et al., 2006^109^. | Non-compliant participants were excluded from the analyses. | Parents reported their child's typical bedtime and wake time during the week on the demographics survey. |  |
| Ng et al., 2021^9^ | ActiGraph wGT3X-BT on right hip | 5 days, except in water activities; 2 days (all sleep and 8 h/day) | 20 min or more of consecutive zeros | Sampled at 30 Hz which was integrated into 15 s epochs. | Sleep, SB, LPA, MVPA (min/day). | Sleep (determined through visual inspection of the acceleration graphs)  SB (≤199), LPA (≥200 to 419), MPA (≥420 to 841 cp15s) and VPA (≥842 cp15s); Pate et al., 2006 ^109^. | Non-compliant participants were excluded from the analyses. | Parents reported sleep timings sedentary screen time, total PA, MVPA, and the time children spent outdoors at both time points using questionnaires. |  |
| Tomaz et al., 2019^10^ | Actigraph GT3X+ on right hip; Actilife | 7 days, except in water activities; ≥3 weekdays and ≥1 weekend day (≥ 7 waking hours for PA; (>160 min for sleep) | 20 minutes of consecutive  zeroes and was excluded from daytime and nocturnal analyses | Sampled at 80 Hz and converted to 15-s epoch for PA and 1-min epoch for sleep | Sleep (h/day).  SB, LPA, MPA, VPA (min/day). | Sleep (nap times were  identified during the daytime by visual inspection; Nocturnal sleep: by visual inspection); (Chow et al., 2016)^110^  SB (≤25 cp15s), LPA (>25 cp15s) and MPA (>420 cp15s); Janssen et al., 2003^111^ and VPA (>842 cp15s); Pate et al., 2010^112^ | Non-compliant participants were excluded from the analyses. | Naps daily schedules  of the preschools were used.  Sleep diary was not used. |  |
| Meredith-Jones et al., 2019^11^ | Actical on hip; MATLAB | 7 days; ≥3 days (≥8 waking hours) | At least  20 min of consecutive zeros (Esliger et al., 2005)^113^. | 15-s epochs. | Sleep, SB, TPA (min/day). | Sleep (sleep onset, as the start of the first 15 continuous minutes of sleep preceded by 5 min of awake, to offset, as the last of 15 continuous minutes of sleep followed by 5 min of awake. Matlab script defines naps during daytime wake periods (9 a.m.–5 p.m.) as at least 30 min of continuous sleep, preceded by 5 min of awake).  SB (0–6 counts cp15s), LPA (7–286 cp15s), MVPA (≥287 cp15s); There is no cut points for this age range, but the presented ones were based on Adolph et al., 2012^114^ and Trost et al., 2010^101^ | Non-compliant participants were excluded from the analyses. | No use. |  |
| Laurent et al., 2020^12^ | Actiwatch Spectrum on non-dominant wrist; Actiware software | 16 days; N.R. (≥480 min of wear time and full night) | Off-wrist detection with a button  to mark events in the record | Sampled at 32 Hz and converted in 15-s epoch | Sleep, SB, LPA, MVPA (min/day) | Sleep (nap, nighttime and total sleep duration) was defined by combination of sleep diaries and button-marked events. If sleep diary entries or event  markers were unavailable, the first three consecutive minutes of sleep were used to define sleep onset, and the last five consecutive minutes of sleep defined sleep offset for sleep episodes.  SB (≤ 79 cp15s), LPA (80 to 261 cp15s), MVPA (≥ 262 cp15s); Ekblom et al., 2012^115^ | Non-compliant participants were excluded from the analyses. | Sleep diary. |  |
| Williams et al., 2014^13^ | Actical on hip; Actilife. | 5 days including 2 weekend days (N.R.) | at least 20 consecutive minutes of zero counts (Cliff et al., 2009)^116^. | 15-s epoch. | Sleep, SB, LPA, MVPA (h/day) | Sleep (duration) was defined by algorithm (Sadeh et al., 1994) ^99^.  SB (0–47 cpm), LPA (48–2031 cpm), MPA (2032–2875 cpm), VPA (≥ 2876); Evenson et al., 2008^117^. | Data were excluded if the total counts for the day were less than  10,000 or more than 20 million total counts | Sleep diary completed by parents. |  |
| Butte et al., 2016^14^ | ActiGraph GT3X on right hip and Actiheart on chest; N.R. | 7 days except in water activities; 4 days including 1 weekend day (≥ 1000 min/day) | 20 min  or more of consecutive zero counts, if the interval was not identified as nighttime sleep, nap time, or device removal for bathing or aquatic activities in the records completed by the parents. | 1-min epoch | Sleep, SB, MVPA (min/day) | Sleep (Sleep onset was identified by inactivity (counts usually zero) and a gradual decline in heart rate. Sleep termination was identified by abrupt increases in activity and heart rate. This was supported with visually screening)  SB (<821 cpm) and MVPA (>3909); Butte et al., 2014^103^ | Non-compliant participants were excluded from the analyses. | Sleep and non-wear diary. |  |
| Hossain et al. 2021^15^ | ActiGraph wGT3X-BT on the right hip and activPAL4 on the right thigh; Actilife | 4 days, except water-based activities; 1 day (≥6 waking h/day) | ≥ 20 min of consecutive zero counts and by  visual inspection of the ActiGraph data | Sampled at 30 Hz and integrated into 15-s epochs | Sleep, SB, LPA, and MPA (min/day) | Sleep (Manually and visually screened, considering log and accelerometer files)  SB (< 800 cpm), LPA (800–1679 cpm), MPA (1680–3367 cpm) and VPA (≥3368 cpm; Pate et al., 2006^109^ | Non-compliant participants were excluded from the analyses. | Parents reported sleep timings, screen time, total PA, MVPA, and the time children spent outdoors at both time points using questionnaires |  |
| Kang et al. 2021^16^ | ActiGraph GT3X+ on hip; Actilife | 5 days (24h), except water-based activities; 5 days including 2 weekend days and 3 weekdays (10 waking h/day) | ≥ 20 min of consecutive zero counts outside the hours of bedtime and waketime | Sampled at 30 Hz and integrated into 5-s epochs | Sleep, SB, LPA, and MPA (min/day) | Sleep (duration) defined by  Algorithm (Hjorth et al. 2012)^118^  SB, LPA, and MVPA (N.R.)  Freedson et al.1998^119^ | Non-compliant participants were excluded from the analyses. | Sleep diary. |  |
| Kuzik et al. 2020^17^ | ActiGraph  wGT3X-BT on the right hip; R software | 7 days (24h), except water-based activities; ≥3 days (≥10 waking h/day) | ≥ 20 min of consecutive zero counts outside the hours of bedtime and waketime | Sampled at 30 Hz and integrated into 5-s epochs | Sleep, SB, LPA, and MPA (min/day) | Sleep (duration) defined by  Algorithm (Hjorth et al. 2012)^118^.  SB (≤25 cp15s), LPA (26–419 cp15s) and  MVPA (≥420 cp15s);  Hjorth et al. 2012^118^ | Non-compliant participants were excluded from the analyses. | sleep and accelerometer wear time log. |  |
| Guan et al. 2020^18^ | ActiGraph GT9X on hip (24h); Actilife | 3 days (except water-based activities); 1 day (24h) | 20 min of consecutive zero counts | 15-s epochs | Sleep, SB, Screen time, LPA, MPA and VPA (min/day) | Sleep (at least 10 consecutive minutes with a vector magnitude > 0).  SB (≤100 cpm), LPA (101–799 cpm), MPA (800–1679 cpm), and MVPA (≥1680 cpm).  Pate et al. 2006^109^ | Those with <24h period were excluded | Activity log (including nap(s), bedtime, wake-up time, and accelerometer non-wear time); Screen time parent-report questionnaire |  |
| **Children (5-10 years)** | | | | | | | | | |
| Manyanga et al., 2019^19^ | ActiGraph GT3X+ on right side of waist; ActiLife. | 7 days except in water activities; ≥3 weekdays and ≥1 weekend day (10 h/day). For sleep, the criteria were: ≥3 weekdays and ≥1 weekend, with valid data (total  Sleep period time ≥160 min per night and >90% estimated wear time). | Any sequence of ≥20 consecutive  minutes of 0 activity counts. | Sampled at 80 Hz, downloaded in 1-s Epochs, and reintegrated to 15- and 60-s epochs for analysis. | Sleep, SB and MVPA (h/day). | Sleep (sleep period time: sleep onset to sleep offset) defined by algorithm (Tudor-Locke et al. 2014)^106^.  SB (≤25 cp15s), LPA (26–573 cp15s) and  MVPA (≥574 cp15s); Consistent with Evenson et al. 2008^117^. | No wear time was excluded. | No use. |  |
| Ekstedt et al., 2013^20^ | Actiwatch 4 on wrist; N.R. | 7 days, except in water activities; N.R. (N.R.). | sequences during the waking hours  indicated ten or more consecutive minutes of zero counts. | 1-min epoch. | Sleep, SB, LPA, MVPA (min/day). | Sleep (Total sleep time: the sum (in minutes) of all sleep epochs between sleep onset and sleep end) defined by algorithm model (Cole et al., 1992)^120^.  SB (<1.5 METs or < 332 cpm), LPA (1.5 to 3 METs or 332 to 1004 cpm), MPA (3 to 6 METs or 1004 to 2336 cpm) and VPA (> 6 METs or > 2336 cpm); N.R.. | No wear time was excluded from the analyses. | No use. |  |
| Padmapriya et al., 2021^21^ | ActiGraph wGT3X on non-dominant wrist, nonremovable; Actilife. | 7 days, 2 weekdays and 1 weekend day (16h/day). | Non-wear time was estimated based on the standard deviation and value range of each accelerometer axis, using a 60-min window with 15-min increments. | 80 Hz. | Sleep, SB, LPA, MPA and VPA (min/day). | Sleep (Sleep duration) defined by the algorithm (Hees et al., 2018)^121^.  SB (ENMO ≤35 mg), LPA (ENMO 35–200 mg), MPA (ENMO 200 to 707 mg) and VPA (ENMO ≥707 mg); ENMO values were averaged over 5 s epochs, Hildebrand et al., 2014^107^ and 2017^108^. | For each 15-min period detected as non-wear time over the valid wearing days, the invalid data were imputed using the mean value of valid data at same time points on other days. | No use. |  |
| Taylor et al., 2020^22^ | ActiGraph  GT3X on right hip; ActiLife. | 8 days; 3 days (≥20 h/day). | at least 20 min of consecutive zeros during awake time data only. | 15-s  Epoch. | Sleep, SB, LPA, MVPA (% of 24h period). | Sleep duration defined by the algorithm (Meredith-Jones et al., 2016)^122^.  SB (≤25 cp15s), LPA (26–573 cp15s) and  MVPA (≥574 cp15s); Consistent with Evenson et al. 2008^117^. | No wear time was excluded from the analyses. | No use. |  |
| Vuholm et al., 2021^23^ | ActiGraph GT3X +or GT3X on right hip; ActiLife. | 7 days, except in water activities; 3 weekdays and 1 weekend day (≥ 9 waking h/day). | Was removed by the algorithm provided by ActiLife. | 1-min epoch. | Sleep, SB, LPA and MVPA (min day).  TPA (cpm).  Steps (min-1). | Sleep duration defined by the algorithm (Sadeh et al., 1994) ^99^.    SB (≤100 vertical cpm ), LPA (101–2295 vertical cpm) and MVPA (≥2296 vertical cpm); Evenson et al., 2008^117^ | Nights where the monitor was reported to have been removed were excluded from the analyses of sleep as were nights with no recorded activity and nights with a sleep duration ≥ 15 h. | Sleep and non-wear time diary. |  |
| Burkart et al., 2021^24^ | FitBit Charge 2 on wrist; FitBit app and Fitabase plataform | 6 weeks; 3 weekdays and 1 weekend day. (10 waking hours/day) | N.R. | N.R. | Sleep, SB, LPA, MVPA (min/day) | Sleep (Nocturnal sleep: sleep period lasting >240 min with sleep onset between 7 PM and 6 AM, and offset between 5 AM and 1 PM. Other periods, sleep was considered invalid)  PA intensity was calculated from heart rate data: SB (0%-19.9% HRR), LPA (20%-49.9% HRR) and MVPA (≥ 50% HRR); | No wear time was excluded from the analyses. | No use |  |
| Haszard et al., 2020^25^ | Actigraph GT3X on right hip; Actilife | 7 days, except in water activities; 3 days (10 waking h/day and if  the total time was between 20 and 28 hours) | At least 20 min of consecutive zeros. | 15-s epochs. | Sleep, SB, LPA, MVPA (min/day) | Sleep (Sleep period) defined by automated script (Meredith-Jones et al., 2016)^122^.  SB (≤25 cp15s), LPA (26–573 cp15s) and MVPA (≥574 cp15s); Evenson et al. 2008 ^117^. | Non-wear time was reallocated to other day-time components (sedentary, LPA, and MVPA) by  multiplying the proportions of day-time wear by total daytime in minutes. | No use. |  |
| Toledo-Vargas et al., 2020^26^ | ActiGraph GT3X+ on waist; ActiLife. | 7 days, except in water activities; ≥ 4 days, including 1 weekend day (≥10 waking h/day with  their respective sleeping periods). | Filter (Tudor-Locke et al., 2015)^123^. | 1-min epoch. | Sleep and SB (h/day)  MVPA (min/day) | Sleep defined by algorithm (Barreira et al., 2015)^124^.  SB (0.90– 0.98 area under the receiving operating characteristic curve), LPA (0.70 area under the receiving operating characteristic curve), MPA (0.74–0.85 rea under the receiving operating characteristic curve) and VPA (0.83–0.90 rea under the receiving operating characteristic curve); Evenson et al., 2008^117^. | No wear time was excluded from the analyses. | No use |  |
| Armstrong et al., 2021^27^ | Fitbit Charge 2 on non-dominant wrist; Fitabase. | 8 days; N.R. (≥10 waking h/day and with step estimates between 1000 and 30,000 steps) | N.R. | 1-s epoch for PA and 1-min epoch for sleep | Sleep, SB and MVPA (min/day) | Sleep (Nocturnal sleep: >160 consecutive minutes classified as “asleep” between 7:00 pm and 11:58 am).  PA intensity was calculated from heart rate data: SB (0%-19.9% HRR) and MVPA (≥ 50% HRR); Chandler et al., 2016, Gavarry et al., 1998. | No wear time was excluded from the analyses. | No use |  |
| Fairclough et al., 2017^28^ | ActiGraph GT9X on non-dominant wrist; ActiLife | 7 days, except in water activities; 3 days (16 h/day) | Non-wear was estimated on the basis of the standard deviation and value range of each accelerometer axis, calculated for moving windows of 60 min with 15 min increments. | Sampled at  30 Hz and converted in 1-s epoch. | Sleep, SB, LPA and MVPA (min/day). | Sleep defined by the algorithm (Hees et al., 2015)^104^.  SB/LPA (2 METs) and MVPA (4 METs) Hildebrand et al., 2014^107^ | For each 15 min period detected as  non-wear time over the valid days, missing data were replaced by the mean value calculated from measurement on other days at the same time of day. | Non-wear time diary |  |
| Hjorth et al., 2016^29^ | ActiGraph GT3X+ or GT3X on right hip; ActiLife | 7 days except in water activities; ≥3 weekdays and ≥1 weekend day (≥ 10 waking h/day | periods of at least 15 min of consecutive zero counts using tri-axial  vector magnitudes | 1-min epoch | Sleep, SB, LPA. MVPA (min day); TPA (cpm) | Sleep (Sleep duration) was defined by the algorithm (Sadeh et al., 1994)^99^ and diary information.  SB (≤100 vertical cpm), LPA (101–2295 vertical cpm) and MVPA (≥2296 vertical); Trost et al., 2011 | No wear time was excluded from the analyses. | Sleep diary |  |
| Moreno et al., 2021^30^ | Actigraph GT3X-BT on non-dominant wrist; N.R. | 7 days, except water activities; ≥5 nights and ≥4 days, including 1 weekend night and day (≥10 waking hours.  Nights were considered valid if the  participant provided 20 min of wear time before sleep onset. | Defined by diary. | Sampled at 30 Hz and converted in 1-min epoch. | Sleep (h/day), SB, LPA and MVPA (min/day) | Sleep defined by algorithm (Sadeh et al., 1994)^99^.  SB, LPA, MVPA cut points were not clear.  Actigraph’s digital pass filter with a band width of .25 Hz–2.5 Hz, designed to detect normal human behavior. | If epochs of low activity existed  outside of the scoring interval or if non-wear time occurred during the interval, a consensus was reached by the research team.  Non-wear time in the hour before bedtime had to  be less than 60 min unless confirmed by the wear log, or unless ambient light data were available to confirm bedtime. | Sleep and non-wear time diary. |  |
| Antczak et al., 2021^31^ | GENEActiv on non-dominant wrist | 8 days except in organized contact sports; 4 days, including 1 weekend day (>16h/day) | R-package GGIR detects non-wear time | Sampled at 87.5 Hz and converted into 5-second by R-package GGIR | Sleep, SB, LPA, MPA, VPA, MVPA (min/day) | Sleep (duration) defined by algorithm (Hees et al., 2015)^104^.  SB (ENMOZ 0–56.3 mg), LPA (ENMOZ 56.3–191.6 mg), MPA (ENMOZ 191.6–695.8 mg) and VPA (ENMOZ >695.8 mg); Hildebrand et al., 2014^107^ and 2017^108^ | No wear time was excluded from the analyses. | No use. |  |
| García-Alonso et al., 2022^32^ | GENEActiv on non-dominant wrist; GENEActiv PC Software | 6 days; ≥ 6 days (≥600 min/ day in waking time and an average sleep  time ≥ 200 min) | N.R. | Sampled at 87.5 Hz | Sleep (h/day), SB, LPA, MVPA and TPA (min/day) | Sleep defined by algorithm (Hees et al., 2015)^104^.  SB (<56.3 mg); LPA (56.3- 191.5 mg), MPA (191.6-695.7 mg) and VPA (≥ 695.8 mg); Hildebrand et al., 2014^107^ and 2017 ^108^. | No wear time was excluded from the analyses. | Sleep diary. |  |
| Hedayatrad et al. 2022^33^ | Axivity AX3 on dominant thigh and lower back; Open Movement Software | 7 days; 1 day (24h of concurrent wear time for both sensors) | Detected using the in-built temperature sensor.  Schneller et al. 2017^125^ | 5-s epoch; Sampled at 100 Hz converted  into counts. | Sleep, SB, LPA, and MVPA (min/day) | Sleep defined by algorithm (Tudor-Locke et al. 2014)^106^.  SB (N.R.); LPA (N.R.), MVPA (N.R); Evenson et al.2008^117^ | No wear time was excluded from the analyses. | N.R. |  |
| Lucas-de la Cruz et al. 2018^34^ | ActiGraph GMTI on right hip for PA, and on wrist for Sleep; KineSoft software | 7 days except in water activities; ≥4 days including 1 weekend day (≥10 waking h/day). | ≥10 min with zero counts | 30 Hz converted into 60-s epoch | Sleep, SB, LPA, MPA, and VPA (min/day) | Sleep (duration) defined by algorithm (Sadeh et al. 1994)^99^.  SB (0-100 cpm), LPA (101–2295 cpm), MPA (2296–4011 cpm) and VPA (≥4012 cpm); Evenson et al. 2008^117^ | No wear time was excluded from the analyses. | Sleep log |  |
| Martinez et al. 2019^35^ | Actical on right hip; N.R. | 3 days - two weekdays, one weekend day (24h/day) except in water activities | ≥10 min with zero counts during waking time | 60-s epoch | Sleep, SB, LPA, MPA, and VPA (min/day) | Sleep defined as the point when the cpm data changed to consecutive zeros lasting about 8-10 hours,and cross-checked with the participants’ wear log for “off’ times.  SB (AEE <0.01 kcal/kg/min), LPA (AEE 0.01 < 0.04 kcal/kg/min), MPA (AEE 0.04 < 0.10 kcal/kg/min), and VPA (>0.10 kcal/kg/min); Puyau et al. 2004^126^ | No wear time was excluded from the analyses. | Sleep and non-wear time log |  |
| **Adolescents (10-17)** | | | | | | | | | |
| Caetano et al. 2021^36^ | ActiGraph GT3X on right hip; ActiLife | 8 days except in water activities; 5 days including 1 weekend day (≥10 waking h/day). | Zero consecutive counts/min for at  least 20-min. | Sampled at 30 Hz with a standard filter and the data was reintegrated into 15-s epochs. | Sleep (h/day)  SB, LPA, MPA, VPA, MVPA (min/day) | Sleep (sleep duration: evaluation of daily charts and inclinometer data, which were crossed with diary data)  SB (0-180 cp15s), LPA (181-756 cp15s), MPA (757- 1111 cp15s) and VPA (≥1112 cp15s); Romanzini et al. 2014^127^ | No wear time was excluded from the analyses. | Movement behaviours diary. |  |
| Chong et al. 2021^37^ | GENEActiv on non-dominant wrist; GENEActiv PC | 6 days; ≥3 days including 1 weekend day (≥16 h/day) | Non-wear time was estimated based on the standard deviation and value range of each accelerometer axis, calculated over 60 min windows with 15 min increments | At 75 Hz and converted in 5-s epoch | Sleep,  SB, LPA, MVPA (min/day and % of 24h) | Sleep (Sleep duration) defined by the algorithm (Hees et al., 2018)^121^.  SB (ENMO< 52 mg); LPA (ENMO 52–191 mg); MVPA (ENMO ≥ 192 mg); ENMO values were averaged over 5 s epochs, Hildebrand et al., 2014^107^ | For each 15 min period detected as non-wear time over the valid wearing days, missing data were imputed using the mean values calculated from valid data at the same time points on other days.  Any days with ≤200 min of sleep duration or ≤1000 min of ST were considered invalid  and were excluded from the analysis | No use. |  |
| Costa et al., 2020^38^ | ActiGraph GT3x+ on non-dominant wrist; Actilife. | 7 days except in water activities; ≥ 3 weekdays and ≥1 weekend day (≥ 16 h/day). | Identified using an algorithm that inspects blocks of 15 min in windows of 60 min, classifying each block as non-wear if the standard deviation of the moving averaged 60-min window is less than 13 mg and the value range of the 60-min window acceleration  was less than 50 mg for at least two of the three acceleration axes. | Sampled at 30 Hz and data were analyzed in 5 s  epochs. | Sleep, SB, LPA, MVPA (min/day). | Sleep (Sleep duration) defined by the algorithm (Hees et al., 2018)^121^.  SB (< 35.6 mg), LPA (35.6 and 201.4 mg) and MVPA (> 201.4 mg); Hildebrand et al 2014^107^ and 2017^108^. | No wear time was excluded from the analyses. | No use. |  |
| Dumuid et al., 2021^39^ | GENEActiv accelerometer on non-dominant wrist; GENEActiv PC and a MATLAB-based customized software program  (Cobra). | 8 days; 4 days (≥10 waking h/day or ≥200 min/day for sleep or ≥1000 min/d for SB). | N.R. | Data collected at 50 Hz and converted to 60-s epoch. | Sleep, SB, LPA, MVPA (min/day). | Sleep (Sleep duration) defined by the algorithm (Hees et al., 2015)^104^.  SB (244 g.min), LPA (878 g.min) and MVPA (2175 g.min); Philips et al., 2013^128^. | If the device  was removed for “sport”, the corresponding period of non-wear was imputed with 50% MVPA, 30% LPA and 20% sedentary time. | Sleep and non-wear time diary. |  |
| Negele et al., 2020^40^ | ActiGraph GT3X on hip on the side of the dominant hand. At night the accelerometer was moved to the wrist on the non-dominant hand side; ActiLife. | 7 days; 3 weekdays and 1 weekend day (≥10 waking h/ day). | N.R. | Sampled at 30 Hz and the measured accelerations stored at 1 Hz after conversion into proprietary “activity count units” summed over a 1 s epoch.  For PA: 1 min epoch was used. | Sleep, SB and LPA (% of day), MVPA (min/day). | Sleep defined by algorithm (Sadeh et al., 1994)^99^.  SB (<200 cpm), LPA (200 to <3028 cpm), MPA (≥3028 and <4448 cpm), VPA (≥4448); Sasaki et al., 2011^129^. | No wear time was excluded from the analyses. | Sleep and non-wear time diary. |  |
| Tye et al; 2020^130^ | Actigraph GT3X+ on right hip; Actilife. | 7 days, except in water activities; 3 days (10 waking h/day; total wear time or wear time plus imputed physical activity ≥ 20 h; ≥ 2 h of sleep). | Identified by diary. | Sampled at 30 Hz, saved in 15-s epochs and collapsed into 1-min epoch. | Sleep, SB, LPA, MVPA (min/day). | Sleep duration, defined by the algorithm (Sadeh et al., 1994)^99^.  SB (<150 cpm; Kozey-Keadle et al., 2011^131^), LPA (150 to 1951 cpm) and MVPA (≥1952 cpm); Freedson et al., 1998^119^. | Activities that were performed during non-wear time, were also recorded, and then manually imputed (manually imputed data accounted for 0.5% of total wear time). | Sleep and non-wear time diary. |  |
| Van Dyk et al., 2018^41^ | ActiGraph GT3X-BT on waist and SleepWatch on wrist for Sleep. | 5 days; N.R. (≥10 waking h/day). | N.R. | N.R. | Sleep, SB, LPA and MVPA (min/day). | Sleep defined by the algorithm (Sadeh et al., 1994)^99^.  SB (≤100 cpm), LPA (101-2295 cpm) and MVPA (≥2296 cpm); Trost et al., 2011^132^. | No wear time was excluded from the analyses. | Sleep and non-wear time diary. |  |
| Fairclough et al., 2021^41^ | ActiGraph GT9X on non-dominant wrist; Actilife. | 7 days; ≥3 days (16 h/day). | determined based on the SD and value range of the accelerations at each axis, calculated for 60-minute windows with a 15-minute sliding window. If for at least 2 out of the 3 axes the SD was less than 13 mg or the value range was less than 50 mg, the  time window was classified as non-wear (Van Hees et al., 2013)^133^. | Sampled at 100 Hz and converted to 1 omnidirectional measure of acceleration (ENMO), that was reduced to 5-s epoch. | Sleep, SB, LPA, MVPA (min/day) | Sleep (sleep duration) was defined by the algorithm (Hees et al., 2018)^121^.  SB/LPA (ENMO 50 mg; (Hurter et al., 2018)^134^) and MVPA (ENMO 200 mg; Hildebrand et al., 2014)^107^. | Participants were excluded if accelerometer post calibration error was >10 mg.  Non-wear data were imputed by the average at similar time points on other days of the week. | No use. |  |
| Collings et al., 2015^42^ | Actiheart on chest | 4 days;1 day (16 h/day with these hours being roughly  equally distributed between the morning (3 am-9 am),  noon (9 am-3 pm), afternoon (3 pm-9 pm), and midnight  (9 pm-3 am) parts of the day  ) | combination of prolonged periods of  zero acceleration accompanied by non-physiological heart rate data, and data were adjusted to minimize potential diurnal bias during summarization. | 30-s epoch. | Sleep, SB, LPA, MPA and VPA (min/day) | Sleep (1) Sleep onset constitutes the beginning of sustained low movement registration accompanied by a steady decline in heart rate, 2) Sleep termination constitutes the commencement of movement after long periods of barren movement,  together with an abrupt elevation in heart rate. The accumulated time spent in ≤1.5 METs between a designated sleep onset and termination period was deemed to be the sleep duration. This was overlaid on the self-reported sleep habits)  SB (≤1.5 METs), LPA (1.5 to 4 METs), MPA (1.5 to 4 METs) and VPA (>7 METs). | No wear time was excluded from the analyses. | Adolescents were asked to report the times that they usually went to bed and got up on week and weekend days. |  |
| Gába et al., 2020^43^ | ActiGraph GT9X Link or wGT3X-BT on non-dominant wrist; Actilife. | 7 days, except in water activities; ≥4 days, including ≥1 weekend day (≥ 16h/day). | determined based on the SD and value range of the accelerations at each axis, calculated for 60-minute windows with a 15-minute sliding window. If for at least 2 out of the 3 axes the SD was less than 13 mg or the value range was less than 50 mg, the  time window was classified as non-wear (Van Hees et al., 2013)^133^. | Sampled at 100 Hz and converted in 5-s epoch. | Sleep, SB, LPA, MVPA (min/day) | Sleep (the difference between the sleep onset and waking time) was defined by the algorithm (Hees et al., 2015)^104^.  SB (ENMO < 36mg), LPA (ENMO 36–200mg) and MVPA (ENMO ≥201mg); Hildbrand et al., 2014^107^ and 2017 ^119^. | The data missing due to non-wear time or abnormally high acceleration values (0.7% of accelerometer data) were imputed using the accelerometer data from the same time interval on the remaining days of the week. | Sleep and non-wear time diary. |  |
| Kim et al., 2020^55^ | ActiGraph GT9X on non-dominant wrist; ActiLife | 3 days except in water activities; N.R. (≥13 waking h/day) | Choi’s algorithm^135^ in addition to the non-wear time diary | 5-s epoch. | Sleep, SB, LPA, MVPA (min/day) | Sleep (Total sleep time) defined by the algorithm (Sadeh et al., 1994)^99^.  SB (<305 cp15s), LPA (306–817 cp15s), MPA (818–1968 cp15s), VPA (≥1969 cp15s); Chandler et al., 2016^136^, | No wear time was excluded from the analyses. | Non-wear time diary |  |
| Talarico et al., 2018^44^ | Actical on right hip; imported into  SAS statistical software and processed using a specially designed program. | 7 days; ≥ 4 days (≥ 10 waking h/day). | ≥60 consecutive minutes of zero counts, with  allowance of 2 min of counts between 0 and 100. | 15-s epoch. | Sleep, SB, LPA and MVPA (min/day) | Sleep (Sleep duration: manually verified,  and corrected as necessary, by visually inspecting).  SB (< 100 cpm), LPA (100–1499 cpm) and MVPA (> 1499 cpm); Payau et al., 2002^137^. | No wear time was excluded from the analyses. | Activity and sleep diary. |  |
| Starbek et al., 2022^45^ | ActivPAL4 micro on anterior aspect of the right thigh; PAL analysis | 14 days; 6 days (20 h/day). | Defined by diary. | Sampled at 20 Hz. | Sleep, SB, LPA and MVPA (min/day). | Sleep (naps and nighttime duration) was defined by summing activPAL4 output called “primary lying time” (That was set based on entered hours of self-reported SL time) and self-reported napping time.  SB defined by summing activPAL4 output “sitting time” and “secondary lying time”, while deducting self-reported napping time.  LPA defined by residual time to wear time (LPA = 24 h—SL—SB—MVPA—non-wear time).  MVPA (100 steps per minute).  CREA algorithm | If non-wear time existed, the data on  lecture/leisure movement behaviours were proportionally rescaled to fit a specific time period. | Sleep and non-wear time diary. |  |
| Grant et al. 2020^46^ | Actical on wrist; Actical software | 7 consecutive days including sleep- and water-based activities; N.R. (N.R.) | Remotion of the accelerometer | 60-s epochs | Sleep, SB, LPA, MPA and VPA (min/day) | N.R. | Those with a wearing time <168/h were excluded from analysis | Sleep diary |  |
| Beltran-Valls et al., 2021^50^ | GENEActiv, on wrist; GENEActiv Post-Processing PC Software. | N.R.; ≥4 days, including weekends and weekdays (24h/day). | Detected by body temperature sensor. | Sampled at 100 Hz, and converted in 1-s epoch, and data were stored in gravity (g)  units (1 g=9.81 m/s2). | Sleep (hours/day), SB and VPA (min/day). | Sleep defined by algorithm with support of body temperature and luminosity sensors of GENEActiv Post-Processing PC Software.  SB (<7 g), VPA (>60 g,); Phillips et al., 2012^128^. | No wear time was excluded from the analyses. | Sleep and non-wear time diary. |  |
| Domingues et al., 2022^51^ | ActiGraph GT3X on right hip; Actilife. | 8 days except in water activities; ≥ 4 days (10h/day) and ≥ 3 nights (≥ 160 minutes). | At least  20 consecutive minutes of zero counts/minute recording^113^. | Sampled at 30 Hz converted in 1-min epoch. | Sleep, SB, LPA, MVPA (min/day) | Sleep was defined by algorithm (Sadeh et al., 1994)^99^.  SB (≤180 cp15s), LPA ≤757cp15s) and MVPA (≤1112cp15s); Romanzini et al., 2014^127^. | No wear time was excluded from the analyses. | No use. |  |
| Harrington et al., 2021^52^ | GENEActiv; on non-dominant wrist; GENEActiv software | 7 days; N.R. (≥ 16 h/day and < 6 hours or > 12 hours of sleep) | Based on the SD and value range of each axis, calculated for 60-min windows with 15-min moving increments. If, for at least two out of the three axes, the SD is less than 13 mg or the value range is less than 50 mg. Hees et al., 2013^133^ | Sampled at 100 Hz and converted in 5-s epoch | Sleep (h/day), SB, LPA, MVPA (min/day) | Sleep defined by algorithm (Hees et al., 2018)^121^.  SB (0–40 mg (minus sleep)); LPA (41–199 mg); MVPA (≥200 mg); Hildebrand et al., 2014^107^ and 2017^108^ | Files were excluded from all analyses if post-calibration error was greater than 0.02 g or if less than 16 hours of wear time was recorded by either monitor during the 24-hour day of interest. if the sleep duration recorded for a night was < 6 hours or > 12 hours and if visual examination suggested non-wear or erroneous data. | No use. |  |
| Aguilar-Farias et al., 2020^53^ | ActiGraph GT3X+ on right hip; ActiLife | 7 days except in water activities; ≥ 4 days including a weekend day (≥ 10 waking h/d) | 60 consecutive minutes of zero activity counts. | N.R. | Sleep, SB, LPA, MPA, VPA, MVPA (min/day) | Sleep defined by an automated filter (Barreira et al., 2015)^124^  Vertical (uniaxial) axis cut-points:  SB (0-25 cp15s), LPA (26–573 cp15s). MPA (574–1002 cp15s) and VPA ( ≥1003 cp15s); Evenson et al., 2008^117^ | No wear time was excluded from the analyses. | No use. |  |
| Butte et al., 2007^54^ | Actiwatch on right hip; N.R. | 3 days except during water activities; N.R. (≥ 1000 min/day). | If continuous zeros for more than 20 min during awake periods were not accounted for in the subjects log. | 1-min epoch. | Sleep, SB, LPA, MPA, VPA, MVPA (min/day). | Sleep manually screened.  SB (< 1.5 PAR), LPA (1.5 - 2.9 PAR), MPA (3.0-6.0 PAR) and VPA (> 6.0); Puyau et al., 2004^126^. | No wear time was excluded from the analyses. | Sleep and non-wear time diary. |  |
| Krietsch et al. 2022^47^ | ActiGraph wGT3x-BT on dominant hip for PA, and SleepWatch on non-dominant wrist for Sleep; ActiLife. | 7 days (except water-based activities); ≥3 days (≥10 waking/hr  day) | Manufacturer’s algorithm and visually verified by condition-blind study staff | 30-s 1-s epoch  for PA; 60-s 1-s epoch  for Sleep | Sleep, SB, LPA, and MVPA (min/day). | Sleep (duration) defined by algorithm Meltzer et al. 2012^138^.  SB (0-100 cpm), LPA (101-2295 cpm), and MVPA (>2295 cpm)  Trost et al. 2011^132^ | No wear time was excluded from the analyses. | Sleep and non-wear time diary. |  |
| Master et al. 2017^48^ | Actigraph GT3X on hip for PA, and Actiwatch on non-dominant wrist for Sleep; ActiLife and R for PA; Philips Actiware software for Sleep | 7 days (except water-based activities); ≥3 days (≥10 waking/hr  day) | ≥ 90 min of consecutive zero counts | Sampled at 80 Hz and converted in 1-s epoch | Sleep, SB, and MVPA (min/day). | Sleep defined by algorithm (Van Hees et al. 2018)^121^.  SB (<1.5 MET) and MVPA (≥3.8 MET)  Trost et al. 2012^139^ | No wear time was excluded from the analyses. | No use. |  |
| Merikanto et al. 2020^49^ | Actiwatch on non-dominant wrist; N.R. | 7 days (except water-based activities); ≥3 days (≥10 waking/hr  day) | N.R. | 60-s epoch | Sleep, SB, and MVPA (min/day) | Sleep (duration)  SB (0-320 cpm), LPA (321-1047 cpm), and MVPA (≥1048 cpm)  Ekblom et al. 2012^115^ | No wear time was excluded from the analyses. | Sleep log. |  |
| **Adults and older adults (≥ 18 years)** | | | | | | | | | |
| Ataka et al. 2022^56^ | Silmee™ on wrist; N.R. | 7 days except in water activities; at least three days including 1 weekend day (at least three hours). | The time when heart  rate counts were zero. | 1-min epoch. | Sleep duration, SB, LPA, MVPA (min/day). | Sleep (number of minutes asleep between sleep onset and sleep offset: with no movement for more than 20 min; was measured between 6:00 pm and 5:59 am and corrected visually).  SB (≤1.5 METs), LPA (1.5–3.0 METs), MVPA (≥3 METs); algorithms developed by the product designer. | No wear time was excluded from the analyses. | No use. |  |
| Betson et al. 2022^57^ | Actigraph GT9X Link on non-dominant wrist; Actilife | 25 days except in water activities; N.R. (N.R.) | N.R. | 1-min epoch | Sleep (hours/day)  SB and MVPA (time %), and number of steps per day | Sleep defined by the algorithm (Cole-Kripke et al., 1992)^120^  SB (<100 cpm); LPA (≤ 1951 cpm); MVPA (≥1952); Freedson et al 1998^119^ | No wear time was excluded from the analyses. | Sleep log. |  |
| Blodgett et al. 2022^58^ | ActivPAL3 on Midline anterior aspect of the upper thigh; participants were requested not to reattach it; activPAL software | 7 days; 1 day (10 h of waking wear time) | No non-wear time. | Sampled at the default frequency of 20 Hz. | Sleep duration, SB, LPA, MVPA (min/day) | Sleep defined by algorithm (Winkler et al., 2016)^140^.  SB (0 steps), LPA (<100 steps/min), MVPA ≥100 steps/min; Hamer et al., 2020^141^ | Non-compliant participants were excluded from the analyses. | Sleep diary. |  |
| Booth et al. 2012^59^ | Actiwatch-64 with an event marker on non-dominant wrist and the Actical on waist; Actiware Sleep version and Actical | 14 days except in water activities; Actiwatch for at least 7 nightsand the Actical for at least 6 valid days including 1 weekend day (at least 23 h of  recorded data) | N.R. | 1-min epochs | Sleep, SB, LPA, MPA, VPA and MVPA (min/day) | Sleep (Participants were asked to press the event-marking button each night before going to sleep and again when they got out of bed each morning and the data were crossed with sleep log)  SB (subtracted the habitual sleep time from the 24-hour sedentary time of each participant to calculate the amount of habitual sedentary time when he or she was awake.)  LPA (<3.0 METs), MPA (≥3  to <6.0 METs), VPA (≥6.0 METs); (Heil, 2006)^142^ | Non-compliant participants were excluded from the analyses. | Sleep log. |  |
| Buman et al. 2016^60^ | GENEActiv on wrist; Activinsights software package | 3 days; N.R (N.R.) | Periods of non-wear were screened for and removed based upon variability in the monitor temperature outputs and visual inspection | Initially sampled at 40 hz and summarized to 60 s epochs | N.R. | N.R. | Non-compliant participants were excluded from the analyses. | Movement behaviour digital diary. |  |
| Cabanas-Sánchez et al. 2019^61^ | ActiGraph GT9X on non-dominant wrist; ActiLife software. | 7 days except in water activities; ≥4 days including 1 weekend day (at least 16 h/day). | Non-wear time was detected so that each 15-minute block was classified as non-wear time when the standard deviation of 2 out of the 3 axes was lower than 13 mg during the surrounding 1-hour moving window or the value range in 2 of the 3 axes was lower than 50 mg. | Sampled at 100 Hz. | Sleep and SB (h/day).  LPA and MVPA (min/day). | Sleep (sleep onset and offset) defined by the algorithm (Hees et al. 2018)^121^.    SB (ENMO <45 mg); LPA (ENMO ≥45 mg and <100 mg); MVPA (ENMO ≥100 mg); ENMO values were averaged over 5 s epochs, Hildebrand et al., 2017)^108^. | Abnormal high accelerations  and non-wear time were imputed by the mean of the acceleration maintained during the same time intervals as the affected periods for the rest of the recording period in each participant. | No use. |  |
| Carneiro-Barrera et al. 2020^62^ | ActiGraph GT3X+ on non-dominant wrist; ActiLife. | 7 days, except in water activities; ≥ 4 days, including 1 weekend day (16 h/day). | N.R. | Sampled at 100 Hz and converted to 1 s epoch. | Sleep, SB, LPA, MVPA, TPA (min/day). | Sleep (total sleep time: total amount of time spent in bed excluding sleep latency) defined by algorithm (Hees et al 2015)^104^.  SB (ENMO< 52 mg); LPA (ENMO 52–191  mg); MVPA (ENMO ≥  192 mg); ENMO values were averaged over 5 s epochs, Hildebrand et al., 2014^107^. | Non-compliant participants were excluded from the analyses. | Sleep and non-wear time diary |  |
| Dumuid et al., 2018^63^ | ActiGraph GT3X+ on waist; Actilife. | 7 days, except in water activities; ≥ 3 weekdays and ≥1 weekend days (600 min/day of waking wear). | Periods of 60 minutes of consecutive zero counts. | 1-min epoch. | Sleep, SB, LPA and MVPA (min day and % of 24h). | Sleep (Sleep duration: each full day of accelerometer data was then visually inspected by a researcher to determine any discrepancies between self-reported sleep and movement recorded by the device. Where there was discrepancy between the reported times  and the trace, the researcher manually identified the bed and wake times using the trace).  SB (0-99 cpm), LPA (100-1951 cpm), MPA (1952-5274 cpm) and VPA (≥5275 cpm); Freedson et al., 1998^119^. | Non-compliant participants were excluded from the analyses. | Sleep and non-wear times diary. |  |
| Ellingson et al., 2018^64^ | SenseWear Mini Armband on the upper arm (sensors  for heat flux, galvanic skin response, and skin temperature); Brand software. | 10 days, except in water activities; 7 days, including 2 weekend days (≥21 h/day). | Directly measure non-wear time. | 1-min epoch. | Sleep and SB (h/day).  PA (steps/day). | Sleep (total nighttime sleep derived from minute-by-minute sleep epoch).  SB (≤1.5 METs), PA (Average daily steps). | Non-compliant participants were excluded from the analyses. | No use. |  |
| Full et al., 2020^65^ | Actigraph GT3X+ on hip; ActiLife. | N.R.; N.R. (≥16H/day), including the primary sleep period to be considered valid. | N.R. | 30 Hz. Converted in 1-min epoch for sleep and in 15-s epoch for SB and PA. | Sleep, SB, LPA, MVPA (min/day). | Sleep defined by algorithm (Cole-Kripke et al., 1992)^120^.  SB (<19 cp15s); LPA (19–518 cp15s) and MVPA (≥519 cp15s); Evenson et al., 2015^143^  Minutes of in bedtime not classified as sleep by the Cole-Kripke sleep-wake algorithm were classified as minutes of SB, with the low frequency extension filter | Non-compliant participants were excluded from the analyses. | Sleep logs. |  |
| Liao et al., 2020^66^ | Fitbit on wrist; Fitabase. | N.R.; N.R. (≥10 h/day). | Greater than 60 consecutive minutes of 0 steps, with 2 min tolerance (i.e., for 2 min with nonzero counts during non-wear intervals). | 1 min-epoch. | “Minutes asleep” for each sleep episode and “Total minutes asleep” within a day.  Daily total step counts, “Very active minutes”, “Fairly active minutes”, and “Lightly active minutes”. | Valid sleep data were defined as having non-nap sleep duration >3 hr. Nap is a sleep episode with a start time between 8 am to 5 pm. Sleep duration was computed by subtracting total nap minutes from “total minutes asleep.”.  Sedentary minutes variable was computed by subtracting the active and light activity minutes from the total valid wear time.  Active minutes variable was the sum of the “very active” and “fairly active” minutes. | Non-compliant participants were excluded from the analyses. | No use. |  |
| Millard et al., 2021^67^ | Axivity AX 3 on non-dominant wrist; N.R. | 7 days; N.R. (24 h/day). | The UK Biobank accelerometer analysis tool defined the non-wear time (as consecutive stationary episodes (where all 3 axes had a standard deviation of less than 13.0  Milligravities) lasting for at least 60 minutes. | 100 Hz (dynamic range +/− 8 g). The UK Biobank accelerometer analysis tool derives summary activity variables for each 1-minute epoch. | Sleep, SB, LPA, and MVPA (min/day). | Sleep, SB, LPA, and MVPA were defined by a machine learning model (Doherty et al., 2018)^144^.  MVPA was identified as minutes ≥ 100 mg and then the machine learning model was used to identify min of sleep and SB. All other minutes were considered LPA. | Find all periods of accelerometer data on other days that are during the same time period and have no missing data and impute it. | No use. |  |
| Mochón‑Benguigui et al., 2021^68^ | ActiGraph GT3X + on non-dominant wrist; ActiLife. | 7 days, except water activities; 4 days, including 1 weekend day (≥ 16 h/day). | N.R. | Sampled at 100 Hz and converted to 1 s epoch. | N.R. | Total sleep time (total amount of time spent in bed minus sleep onset latency).  SB, LPA, MPA, VPA and MVPA cutoffs were not clarified. | Non-compliant participants were excluded from the analyses. | Sleep and non-wear time diary. |  |
| Park et al., 2022^69^ | ActiGraph GT3X on dominant hip for SB and PA and ActiGraph GT9X on non-dominant wrist for sleep; ActiLife. | 7 days; 4 days (10 h/day). | Cho’s algorithm^135^ to the hip accelerometer data to identify non-wear time, after sleep periods be marked as wear time. | Sampled at 100 Hz, converted in 1-min epoch. | Sleep, SB, LPA, MVPA (min/day) | Sleep duration defined by algorithm (Cole-Kripke et al., 1992)^120^.  SB (≤ 100 cpm), LPA (101–1951 cpm) and MVPA (≥ 1952 cpm); Freedson et al., 1998 ^119^. | Non-compliant participants were excluded from the analyses. | Sleep and non-wear time diary. |  |
| Park et al., 2021^145^ | SenseWear Mini Armband on the back of upper arm (sensors  for heat flux, galvanic skin response, and skin temperature); Brand software. | 1 days, except in water activities (24h). | Directly measured. | 1 min-epoch. | Sleep, SB, LPA, MPA, VPA and MVPA (min/day). | Behaviours were detected by using proprietary pattern recognition algorithms that are refined over time, based on: SB (≤1.5 METs) and MVPA (≥3.0 METs). | Non-compliant participants were excluded from the analyses. | Log to record the duration and types of activities performed while the SWA was not worn. |  |
| Suorsa et al., 2022^70^ | ActiGraph GT3X-BT for SB and PA and ActiSleep-BT for sleep on non-dominant wrist; ActiLife. | 7 days; 4 days (≥10 waking h/day, and no  specific restrictions were made regarding night duration). | 15 min time blocks based on the characteristics of the 60 min time window centered at these 15 min. A block was classified as non-wear time if the standard deviation of the 60 min window was less than 13.0 mg (milli gravity-based acceleration unit, where 1 g = 9.81 m/s2) for at least two out of the three axes or if the value range, for at least two out of three axes, was  less than 50 mg. | Sampled at 80 Hz. | Sleep, SB, LPA, MVPA (proportion of the 24-h day). | Sleep duration, defined by the algorithm and logs (Hees et al., 2015)^104^.  SB (< 30 mg), LPA (30 to 100.6 mg) and MVPA (≥ 100.6 mg); Hildebrand et al., 2014^107^. | Data excluded if <4 days and < 10 waking h/day. | Daily log (record the date, waking time, bedtime and working times) |  |
| Curtis et al., 2020^71^ | GENEActiv on left wrist; N.R. | 7 days, except in water activities; 4 days, including a minimum of 1 weekend day (≥10 waking hours). | Periods of 60 minutes of less than 25 counts per minute were considered nonwear time and excluded from the analysis. | Sampled at 50 Hz, converted in 1-min epoch. | Sleep, SB, LPA, MVPA (min/day; % of time). | Sleep (sleep and wake times were manuallyidentified on the accelerometer trace).  SB (<188 cpm), LPA (188 cpm), MPA (403 cpm), VPA (1131 cpm); Esliger et al., 2011^146^.  If behaviors did not sum to 24 hours, small linear adjustments were made to the daily mean durations so that 100% was equivalent to 24 h for every participant. | Non-compliant participants were excluded from the analyses. | Sleep and non-wear time diary. |  |
| Cabanas-Sanchez et al., 2018^72^ | IDEEA pattern-recognition activity monitor on waist (Three sensors, connected to the main recorder  device by wires, were attached to the anterior sternum and the anterior side of each thigh. Moreover, two sensors were placed on the plantar surface under each foot; these sensors are connected by wires to two subrecorders located on the  lateral side of each ankle which, in turn, are wirelessly connected to the main recorder device). | 2 days, except in water activities; ≥ 1 day (≥10 waking h/day and with the sleep period detected). | N.R. | Sampled at 32 Hz. | Sleep, SB, LPA, MVPA (% of time; h/day). | Sleep (Sleep period: the time while lying, reclining, or sitting during the sleep period) defined by algorithm (Cabanas-Sanchez et al., 2018)^147^.  Lying, reclining, passive sitting, active sitting, standing, walking, and other activities; Cut points were not clear. Walking was divided into two categories according to the 2.5-mph cut point, which identifies light (<2.5 mph) and moderate-to-vigorous (≥2.5 mph) intensities. | Non-compliant participants were excluded from the analyses. | No use. |  |
| Husu et al., 2021^73^ | UKK RM42 on right hip for PA and on non-dominant wrist for sleep | 7 days, except in water activities; ≥4 days (24h) | > 120 min in quiescent time | Sampled at 100 Hz and converted in 6-s epoch | Sleep, SB, standing (h/day), LPA, MPA, VPA (min/day) | SB and standing (MAD < 22.5) LPA (≥ 1.5 and <3.0 (MAD = 22.5 and 91.5 mg)); MVPA (≥ 3.0 (MAD > 91.5 mg)); Vähä-Ypyä et al., 2015 | Non-compliant participants were excluded from the analyses. | Sleep diary. |  |
| Knaeps et al., 2017^74^ | SenseWear Pro 3 Armband on arm; SenseWear Professional software | 7 days, except in water activities; 3 weekdays and 2 weekend days (≥ 1296 minutes) | N.R. | N.R. | Sleep, SB, LPA, MVPA (min/day). | Sleep time was defined by algorithms developed by the manufacturer (Shin et al., 2015)^148^  SB (≤ 1.5 MET), LPA (1.5 – 3 MET) and MVPA (>3 MET); (Shin et al., 2015)^148^. | Non-compliant participants were excluded from the analyses. | No use. |  |
| Mitchell et al., 2016^75^ | ActiGraph GT3X+ on waist (just waking hours) and on non-dominant wrist (24h); Actilife | 7 days, except in water activities; 1 day (the hip acc: ≥ 10 waking hours; Wrist acc: no information) | Choi et al.^135^ algorithm to the hip accelerometer data to identify non-wear time. | N.R. | Sleep and SB (h/day), MVPA (h/day). | Sleep (Total sleep time) defined by algorithm (Cole–Kripke et al., 1992)^120^.  SB (<100 cpm), MVPA (>1040 cpm); Copeland et al., 2009^149^ and Matthews et al., 2008^150^ | Non-compliant participants were excluded from the analyses. | Sleep and non-wear time diary |  |
| Powell et al., 2020^76^ | ActiviPAL3  Micro on right thigh | 7 days, except in water activities; ≥ 4 days, including ≥1 weekday and ≥1 weekend day (≥ 10 waking h/day) | period of ≥ 60 min of consecutive zero counts | Sampled at 20 Hz | Sleep, SB, standing time, LPA, MVPA (% of 24h in min/day) | Sleep (The last registered non-sedentary epoch of the day, which was followed by a long uninterrupted sedentary period (> 2 h), was identified as sleep)  SB and standing time (calculated using the postural function of the monitor, through the proprietary software).  LPA (24h - (SB + standing time + MVPA)  MVPA (≥ 5123 cp15s); Powell et al., 2017^151^ | Non-compliant participants were excluded from the analyses. | Wear time diary |  |
| Gupta et al., 2020^77^ | ActiGraph GT3X+ on thigh; Actilife and MATLAB program Acti4 | 5 days; 1 day (≥4 h of work and leisure and ≥4 of sleep) | (a) periods  longer than 60 min showing zero counts per minute, (b)  workers reported non-wear periods in the diary and (c)  detection of artefacts or missing data via visual inspection of the accelerometer data. | N.R. | Sleep, SB, LPA, MVPA (min/day) | Sleep (time spent in bed) was visually checked and crossed with diaries.  Sitting and/or lying and standing and walking slow (SB and LPA; <100 steps per min) and running, cycling and stair climbing (MVPA; >100 steps per min); Skotte et al., 2014^152^ | Non-compliant participants were excluded from the analyses. | Time  of starting and ending work and going to and out of the  bed each day, time of reference measurement, and non-wear  periods |  |
| Verhoog et al., 2020^78^ | GeneActiv on  non-dominant wrist; PAMPRO processed in Python. | 7 days; ≥4 days (>1,200 min/day). | Time periods where the SD of acceleration in all 3 axes  fell below 13 mg for >1 hour and was excluded from analyses. | Sampled at 50 Hz. | Sleep, SB, LPA, MVPA (min/day). | Sleep (Sleep duration) defined by algorithm (Hees et al., 2015)^104^.  SB (<48 mg), LPA (48−154 mg) and MVPA (>154 mg); White et al., 2016 | Non-compliant participants were excluded from the analyses. | Sleep diary. |  |
| Full et al., 2019^79^ | Actigraph GT3X+, on the hip (not specified, just waking hours) for PA and on the wrist (not specified, 24h) for sleep; Software N.R. | 7 days; ≥5 days (≥10 waking h/day) | 90 consecutives  minutes of zero counts | N.R. | Sleep, SB, and MVPA (min/day) | Sleep (duration) defined by algorithm (Cole-Kripke et al., 1992)^120^.  SB (0–99 cpm), MVPA (≥2020 cpm), Troiano et al. 2008^153^ | Non-compliant participants were excluded from the analyses. | Sleep diary |  |
| Galmes-Panades et al. 2019^80^ | GENEActiv tri-axial on non-dominant  wrist; GGIR package in R | 7 days; ≥3 days (≥10 waking h/day) | N.R. | Sampled at 40 Hz | Sleep, SB, LPA, and MVPA (min/day) | Sleep (Nighttime sleep duration) defined by the algorithm (van Hees et al 2018.)^121^  SB (<40 mg), LPA (48 < 100 mg), and MVPA (≥100 mg). Rowlands et al. 2018^154^ | Non-compliant participants were excluded from the analyses. | Sleep diary |  |
| German et al. 2020^81^ | Actiwatch Spectrum on non-dominant wrist; Actiware-Sleep v.5.59 software | 7 days; ≥3 days (≥10 waking h/day) | N.R. | 30-s epochs | Sleep, SB, LPA, and MVPA (min/day) | Sleep (Nighttime sleep duration) defined by the algorithm (Oakley 1997)^155^.  SB (<178.5 cpm), LPA (178.6–562.4 cpm), and MVPA (>562.4 cpm). Landry et al. 2015^156^. | Participants without 600 daily minutes of wear time  on at least 3 days were excluded from analyses | Sleep diary |  |
| Gilson et al. 2021^82^ | ActiGraph GT3X on non-dominant wrist; GGIR package in R | 7 days; ≥4 days (including at least 1 non-working day) (16h/day) | N.R. | 5-s epochs | Sleep, SB, LPA, MPA and VPA (min/day) | Sleep (Nighttime sleep duration) defined by the algorithm (van Hees et al. 2018)^121^.  SB (<30 mg), LPA (30 < 99 mg), MPA (100 < 399 mg), and VPA (>400 mg); Hildebrand et al. 2014^107^ | Non-compliant participants were excluded from the analyses. | Sleep diary and working non-working diary. |  |
| Goncin et al. 2020^83^ | ActivPAL on right anterior thigh ActivPAL; Open Movement software | 7 days including sleep- and water-based activities; ≥3 days (≥ 10 waking h/day) | 24-h bouts ≥ 2h or any bout ≥ 5 h without detectable movement | Sampled at 20 Hz | Sleep, SB, LPA, MPA and VPA (min/day) | Sleep, SB, LPA, MPA and VPA defined by the algorithm (Winkler et al. 2016)^140^  . | Non-compliant participants were excluded from the analyses. | N.R. |  |
| Gubelmann et al. 2018^84^ | GENEActiv tri-axial on right wrist; GENEActiv software and GGIR package in R | 14 days; ≥7 days (5 weekdays and 2 weekend days; ≥10 and ≥8 waking/h  day on weekdays and weekend days, respectively) | N.R. | Sampled at 50 Hz collapsed into 60-s epochs | Sleep, SB, LPA, and MVPA (min/day) | Sleep (Nighttime sleep duration) defined by algorithm (Hees et al., 2015)^104^.  SB (<241 g/min), LPA (241–338 g min), and MVPA (>338 g min); Esliger et al. 2011^146^ | Non-compliant participants were excluded from the analyses. | Sleep quality derived from the questionnaire. |  |
| Gupta et al. 2018^85^ | ActiGraph GT3X on the right thigh; MATLAB (Acti4) | 4 consecutive days, including ≥2  working days (≥10 waking/h  day) | Defined by diary. | N.R. | Sleep, SB, LPA, and MVPA (min/day) | Sleep (Nighttime sleep ascertained through time in bed).  SB, LPA, and MVPA (N.R.). | Non-working days and non-wear periods of accelerometers  were excluded | Diary on working hours, time in  bed (going to and getting out of bed), and non-wear time |  |
| Hargens et al. 2021^86^ | ActiGraph GT3X on waist for PA and on non-dominant wrist (time in bed) for Sleep; Actilife | 7 days (except water-based activities); ≥3 days (≥8 waking/hr  day) | Wear time was determined by Choi’s algorithm^135^ | Sampled at 30 Hz collapsed into 60-s epochs | Sleep, SB, LPA, MPA, VPA, and VVPA (min/day) | Sleep (duration) defined by algorithm (Sadeh et al. 1994)^99^.  SB (0-99 cpm), LPA (100–1951 cpm), MPA (1952-5724 cpm), VPA (5725–9498 cpm), and VVPA (>9499 cpm); Freedson et al. 1998^119^ | Non-compliant participants were excluded from the analyses. | Sleep log. |  |
| Heiland et al. 2021^87^ | ActiGraph GT3X on waist for PA and on non-dominant wrist (time in bed) for Sleep; Actilife | 8 days (except water-based activities); ≥4 days (≥8 waking/h  day) | ≥60 min with zero counts | Sampled at 30 Hz collapsed into 60-s epochs | Sleep, SB, and MVPA (min/day) | Sleep (duration) defined by algorithm (Cole –Kripke at al. 1992)^120^.  SB (0-199 cpm) and MVPA (>2690 cpm).  SB - Sasaki et al. 2011^129^  MVPA - Aguilar-Farias at al. 2014^157^ | Non-compliant participants were excluded from the analyses. | Sleep log |  |
| Imes et al. 2021^88^ | ActiGraph GT3x on hip for PA and Actiwatch2 on non-dominant hand for sleep; Actilife and Actiware, respectively | 7 days (except water-based activities); ≥4 days (≥10 waking/hr  day) | N.R. | Sampled in 30 Hz collapsed into 60-s epochs | Sleep, SB, and MVPA (min/day) | Sleep (duration) defined by algorithm (Oakley 1997.).  SB (0-199 cpm) and MVPA (>2690 cpm);  SB - Sasaki et al. 2011^129^  MVPA - Aguilar-Farias at al. 2014^157^ | Non-compliant participants were excluded from the analyses. | Sleep log |  |
| Le et al. 2021^89^ | ActiGraph  wGT3X-BT on wrist; ActiLife | 2 days, 7 days, and 15  days (except water-based activities; N.R. (N.R.) | ≥ 90 min  of consecutive zero counts validated by algorithm  Choi et al. 2011^135^ | 60-s epochs | Sleep, SB, LPA and MVPA (min/day) | Sleep (duration) defined by algorithm (Cole–Kripke et al., 1992)^120^.  SB (< 231 cpm), LPA (232–4514 cpm), and MVPA (> 4515 cpm); Lee et al. 2019^158^ | Non-wear time reallocated to other wake components proportionally based on the time they contributed to the total day. | Sleep diary |  |
| Lee et al. 2020^90^ | ActiGraph GT1M on dominant hip for PA, and Actiwatch-2 on non-dominant wrist for Sleep; ActiLife | 7 days (except water-based activities); ≥4 days (≥10 waking/hr  day) | ≥ 60 min  of consecutive zero counts | 60-s epochs | Sleep, SB, LPA, MPA, and VPA (min/day) | Sleep (duration) defined by algorithm (Oakley 1997).  SB (<100 cpm), LPA (100–1951 cpm), MPA (1952-5724), and VPA (≥5725 cpm)  Freedson et al. 1998^119^ | Non-compliant participants were excluded from the analyses. | Sleep diary |  |
| Madden et al. 2014^91^ | SensewearPro for PA and Sleep on arm; R core software package | 7 days; ≥4 days (≥23 wearing h/day) | N.R. | 1-s epochs | Sleep, SB, LPA, and MVPA (min/day) | Sleep (duration) defined by algorithm SenseWear.  SB (0-99 cpm), LPA (100–1951 cpm), and MVPA (≥1952 cpm) Freedson et al. 1998^119^ | Non-compliant participants were excluded from the analyses. | N.R. |  |
| McDonough et al. 2022^92^ | ActiGraph GT9X+ on non-dominant wrist; ActiLife and R Studio-package GGIR | 7 days (except water-based activities); N.R. (N.R.) | N.R. | Sampled in 30Hz collapsed into 5-s epochs | Sleep, SB, LPA, and MVPA (min/day) | Sleep (duration and efficiency) defined by algorithm (Hees et al., 2015)^104^.  SB (0-56.2 mg), LPA (56.3 -191.6 mg), and MVPA (>191.6 mg) Hildebrand et al. 2014^107^. | Intent-to-treat using chained equations (R Studio) | Wearing time and Sleep log |  |
| Meyer et al. 2020^93^ | SenseWear armband on the upper arm; N.R. | 10 days (except water-based activities); ≥5 days, including 2 weekend days (≥18.5 waking/h  day) | N.R. | 60-s epoch | Sleep, SB, LPA and MVPA (min/day) | Sleep (duration) defined by algorithm (SenseWear Sleep algorithm).  SB (1.0–1.5 METs); LPA (1.6–2.9 METs) MVPA (≥3.0 METS). Reece et 2015^159^. | Non-compliant participants were excluded from the analyses. | Activity and Sleep log |  |
| Pasanen et al. 2022^94^ | ActiGraph  wGT3X-BT on non-dominant wrist; R-package GGIR | 8 days/7 nights  (except water-based activities); ≥4 days, including 3 nights (≥10 waking/h  day) | ≥ 60min of <50 mg 2/3 axes or a SD  <13.0 mg | N.R. | Sleep, SB, LPA and MVPA (min/day) | Sleep (duration) defined by algorithm (Van Hees et al. 2015)^104^.  SB (<30.0 mg), LPA (>30.0-100.6 mg), and MVPA (>100.6 mg) Rowlands et al. 2018^154^ | Non-compliant participants were excluded from the analyses. | Daily log (including sleep) |  |
| Seol et al., 2019^95^ | Omrom Active Style Pro HJA-350IT on left side of their waists for PA and SB and actigraph (model not reported) on non-dominant wrist for sleep; Actilife | 7 days; N.R (10 waking h except in water activities) | Intervals of at least  60 consecutive minutes of zero counts, and the total wearing time was computed as  24 h minus non-wearing hours | 1-min epoch. | Sleep, SB, LPA and MVPA (min/day) | Sleep (duration) defined by algorithm (Cole–Kripke et al., 1992)^120^.  SB (1.0–1.5 METs); LPA (1.6–2.9 METs) MVPA (≥3.0 METS), were then scored into units of 30 min per day to facilitate interpretation of the results. | Sleep data  on days where no activity data were measured by the accelerometer  were excluded from the analysis. | Sleep diary |  |
| Tigbe et al., 2017^96^ | ActivPAL on mid anterior thigh; N.R. | 7 days except in water activities; 3 24-hour periods, including a weekend day (N.R.) | Defined by diary | N.R. | Sleep, SB,and standing (h/day)  Stepping (steps/min). | Sleep (duration) was defined as prolonged periods (>2 hours) of continuous inactivity during sleeping hours (extracted from the activPAL raw output)  SB (Sleep duration was subtracted from total sedentary time to obtain waking hours‟ sedentary time.  Stepping and standing (not reported). | Non-compliant participants were excluded from the analyses. | Non wear time diary |  |
| Wang et al., 2022^97^ | ActiGraph GT3X on hip for PA and SB and on the left wrist for sleep | 14 days; 8 days (600 min/day of wear time | N.R. | N.R. | Sleep, SB, LPA, MPA, VPA, Very VPA (% of time) | Sleep duration was recorded by the participants from the time that they began trying to fall asleep till the time they got out of bed in a daily diary.  SB (<1.5 METs), LPA (1.5–3 METs); MPA (3–6 METs); VPA (6–8.9 METs) Very VPA (9 METs); No ref | Non-compliant participants were excluded from the analyses. | Sleep diary |  |
| Santos et al., 2021^98^ | Actigraph GT3X + on dominant wrist; Actilife. | 7 days, except in water activities; 3 weekdays and 1 weekend day (≥10 waking h/day). | N.R. | Sampled at 100 Hz and processed as 10-s epoch. Sleep was analyzed by 1-min epoch. | Sleep, SB, LPA, MPA, VPA and MVPA (h/day). | Sleep (Total sleep time), defined by the algorithm (Cole–Kripke et al., 1992)^120^.  SB (0-275 cp5s), LPA (276-415 cp5s), MPA (≥ 778 cp5s) and VPA (≥ 416 cp5s); Crouter et al., 2015^160^. | No wear time was excluded from the analyses. | Sleep diary |  |

Legend: h: hours; min: minutes; s: seconds; SB: sedentary behavior; PA: physical activity; LPA: light physical activity; MPA: moderate physical activity; VPA: vigorous; MVPA: moderate to vigorous physical activity; TPA: total physical activity; VVPA: very vigorous physical activity; cpm: counts per minute; N.R.: not reported; Hz: Hertz; cp15s: counts per 15 seconds; METs: Metabolic equivalents; mg: milligravitational units; ENMO: Euclidean Norm Minus One; g.min: gravity minutes; g: gravity units; HRR: Heart Rate Reserve; cp5s: counts per 5 seconds; HPFVM: high-pass filtered vector magnitude; PAR: Physical activity ratio; MAD: Mean amplitude deviation.

**References**

1. Armstrong B, Covington LB, Hager ER, Black MM. Objective sleep and physical activity using 24-hour ankle-worn accelerometry among toddlers from low-income families. *Sleep Health*. Oct 2019;5(5):459-465. doi:10.1016/j.sleh.2019.04.005

2. Santos R, Zhang Z, Pereira JR, Sousa-Sá E, Cliff DP, Okely AD. Compliance with the Australian 24-hour movement guidelines for the early years: associations with weight status. *BMC Public Health*. Nov 20 2017;17(Suppl 5):867. doi:10.1186/s12889-017-4857-8

3. Haines J, Douglas S, Mirotta JA, et al. Guelph Family Health Study: pilot study of a home-based obesity prevention intervention. *Can J Public Health*. Aug 2018;109(4):549-560. doi:10.17269/s41997-018-0072-3

4. Alonso-Martínez AM, Ramírez-Vélez R, García-Alonso Y, Izquierdo M, García-Hermoso A. Physical Activity, Sedentary Behavior, Sleep and Self-Regulation in Spanish Preschoolers during the COVID-19 Lockdown. *Int J Environ Res Public Health*. Jan 15 2021;18(2)doi:10.3390/ijerph18020693

5. Chang Z, Lei W. A Study on the Relationship Between Physical Activity, Sedentary Behavior, and Sleep Duration in Preschool Children. *Front Public Health*. 2021;9:618962. doi:10.3389/fpubh.2021.618962

6. Chen B, Bernard JY, Padmapriya N, et al. Associations between early-life screen viewing and 24 hour movement behaviours: findings from a longitudinal birth cohort study. *Lancet Child Adolesc Health*. Mar 2020;4(3):201-209. doi:10.1016/s2352-4642(19)30424-9

7. De Craemer M, Decraene M, Willems I, Buysse F, Van Driessche E, Verbestel V. Objective Measurement of 24-Hour Movement Behaviors in Preschool Children Using Wrist-Worn and Thigh-Worn Accelerometers. *Int J Environ Res Public Health*. Sep 8 2021;18(18)doi:10.3390/ijerph18189482

8. Duraccio KM, Jensen CD. Associations between physical and sedentary activity regularity and sleep in preschoolers and kindergartners. *Sleep Health*. Aug 2017;3(4):263-268. doi:10.1016/j.sleh.2017.04.001

9. Ng JYY, He Q, Chong KH, Okely AD, Chan CHS, Ha AS. The Impact of COVID-19 on Preschool-Aged Children's Movement Behaviors in Hong Kong: A Longitudinal Analysis of Accelerometer-Measured Data. *Int J Environ Res Public Health*. Nov 12 2021;18(22)doi:10.3390/ijerph182211907

10. Tomaz SA, Jones RA, Hinkley T, et al. Physical activity in early childhood education and care settings in a low-income, rural South African community: an observational study. *Rural Remote Health*. Nov 2019;19(4):5249. doi:10.22605/rrh5249

11. Meredith-Jones K, Galland B, Haszard J, et al. Do young children consistently meet 24-h sleep and activity guidelines? A longitudinal analysis using actigraphy. *Int J Obes (Lond)*. Dec 2019;43(12):2555-2564. doi:10.1038/s41366-019-0432-y

12. St Laurent CW, Burkart S, Rodheim K, Marcotte R, Spencer RMC. Cross-Sectional Associations of 24-Hour Sedentary Time, Physical Activity, and Sleep Duration Compositions with Sleep Quality and Habits in Preschoolers. *Int J Environ Res Public Health*. Sep 29 2020;17(19)doi:10.3390/ijerph17197148

13. Williams SM, Farmer VL, Taylor BJ, Taylor RW. Do more active children sleep more? A repeated cross-sectional analysis using accelerometry. *PLoS One*. 2014;9(4):e93117. doi:10.1371/journal.pone.0093117

14. Butte NF, Puyau MR, Wilson TA, et al. Role of physical activity and sleep duration in growth and body composition of preschool-aged children. *Obesity (Silver Spring)*. Jun 2016;24(6):1328-35. doi:10.1002/oby.21489

15. Hossain MS, Deeba IM, Hasan M, et al. International study of 24-h movement behaviors of early years (SUNRISE): a pilot study from Bangladesh. *Pilot Feasibility Stud*. Sep 15 2021;7(1):176. doi:10.1186/s40814-021-00912-1

16. Kang AW, Gans KM, Minkel J, Risica PM. Correlates of Objectively Measured Sleep and Physical Activity Among Latinx 3-To-5-Year Old Children. *J Pediatr Nurs*. Sep-Oct 2021;60:40-45. doi:10.1016/j.pedn.2021.01.010

17. Kuzik N, Naylor PJ, Spence JC, Carson V. Movement behaviours and physical, cognitive, and social-emotional development in preschool-aged children: Cross-sectional associations using compositional analyses. *PLoS One*. 2020;15(8):e0237945. doi:10.1371/journal.pone.0237945

18. Guan H, Zhang Z, Wang B, et al. Proportion of kindergarten children meeting the WHO guidelines on physical activity, sedentary behaviour and sleep and associations with adiposity in urban Beijing. *BMC Pediatr*. Feb 15 2020;20(1):70. doi:10.1186/s12887-020-1969-6

19. Manyanga T, Barnes JD, Chaput JP, Katzmarzyk PT, Prista A, Tremblay MS. Prevalence and correlates of adherence to movement guidelines among urban and rural children in Mozambique: a cross-sectional study. *The international journal of behavioral nutrition and physical activity*. Oct 28 2019;16(1):94. doi:10.1186/s12966-019-0861-y

20. Ekstedt M, Nyberg G, Ingre M, Ekblom Ö, Marcus C. Sleep, physical activity and BMI in six to ten-year-old children measured by accelerometry: a cross-sectional study. *The international journal of behavioral nutrition and physical activity*. Jun 22 2013;10:82. doi:10.1186/1479-5868-10-82

21. Padmapriya N, Chen B, Goh C, et al. 24-hour movement behaviour profiles and their transition in children aged 5.5 and 8 years - findings from a prospective cohort study. *The international journal of behavioral nutrition and physical activity*. Nov 6 2021;18(1):145. doi:10.1186/s12966-021-01210-y

22. Taylor RW, Haszard JJ, Farmer VL, et al. Do differences in compositional time use explain ethnic variation in the prevalence of obesity in children? Analyses using 24-hour accelerometry. *Int J Obes (Lond)*. Jan 2020;44(1):94-103. doi:10.1038/s41366-019-0377-1

23. Vuholm S, Teisen MN, Mølgaard C, Lauritzen L, Damsgaard CT. Sleep and physical activity in healthy 8-9-year-old children are affected by oily fish consumption in the FiSK Junior randomized trial. *Eur J Nutr*. Sep 2021;60(6):3095-3106. doi:10.1007/s00394-021-02490-7

24. Burkart S, Parker H, Weaver RG, et al. Impact of the COVID‐19 pandemic on elementary schoolers' physical activity, sleep, screen time and diet: A quasi‐experimental interrupted time series study. *Pediatric Obesity*. 2022;17(1)doi:10.1111/ijpo.12846

25. Haszard JJ, Meredith-Jones K, Farmer V, Williams S, Galland B, Taylor R. Non-Wear Time and Presentation of Compositional 24-Hour Time-Use Analyses Influence Conclusions About Sleep and Body Mass Index in Children. *Journal for the Measurement of Physical Behaviour*. 2020;3(3):204-210.

26. Toledo-Vargas M, Perez-Contreras P, Chandia-Poblete D, Aguilar-Farias N. Compliance of the 24-Hour Movement Guidelines in 9- to 11-Year-Old Children From a Low-Income Town in Chile. *J Phys Act Health*. Aug 31 2020;17(10):1034-1041. doi:10.1123/jpah.2019-0672

27. Armstrong B, Beets MW, Starrett A, et al. Dynamics of sleep, sedentary behavior, and moderate-to-vigorous physical activity on school versus nonschool days. *Sleep: Journal of Sleep and Sleep Disorders Research*. 2021;44(2):1-12. doi:10.1093/sleep/zsaa174

28. Fairclough SJ, Dumuid D, Taylor S, et al. Fitness, fatness and the reallocation of time between children's daily movement behaviours: an analysis of compositional data. *The international journal of behavioral nutrition and physical activity*. May 10 2017;14(1):64. doi:10.1186/s12966-017-0521-z

29. Hjorth MF, Sørensen LB, Andersen R, et al. Normal weight children have higher cognitive performance—Independent of physical activity, sleep, and diet. *Physiology & Behavior*. 2016;165:398-404. doi:10.1016/j.physbeh.2016.08.021

30. Moreno JP, Razjouyan J, Lester H, et al. Later sleep timing predicts accelerated summer weight gain among elementary school children: a prospective observational study. *The international journal of behavioral nutrition and physical activity*. Jul 12 2021;18(1):94. doi:10.1186/s12966-021-01165-0

31. Antczak D, Sanders T, Del Pozo Cruz B, Parker P, Lonsdale C. Day-to-day and longer-term longitudinal associations between physical activity, sedentary behavior, and sleep in children. *Sleep*. Apr 9 2021;44(4)doi:10.1093/sleep/zsaa219

32. García-Alonso Y, García-Hermoso A, Izquierdo M, Legarra-Gorgoñon G, Ramírez-Vélez R, Alonso-Martínez AM. Relationship between parents' and children's objectively assessed movement behaviours prior to and during the COVID-19 pandemic. *Pediatr Obes*. Sep 2022;17(9):e12923. doi:10.1111/ijpo.12923

33. Hedayatrad L, Stewart T, Paine SJ, Marks E, Walker C, Duncan S. Sociodemographic differences in 24-hour time-use behaviours in New Zealand children. *The international journal of behavioral nutrition and physical activity*. Oct 4 2022;19(1):131. doi:10.1186/s12966-022-01358-1

34. Lucas-de la Cruz L, Martínez-Vizcaíno V, Cañete García-Prieto J, et al. Movement behaviors and cardiometabolic risk in schoolchildren. *PLoS One*. 2018;13(11):e0207300. doi:10.1371/journal.pone.0207300

35. Martinez SM, Tschann JM, McCulloch CE, et al. Temporal associations between circadian sleep and activity patterns in Mexican American children. *Sleep Health*. Apr 2019;5(2):201-207. doi:10.1016/j.sleh.2018.10.012

36. Caetano IT, Miranda VPN, Dos Santos FK, Dos Santos Amorim PR. Adolescent's movement behaviors and built environment: a latent class analysis. *BMC Public Health*. Oct 25 2021;21(1):1937. doi:10.1186/s12889-021-11974-4

37. Chong KH, Parrish AM, Cliff DP, Dumuid D, Okely AD. Cross-Sectional and Longitudinal Associations between 24-Hour Movement Behaviours, Recreational Screen Use and Psychosocial Health Outcomes in Children: A Compositional Data Analysis Approach. *Int J Environ Res Public Health*. Jun 3 2021;18(11)doi:10.3390/ijerph18115995

38. da Costa BGG, Chaput JP, Lopes MVV, Malheiros LEA, Silva KS. How do adolescents with short sleep duration spend their extra waking hours? A device-based analysis of physical activity and sedentary behaviour in a Brazilian sample. *Sleep Sci*. Apr-Jun 2021;14(Spec 2):163-166. doi:10.5935/1984-0063.20200100

39. Dumuid D, Wake M, Burgner D, et al. Balancing time use for children's fitness and adiposity: Evidence to inform 24-hour guidelines for sleep, sedentary time and physical activity. *PLoS One*. 2021;16(1):e0245501. doi:10.1371/journal.pone.0245501

40. Negele L, Flexeder C, Koletzko S, et al. Association between objectively assessed physical activity and sleep quality in adolescence. Results from the GINIplus and LISA studies. *Sleep Med*. Aug 2020;72:65-74. doi:10.1016/j.sleep.2020.03.007

41. Fairclough SJ, Tyler R, Dainty JR, et al. Cross-sectional associations between 24-hour activity behaviours and mental health indicators in children and adolescents: A compositional data analysis. *J Sports Sci*. Jul 2021;39(14):1602-1614. doi:10.1080/02640414.2021.1890351

42. Collings PJ, Wijndaele K, Corder K, et al. Magnitude and determinants of change in objectively-measured physical activity, sedentary time and sleep duration from ages 15 to 175y in UK adolescents: The ROOTS study. *The international journal of behavioral nutrition and physical activity*. 2015;12

43. Gába A, Dygrýn J, Štefelová N, et al. How do short sleepers use extra waking hours? A compositional analysis of 24-h time-use patterns among children and adolescents. *The international journal of behavioral nutrition and physical activity*. Aug 14 2020;17(1):104. doi:10.1186/s12966-020-01004-8

44. Talarico R, Janssen I. Compositional associations of time spent in sleep, sedentary behavior and physical activity with obesity measures in children. *Int J Obes (Lond)*. Aug 2018;42(8):1508-1514. doi:10.1038/s41366-018-0053-x

45. Starbek P, Kastelic K, Šarabon N. The Impact of Online-Schooling during COVID-19 on Device-Measured 24-Hour Movement Behaviours among High School Students: A Compositional Data Analysis. *Children (Basel)*. May 5 2022;9(5)doi:10.3390/children9050667

46. Grant VM, Tomayko EJ, Kingfisher RD. Sleep and Physical Activity Patterns in Urban American Indian Children. *Am J Health Behav*. Jan 1 2020;44(1):67-75. doi:10.5993/ajhb.44.1.7

47. Krietsch KN, Duraccio KM, Zhang N, et al. Earlier bedtimes and more sleep displace sedentary behavior but not moderate-to-vigorous physical activity in adolescents. *Sleep Health*. Jun 2022;8(3):270-276. doi:10.1016/j.sleh.2022.01.003

48. Master L, Nye RT, Lee S, et al. Bidirectional, Daily Temporal Associations between Sleep and Physical Activity in Adolescents. *Sci Rep*. May 22 2019;9(1):7732. doi:10.1038/s41598-019-44059-9

49. Merikanto I, Kuula L, Lahti J, Räikkönen K, Pesonen AK. Eveningness associates with lower physical activity from pre- to late adolescence. *Sleep Med*. Oct 2020;74:189-198. doi:10.1016/j.sleep.2020.07.021

50. Beltran-Valls MR, Adelantado-Renau M, Mota J, Moliner-Urdiales D. Longitudinal Associations of Healthy Behaviors on Fitness in Adolescents: DADOS Study. *Am J Prev Med*. Sep 2021;61(3):410-417. doi:10.1016/j.amepre.2021.04.009

51. Domingues SF, Diniz da Silva C, Faria FR, de Sá Souza H, Dos Santos Amorim PR. Sleep, sedentary behavior, and physical activity in Brazilian adolescents: Achievement recommendations and BMI associations through compositional data analysis. *PLoS One*. 2022;17(4):e0266926. doi:10.1371/journal.pone.0266926

52. Harrington DM, Ioannidou E, Davies MJ, et al. Concurrent screen use and cross-sectional association with lifestyle behaviours and psychosocial health in adolescent females. *Acta Paediatr*. Jul 2021;110(7):2164-2170. doi:10.1111/apa.15806

53. Aguilar-Farias N, Martino-Fuentealba P, Chandia-Poblete D. Correlates of device-measured physical activity, sedentary behaviour and sleeping in children aged 9-11 years from Chile: ESPACIOS study. / Factores asociados con actividad física, conducta sedentaria y sueño medidos con acelerómetros en niños de 9-11 años de Chile: estudio ESPACIOS. *Retos: Nuevas Perspectivas de Educación Física, Deporte y Recreación*. 2020;37:1-10.

54. Butte NF, Puyau MR, Adolph AL, Vohra FA, Zakeri I. Physical activity in nonoverweight and overweight Hispanic children and adolescents. *Med Sci Sports Exerc*. Aug 2007;39(8):1257-66. doi:10.1249/mss.0b013e3180621fb6

55. Kim Y, Umeda M, Lochbaum M, Sloan RA. Examining the day-to-day bidirectional associations between physical activity, sedentary behavior, screen time, and sleep health during school days in adolescents. *PLoS One*. 2020;15(9):e0238721. doi:10.1371/journal.pone.0238721

56. Ataka T, Kimura N, Eguchi A, Matsubara E. Changes in objectively measured lifestyle factors during the COVID-19 pandemic in community-dwelling older adults. *BMC Geriatr*. Apr 14 2022;22(1):326. doi:10.1186/s12877-022-03043-1

57. Betson JR, Kirkcaldie MTK, Zosky GR, Ross RM. Transition to shift work: Sleep patterns, activity levels, and physiological health of early-career paramedics. *Sleep Health*. Oct 2022;8(5):514-520. doi:10.1016/j.sleh.2022.06.001

58. Blodgett JM, Mitchell JJ, Stamatakis E, Chastin S, Hamer M. Associations between the composition of daily time spent in physical activity, sedentary behaviour and sleep and risk of depression: Compositional data analyses of the 1970 British cohort Study. *J Affect Disord*. Sep 30 2022;320:616-620. doi:10.1016/j.jad.2022.09.110

59. Booth JN, Bromley LE, Darukhanavala AP, Whitmore HR, Imperial JG, Penev PD. Reduced physical activity in adults at risk for type 2 diabetes who curtail their sleep. *Obesity (Silver Spring)*. Feb 2012;20(2):278-84. doi:10.1038/oby.2011.306

60. Buman MP, Hu F, Newman E, Smeaton AF, Epstein DR. Behavioral Periodicity Detection from 24 h Wrist Accelerometry and Associations with Cardiometabolic Risk and Health-Related Quality of Life. *Biomed Res Int*. 2016;2016:4856506. doi:10.1155/2016/4856506

61. Cabanas-Sánchez V, Esteban-Cornejo I, Migueles JH, et al. Twenty four-hour activity cycle in older adults using wrist-worn accelerometers: The seniors-ENRICA-2 study. *Scand J Med Sci Sports*. Apr 2020;30(4):700-708. doi:10.1111/sms.13612

62. Carneiro-Barrera A, Amaro-Gahete FJ, Acosta FM, Ruiz JR. Body Composition Impact on Sleep in Young Adults: The Mediating Role of Sedentariness, Physical Activity, and Diet. *J Clin Med*. May 21 2020;9(5)doi:10.3390/jcm9051560

63. Dumuid D, Lewis LK, Olds TS, Maher C, Bondarenko C, Norton L. Relationships between older adults’ use of time and cardio-respiratory fitness, obesity and cardio-metabolic risk: A compositional isotemporal substitution analysis. *Maturitas*. 2018;110:104-110. doi:10.1016/j.maturitas.2018.02.003

64. Ellingson LD, Meyer JD, Shook RP, et al. Changes in sedentary time are associated with changes in mental wellbeing over 1 year in young adults. *Prev Med Rep*. Sep 2018;11:274-281. doi:10.1016/j.pmedr.2018.07.013

65. Full KM, Gallo LC, Malhotra A, et al. Modeling the cardiometabolic benefits of sleep in older women: exploring the 24-hour day. *Sleep*. Jan 13 2020;43(1)doi:10.1093/sleep/zsz205

66. Liao Y, Robertson MC, Winne A, et al. Investigating the within-person relationships between activity levels and sleep duration using Fitbit data. *Transl Behav Med*. Mar 16 2021;11(2):619-624. doi:10.1093/tbm/ibaa071

67. Millard LAC, Tilling K, Gaunt TR, Carslake D, Lawlor DA. Association of physical activity intensity and bout length with mortality: An observational study of 79,503 UK Biobank participants. *PLoS Med*. Sep 2021;18(9):e1003757. doi:10.1371/journal.pmed.1003757

68. Mochón-Benguigui S, Carneiro-Barrera A, Castillo MJ, Amaro-Gahete FJ. Role of physical activity and fitness on sleep in sedentary middle-aged adults: the FIT-AGEING study. *Sci Rep*. Jan 12 2021;11(1):539. doi:10.1038/s41598-020-79355-2

69. Park C, Larsen B, Kwon S, et al. Acculturation, Discrimination and 24-h Activity in Asian American Immigrant Women. *J Immigr Minor Health*. Aug 2022;24(4):1005-1012. doi:10.1007/s10903-022-01361-5

70. Suorsa K, Leskinen T, Pasanen J, et al. Changes in the 24-h movement behaviors during the transition to retirement: compositional data analysis. *The international journal of behavioral nutrition and physical activity*. Sep 15 2022;19(1):121. doi:10.1186/s12966-022-01364-3

71. Curtis RG, Dumuid D, Olds T, et al. The Association Between Time-Use Behaviors and Physical and Mental Well-Being in Adults: A Compositional Isotemporal Substitution Analysis. *J Phys Act Health*. Feb 1 2020;17(2):197-203. doi:10.1123/jpah.2018-0687

72. Cabanas-Sánchez V, Martínez-Gómez D, Izquierdo-Gómez R, Segura-Jiménez V, Castro-Piñero J, Veiga OL. Association between Clustering of Lifestyle Behaviors and Health-Related Physical Fitness in Youth: The UP&DOWN Study. *J Pediatr*. Aug 2018;199:41-48.e1. doi:10.1016/j.jpeds.2018.03.075

73. Husu P, Tokola K, Vähä-Ypyä H, et al. Physical Activity, Sedentary Behavior, and Time in Bed Among Finnish Adults Measured 24/7 by Triaxial Accelerometry. *Journal for the Measurement of Physical Behaviour*. 2021;4(2):163-173.

74. Knaeps S, De Baere S, Bourgois J, Mertens E, Charlier R, Lefevre J. Substituting Sedentary Time With Light and Moderate to Vigorous Physical Activity is Associated With Better Cardiometabolic Health. *J Phys Act Health*. Mar 1 2018;15(3):197-203. doi:10.1123/jpah.2017-0102

75. Mitchell JA, Godbole S, Moran K, et al. No Evidence of Reciprocal Associations between Daily Sleep and Physical Activity. *Med Sci Sports Exerc*. Oct 2016;48(10):1950-6. doi:10.1249/mss.0000000000001000

76. Powell C, Browne LD, Carson BP, et al. Use of Compositional Data Analysis to Show Estimated Changes in Cardiometabolic Health by Reallocating Time to Light-Intensity Physical Activity in Older Adults. *Sports Medicine*. 2020;50(1):205-217.

77. Gupta N, Dencker-Larsen S, Lund Rasmussen C, et al. The physical activity paradox revisited: A prospective study on compositional accelerometer data and long-term sickness absence. *The international journal of behavioral nutrition and physical activity*. 2020;17doi:10.1186/s12966-020-00988-7

78. Verhoog S, Braun KVE, Bano A, et al. Associations of Activity and Sleep With Quality of Life: A Compositional Data Analysis. *Am J Prev Med*. Sep 2020;59(3):412-419. doi:10.1016/j.amepre.2020.03.029

79. Full KM, Moran K, Carlson J, et al. Latent profile analysis of accelerometer-measured sleep, physical activity, and sedentary time and differences in health characteristics in adult women. Article. *PLoS ONE*. 2018;14(6)doi:10.1371/journal.pone.0218595

80. Galmes-Panades AM, Varela-Mato V, Konieczna J, et al. Isotemporal substitution of inactive time with physical activity and time in bed: cross-sectional associations with cardiometabolic health in the PREDIMED-Plus study. *The international journal of behavioral nutrition and physical activity*. Dec 23 2019;16(1):137. doi:10.1186/s12966-019-0892-4

81. German C, Makarem N, Fanning J, et al. Reallocating sedentary behavior with sleep or physical activity is associated with favorable cardiovascular health in the multi-ethnic study of atherosclerosis: MESA. Conference Abstract. *Circulation*. 2020;141(SUPPL 1)doi:10.1161/circ.141.suppl-1.MP16

82. Gilson ND, Mielke GI, Coombes JS, et al. VO(2peak) and 24-hour sleep, sedentary behavior, and physical activity in Australian truck drivers. *Scand J Med Sci Sports*. Jul 2021;31(7):1574-1578. doi:10.1111/sms.13965

83. Goncin N, Linares A, Lloyd M, Dogra S. Does sedentary time increase in older adults in the days following participation in intense exercise? *Aging Clin Exp Res*. Dec 2020;32(12):2517-2527. doi:10.1007/s40520-020-01502-6

84. Gubelmann C, Heinzer R, Haba-Rubio J, Vollenweider P, Marques-Vidal P. Physical activity is associated with higher sleep efficiency in the general population: The CoLaus study. *Sleep: Journal of Sleep and Sleep Disorders Research*. 2018;41(7):1-9. doi:10.1093/sleep/zsy070

85. Gupta N, Dumuid D, Korshøj M, Jørgensen MB, Søgaard K, Holtermann A. Is Daily Composition of Movement Behaviors Related to Blood Pressure in Working Adults? *Med Sci Sports Exerc*. Oct 2018;50(10):2150-2155. doi:10.1249/mss.0000000000001680

86. Hargens TA, Scott MC, Olijar V, Bigman M, Edwards ES. Markers of poor sleep quality increase sedentary behavior in college students as derived from accelerometry. *Sleep Breath*. Mar 2021;25(1):537-544. doi:10.1007/s11325-020-02190-2

87. Heiland EG, Ekblom Ö, Bojsen-Møller E, Larisch LM, Blom V, Ekblom MM. Bi-Directional, Day-to-Day Associations between Objectively-Measured Physical Activity, Sedentary Behavior, and Sleep among Office Workers. *Int J Environ Res Public Health*. Jul 28 2021;18(15)doi:10.3390/ijerph18157999

88. Imes CC, Bizhanova Z, Kline CE, et al. Bidirectional relationship between sleep and sedentary behavior in adults with overweight or obesity: A secondary analysis. *Sleep Adv*. 2021;2(1):zpab004. doi:10.1093/sleepadvances/zpab004

89. Le F, Yap Y, Tung NYC, Bei B, Wiley JF. The Associations Between Daily Activities and Affect: a Compositional Isotemporal Substitution Analysis. *Int J Behav Med*. Aug 2022;29(4):456-468. doi:10.1007/s12529-021-10031-z

90. Lee J, Walker ME, Matthews KA, Kuller LH, Ranjit N, Gabriel KP. Associations of physical activity and sleep with cardiometabolic risk in older women. *Prev Med Rep*. Jun 2020;18:101071. doi:10.1016/j.pmedr.2020.101071

91. Madden KM, Ashe MC, Lockhart C, Chase JM. Sedentary behavior and sleep efficiency in active community-dwelling older adults. *Sleep Sci*. Jun 2014;7(2):82-8. doi:10.1016/j.slsci.2014.09.009

92. McDonough DJ, Helgeson MA, Liu W, Gao Z. Effects of a remote, YouTube-delivered exercise intervention on young adults' physical activity, sedentary behavior, and sleep during the COVID-19 pandemic: Randomized controlled trial. *J Sport Health Sci*. Mar 2022;11(2):145-156. doi:10.1016/j.jshs.2021.07.009

93. Meyer JD, Ellingson LD, Buman MP, Shook RP, Hand GA, Blair SN. Current and 1-Year Psychological and Physical Effects of Replacing Sedentary Time With Time in Other Behaviors. *Am J Prev Med*. Jul 2020;59(1):12-20. doi:10.1016/j.amepre.2020.02.018

94. Pasanen J, Leskinen T, Suorsa K, et al. Effects of physical activity intervention on 24-h movement behaviors: a compositional data analysis. *Sci Rep*. May 24 2022;12(1):8712. doi:10.1038/s41598-022-12715-2

95. Seol J, Abe T, Fujii Y, Joho K, Okura T. Effects of sedentary behavior and physical activity on sleep quality in older people: A cross-sectional study. *Nurs Health Sci*. Mar 2020;22(1):64-71. doi:10.1111/nhs.12647

96. Tigbe WW, Granat MH, Sattar N, Lean MEJ. Time spent in sedentary posture is associated with waist circumference and cardiovascular risk. *Int J Obes (Lond)*. May 2017;41(5):689-696. doi:10.1038/ijo.2017.30

97. Wang R, Blom V, Nooijen CFJ, Kallings LV, Ekblom Ö, Ekblom MM. The Role of Executive Function in the Effectiveness of Multi-Component Interventions Targeting Physical Activity Behavior in Office Workers. *Int J Environ Res Public Health*. Dec 27 2021;19(1)doi:10.3390/ijerph19010266

98. Maria da Silva Santos A, Galan Ribeiro SL, Marreiro de Sousa Junior AV, et al. ARE THERE DIFFERENCES BETWEEN MALE AND FEMALE BADMINTON ATHLETES IN SLEEP, PHYSICAL ACTIVITY AND SEDENTARY TIME? / ¿EXISTEN DIFERENCIAS ENTRE ATLETAS DE BASDMINTON MASCULINOS Y FEMENINOS EN EL SUEÑO, ACTIVIDAD FÍSICA Y TIEMPO DE SEDENTARISMO? *Revista Brasileira de Medicina do Esporte*. 2021;27(2):174-178.

99. Sadeh A, Sharkey M, Carskadon MA. Activity-based sleep-wake identification: an empirical test of methodological issues. *Sleep*. 1994;17(3):201-207.

100. Hager ER, Gormley CE, Latta LW, Treuth MS, Caulfield LE, Black MM. Toddler physical activity study: laboratory and community studies to evaluate accelerometer validity and correlates. *BMC Public Health*. 2016;16(1):1-10. doi:10.1186/s12889-016-3569-9

101. Trost SG, Fees BS, Haar SJ, Murray AD, Crowe LK. Identification and validity of accelerometer cut-points for toddlers. *Obesity (Silver Spring)*. Nov 2012;20(11):2317-9. doi:10.1038/oby.2011.364

102. Choi L, Ward SC, Schnelle JF, Buchowski MS. Assessment of wear/nonwear time classification algorithms for triaxial accelerometer. *Medicine and science in sports and exercise*. 2012;44(10):2009.

103. Butte NF, Wong WW, Lee JS, Adolph AL, Puyau MR, Zakeri IF. Prediction of energy expenditure and physical activity in preschoolers. *Med Sci Sports Exerc*. Jun 2014;46(6):1216-26. doi:10.1249/mss.0000000000000209

104. Van Hees VT, Sabia S, Anderson KN, et al. A novel, open access method to assess sleep duration using a wrist-worn accelerometer. *PloS one*. 2015;10(11):e0142533.

105. Crotti M, Foweather L, Rudd JR, Hurter L, Schwarz S, Boddy LM. Development of raw acceleration cut-points for wrist and hip accelerometers to assess sedentary behaviour and physical activity in 5-7-year-old children. *J Sports Sci*. May 2020;38(9):1036-1045. doi:10.1080/02640414.2020.1740469

106. Tudor-Locke C, Barreira TV, Schuna JM, Jr., Mire EF, Katzmarzyk PT. Fully automated waist-worn accelerometer algorithm for detecting children's sleep-period time separate from 24-h physical activity or sedentary behaviors. *Appl Physiol Nutr Metab*. Jan 2014;39(1):53-7. doi:10.1139/apnm-2013-0173

107. Hildebrand M, VT VH, Hansen BH, Ekelund U. Age group comparability of raw accelerometer output from wrist-and hip-worn monitors. *Medicine and science in sports and exercise*. 2014;46(9):1816-1824.

108. Hildebrand M, Hansen BH, van Hees VT, Ekelund U. Evaluation of raw acceleration sedentary thresholds in children and adults. *Scandinavian journal of medicine & science in sports*. 2017;27(12):1814-1823.

109. Pate RR, Almeida MJ, McIver KL, Pfeiffer KA, Dowda M. Validation and calibration of an accelerometer in preschool children. *Obesity*. 2006;14(11):2000-2006.

110. Chow CM, Wong SN, Shin M, et al. Defining the rest interval associated with the main sleep period in actigraph scoring. *Nature and science of sleep*. 2016:321-328.

111. Janssen X, Cliff DP, Reilly JJ, et al. Predictive validity and classification accuracy of ActiGraph energy expenditure equations and cut-points in young children. *PloS one*. 2013;8(11):e79124.

112. Pate RR, O'Neill JR, Mitchell J. Measurement of physical activity in preschool children. *Medicine and science in sports and exercise*. 2010;42(3):508-512.

113. Esliger DW, Copeland JL, Barnes JD, Tremblay MS. Standardizing and optimizing the use of accelerometer data for free-living physical activity monitoring. *Journal of Physical Activity and health*. 2005;2(3):366-383.

114. Adolph AL, Puyau MR, Vohra FA, Nicklas TA, Zakeri IF, Butte NF. Validation of uniaxial and triaxial accelerometers for the assessment of physical activity in preschool children. *J Phys Act Health*. Sep 2012;9(7):944-53. doi:10.1123/jpah.9.7.944

115. Ekblom O, Nyberg G, Bak EE, Ekelund U, Marcus C. Validity and comparability of a wrist-worn accelerometer in children. *Journal of Physical Activity and Health*. 2012;9(3):389-393.

116. Cliff DP, Reilly JJ, Okely AD. Methodological considerations in using accelerometers to assess habitual physical activity in children aged 0–5 years. *Journal of science and medicine in sport*. 2009;12(5):557-567.

117. Evenson KR, Catellier DJ, Gill K, Ondrak KS, McMurray RG. Calibration of two objective measures of physical activity for children. *Journal of sports sciences*. 2008;26(14):1557-1565.

118. Hjorth MF, Chaput J-P, Damsgaard CT, et al. Measure of sleep and physical activity by a single accelerometer: can a waist-worn Actigraph adequately measure sleep in children? *Sleep and Biological Rhythms*. 2012;10:328-335.

119. Freedson PS, Melanson E, Sirard J. Calibration of the computer science and applications, inc. accelerometer. *Medicine and science in sports and exercise*. 1998;30(5):777-781.

120. Cole RJ, Kripke DF, Gruen W, Mullaney DJ, Gillin JC. Automatic sleep/wake identification from wrist activity. *Sleep*. 1992;15(5):461-469.

121. van Hees VT, Sabia S, Jones SE, et al. Estimating sleep parameters using an accelerometer without sleep diary. *Scientific reports*. 2018;8(1):12975.

122. Meredith-Jones K, Williams S, Galland B, Kennedy G, Taylor R. 24 h Accelerometry: impact of sleep-screening methods on estimates of sedentary behaviour and physical activity while awake. *J Sports Sci*. 2016;34(7):679-85. doi:10.1080/02640414.2015.1068438

123. Tudor-Locke C, Barreira TV, Schuna JM, Jr., et al. Improving wear time compliance with a 24-hour waist-worn accelerometer protocol in the International Study of Childhood Obesity, Lifestyle and the Environment (ISCOLE). *The international journal of behavioral nutrition and physical activity*. Feb 11 2015;12:11. doi:10.1186/s12966-015-0172-x

124. Barreira TV, Schuna Jr JM, Mire EF, et al. Identifying children’s nocturnal sleep using 24-h waist accelerometry. *Medicine & Science in Sports & Exercise*. 2015;47(5):937-943.

125. Schneller MB, Bentsen P, Nielsen G, et al. Measuring children's physical activity: compliance using skin-taped accelerometers. *Medicine & Science in Sports & Exercise*. 2017;49(6):1261-1269.

126. Puyau MR, Adolph AL, Vohra FA, Zakeri I, Butte NF. Prediction of activity energy expenditure using accelerometers in children. *Med Sci Sports Exerc*. Sep 2004;36(9):1625-31.

127. Romanzini M, Petroski EL, Ohara D, Dourado AC, Reichert FF. Calibration of ActiGraph GT3X, Actical and RT3 accelerometers in adolescents. *Eur J Sport Sci*. 2014;14(1):91-9. doi:10.1080/17461391.2012.732614

128. Phillips LR, Parfitt G, Rowlands AV. Calibration of the GENEA accelerometer for assessment of physical activity intensity in children. *J Sci Med Sport*. Mar 2013;16(2):124-8. doi:10.1016/j.jsams.2012.05.013

129. Sasaki JE, John D, Freedson PS, Sasaki JE, John D, Freedson PS. Validation and comparison of ActiGraph activity monitors. *Journal of Science & Medicine in Sport*. 2011;14(5):411-416. doi:10.1016/j.jsams.2011.04.003

130. Tye LS, Scott T, Haszard JJ, Peddie MC. Physical Activity, Sedentary Behaviour and Sleep, and Their Association with BMI in a Sample of Adolescent Females in New Zealand. *Int J Environ Res Public Health*. Aug 31 2020;17(17)doi:10.3390/ijerph17176346

131. Kozey-Keadle S, Libertine A, Lyden K, Staudenmayer J, Freedson PS. Validation of wearable monitors for assessing sedentary behavior. *Med Sci Sports Exerc*. Aug 2011;43(8):1561-7. doi:10.1249/MSS.0b013e31820ce174

132. Trost SG, Loprinzi PD, Moore R, Pfeiffer KA. Comparison of accelerometer cut points for predicting activity intensity in youth. *Medicine & Science in Sports & Exercise*. 2011;43(7):1360-1368.

133. Van Hees VT, Gorzelniak L, Dean León EC, et al. Separating movement and gravity components in an acceleration signal and implications for the assessment of human daily physical activity. *PloS one*. 2013;8(4):e61691.

134. Hurter L, Fairclough SJ, Knowles ZR, Porcellato LA, Cooper-Ryan AM, Boddy LM. Establishing raw acceleration thresholds to classify sedentary and stationary behaviour in children. *Children*. 2018;5(12):172.

135. Choi L, Liu Z, Matthews CE, Buchowski MS. Validation of accelerometer wear and nonwear time classification algorithm. *Med Sci Sports Exerc*. Feb 2011;43(2):357-64. doi:10.1249/MSS.0b013e3181ed61a3

136. Chandler J, Brazendale K, Beets M, Mealing B. Classification of physical activity intensities using a wrist‐worn accelerometer in 8–12‐year‐old children. *Pediatric obesity*. 2016;11(2):120-127.

137. Puyau MR, Adolph AL, Vohra FA, Butte NF. Validation and calibration of physical activity monitors in children. *Obes Res*. Mar 2002;10(3):150-7. doi:10.1038/oby.2002.24

138. Meltzer LJ, Montgomery-Downs HE, Insana SP, Walsh CM. Use of actigraphy for assessment in pediatric sleep research. *Sleep medicine reviews*. 2012;16(5):463-475.

139. Trost SG, Wong W-K, Pfeiffer KA, Zheng Y. Artificial neural networks to predict activity type and energy expenditure in youth. *Medicine and science in sports and exercise*. 2012;44(9):1801.

140. Winkler EA, Bodicoat DH, Healy GN, et al. Identifying adults’ valid waking wear time by automated estimation in activPAL data collected with a 24 h wear protocol. *Physiological measurement*. 2016;37(10):1653.

141. Hamer M, Stamatakis E, Chastin S, et al. Feasibility of measuring sedentary time using data from a thigh-worn accelerometer: the 1970 British Cohort Study. *American Journal of Epidemiology*. 2020;189(9):963-971.

142. Heil DP. Predicting activity energy expenditure using the Actical® activity monitor. *Research quarterly for exercise and sport*. 2006;77(1):64-80.

143. Evenson KR, Wen F, Herring AH, et al. Calibrating physical activity intensity for hip-worn accelerometry in women age 60 to 91 years: The Women's Health Initiative OPACH Calibration Study. *Preventive medicine reports*. 2015;2:750-756.

144. Doherty A, Smith-Byrne K, Ferreira T, et al. GWAS identifies 14 loci for device-measured physical activity and sleep duration. *Nature communications*. 2018;9(1):5257.

145. Park JH, Kim Y, Welk GJ, Silva P, Lee JM. Association with Temperature Variability and Physical Activity, Sedentary Behavior, and Sleep in a Free-Living Population. *Int J Environ Res Public Health*. Dec 11 2021;18(24)doi:10.3390/ijerph182413077

146. Esliger DW, Rowlands AV, Hurst TL, Catt M, Murray P, Eston RG. Validation of the GENEA Accelerometer. *Med Sci Sports Exerc*. Jun 2011;43(6):1085-93. doi:10.1249/MSS.0b013e31820513be

147. Cabanas-Sánchez V, Higueras-Fresnillo S, De la Cámara MÁ, Veiga OL, Martinez-Gomez D. Automated algorithms for detecting sleep period time using a multi-sensor pattern-recognition activity monitor from 24 h free-living data in older adults. *Physiological Measurement*. 2018;39(5):055002.

148. Shin M, Swan P, Chow CM. The validity of Actiwatch2 and SenseWear armband compared against polysomnography at different ambient temperature conditions. *Sleep science*. 2015;8(1):9-15.

149. Copeland JL, Esliger DW. Accelerometer assessment of physical activity in active, healthy older adults. *Journal of aging and physical activity*. 2009;17(1):17-30.

150. Matthews CE, Chen KY, Freedson PS, et al. Amount of time spent in sedentary behaviors in the United States, 2003–2004. *American journal of epidemiology*. 2008;167(7):875-881.

151. Powell C, Carson BP, Dowd KP, Donnelly AE. Simultaneous validation of five activity monitors for use in adult populations. *Scandinavian journal of medicine & science in sports*. 2017;27(12):1881-1892.

152. Skotte J, Korshøj M, Kristiansen J, Hanisch C, Holtermann A. Detection of physical activity types using triaxial accelerometers. *Journal of physical activity and health*. 2014;11(1):76-84.

153. Troiano RP, Berrigan D, Dodd KW, Masse LC, Tilert T, McDowell M. Physical activity in the United States measured by accelerometer. *Medicine and science in sports and exercise*. 2008;40(1):181.

154. Rowlands AV, Mirkes EM, Yates T, et al. Accelerometer-assessed physical activity in epidemiology: are monitors equivalent? 2017;

155. Oakley NR. Validation with polysomnography of the Sleepwatch sleep/wake scoring algorithm used by the Actiwatch activity monitoring system. Mini Mitter Co. *Sleep*. 1997;2:0-140.

156. Landry GJ, Falck RS, Beets MW, Liu-Ambrose T. Measuring physical activity in older adults: Calibrating cut-points for the MotionWatch 8©. *Frontiers in Aging Neuroscience*. 2015;7

157. Aguilar-Farías N, Brown WJ, Peeters GG. ActiGraph GT3X+ cut-points for identifying sedentary behaviour in older adults in free-living environments. *Journal of science and medicine in sport*. 2014;17(3):293-299.

158. Lee P, Tse CY. Calibration of wrist-worn ActiWatch 2 and ActiGraph wGT3X for assessment of physical activity in young adults. *Gait & Posture*. 2019;68:141-149. doi:10.1016/j.gaitpost.2018.11.023

159. Reece JD, Barry V, Fuller DK, Caputo J. Validation of the SenseWear Armband as a Measure of Sedentary Behavior and Light Activity. *J Phys Act Health*. Sep 2015;12(9):1229-37. doi:10.1123/jpah.2014-0136

160. Crouter SE, Flynn JI, Bassett DR, Jr. Estimating physical activity in youth using a wrist accelerometer. *Med Sci Sports Exerc*. May 2015;47(5):944-51. doi:10.1249/mss.0000000000000502
